# Deciphering the Relationship between Obesity and Various Diseases from a Network Perspective

**DOI:** 10.3390/genes8120392

**Published:** 2017-12-18

**Authors:** Lei Chen, Yu-Hang Zhang, JiaRui Li, ShaoPeng Wang, YunHua Zhang, Tao Huang, Yu-Dong Cai

**Affiliations:** 1 School of Life Sciences, Shanghai University, Shanghai 200444, China; chen_lei1@163.com (L.C.); jiaruili@shu.edu.cn (J.L.); wsptfb@163.com (S.W.); 2 College of Information Engineering, Shanghai Maritime University, Shanghai 201306, China; 3 Institute of Health Sciences, Shanghai Institutes for Biological Sciences, Chinese Academy of Sciences, Shanghai 200031, China; zhangyh825@163.com (Y.-H.Z.); tohuangtao@126.com (T.H.); 4 Anhui Province Key Lab of Farmland Ecological Conversation and Pollution Prevention, School of Resources and Environment, Anhui Agricultural University, Hefei 230036, China; yunhua9681@163.com

**Keywords:** obesity, disease gene, OMIM disease class, protein–protein interaction

## Abstract

The number of obesity cases is rapidly increasing in developed and developing countries, thereby causing significant health problems worldwide. The pathologic factors of obesity at the molecular level are not fully characterized, although the imbalance between energy intake and consumption is widely recognized as the main reason for fat accumulation. Previous studies reported that obesity can be caused by the dysfunction of genes associated with other diseases, such as myocardial infarction, hence providing new insights into dissecting the pathogenesis of obesity by investigating its associations with other diseases. In this study, we investigated the relationship between obesity and diseases from Online Mendelian Inheritance in Man (OMIM) databases on the protein–protein interaction (PPI) network. The obesity genes and genes of one OMIM disease were mapped onto the network, and the interaction scores between the two gene sets were investigated on the basis of the PPI of individual gene pairs, thereby inferring the relationship between obesity and this disease. Results suggested that diseases related to nutrition and endocrine are the top two diseases that are closely associated with obesity. This finding is consistent with our general knowledge and indicates the reliability of our obtained results. Moreover, we inferred that diseases related to psychiatric factors and bone may also be highly related to obesity because the two diseases followed the diseases related to nutrition and endocrine according to our results. Numerous obesity–disease associations were identified in the literature to confirm the relationships between obesity and the aforementioned four diseases. These new results may help understand the underlying molecular mechanisms of obesity–disease co-occurrence and provide useful insights for disease prevention and intervention.

## 1. Introduction

Obesity is a common medical condition, which negatively affects health. Obesity and being overweight means that the weight of a person is beyond the prescribed limits that match his or her own height [1]. Adult body mass index (BMI) has been introduced as a unique parameter for the quantitative distinction of such two terms [2,3]. BMI is generally calculated by the weight of a person in kilograms divided by the square of height in meters [3]. If the BMI of a person is more than 30, then this person can be considered obese on the basis of the overweight parameter ranging from 25 to 30. According to the statistics provided by the World Health Organization (WHO) [4] in 2016, the population suffering from obesity has been doubled since 1980. In 2014, more than 600 million adults, which constitutes approximately 13% of all adults, have suffered from obesity, thereby indicating that this disease can be considered a common health issue worldwide [5,6].

The fundamental precipitating factor for the pathogenesis of obesity is the imbalance between energy intake and consumption, thus inducing the accumulation of excessive energy in the form of fat [7]. The increased intake of energy-containing food and lack of physical exercises may be the double burden and major inducer for this energy imbalance-induced pathogenesis, thereby accumulating extra energy-transformed fats. However, the pathological factors for this fat accumulation abnormality are not completely identified. Previous studies confirmed that a high BMI may be a major risk factor for various non-communicable diseases, including cardiovascular diseases, musculoskeletal disorders, and cancers. According to statistical data, the risk of these diseases may be high when the BMI is high; this result indicates the influences of obesity on other diseases [8,9].

Various studies have confirmed the potential pathological contribution of obesity to different diseases subtypes, especially to nutritional and endocrinal diseases. Early in 2014, a systematic study [10] on obesity and its metabolic complications confirmed the potential relationship between obesity and insulin resistance and dyslipidemia. Further, in 2015, obesity has also been functionally connected to another nutritional disease subtype, celiac disease, implying the potential pathogenic contribution of obesity [11]. Apart from nutritional diseases, obesity has also been reported to participate in the pathogenesis of endocrinal disease. In 2015, a clinical study [12] on the hepatic fat distribution confirmed that obesity accompanied with fat accumulation may be functionally connected to a common endocrinal disease, diabetes. Another case report [13] on the pathogenic contribution of obesity implied that obesity may also be associated with Cushing’s and Madelung’s diseases, implying the complicated relationship between obesity and endocrine diseases. As for cardiovascular diseases, *FAIM2* reportedly contributes to the pathological processes of myocardial infarction [9]. Common disease subtypes such as orthopedic diseases, cardiovascular diseases and neoplasms have also been reported to be pathologically related to fat accumulation and obesity, validating the impelling role of obesity in various disease subtypes. Recent publications [9,14] confirmed that *FAIM2* may be a susceptibility gene for fat accumulation and obesity. Considering the pathological role of fat accumulation, a biological process *FAIM2* may participate in, therefore, obesity may demonstrate specific functional relationships with the pathogenesis of myocardial infraction. However, although we indeed summarized that obesity may participate in the pathogenesis of various diseases, it is still not clear whether other diseases may also be related to obesity in some unrevealed ways and how obesity promote such pathogenesis. According to recent publications, detailed biological mechanisms that connect obesity and certain diseases may be attributed to the shared abnormal metabolism biological processes and pathways connected by functional co-relevant genes. Therefore, to identify the detailed contribution of obesity to different disease subtypes, the screening of optimal genes that contribute to both obesity and certain disease subtypes may be an effective means. 

The network method has been proven to be an effective way to analyze pathogenesis of different diseases, especial in disease gene identification [15] because it can integrate various information and investigate problems in a system level. Several methods used the guilt-by-association [16], which always consider the neighbors of the known disease genes in the network as the novel disease genes. In other words, the neighbors of genes related to one disease have strong associations with the disease. Besides, many other methods used some classic network algorithms, such as Random Walk with Restart (RWR) [17], Shortest Path (SP) Algorithm [18], as the basic searching engine to discover novel disease genes [19,20,21,22,23,24,25,26,27]. Based on the known disease genes, the network algorithms were adopted to diffuse them, thereby discovering novel disease genes. It can be seen that the guilt-by-association based method can provide higher accuracies because it always searches novel genes around known ones, while RWR-based or SP-based methods seek novel genes with larger distances. However, the guilt-by-association based method cannot find out genes that are quite different from known ones, while RWR-based or SP-based methods can. Here, we used the guilt-by-association to quantify the relationship between each gene related to one disease and obesity, thereby further determining the associations between the disease and obesity.

In this study, we used a class assignment reported in the study of Goh et al. [28], in which all diseases are clustered into 22 classes [28]. Subsequently, the relevant disease genes that may connect to such diseases were retrieved from Online Mendelian Inheritance in Man (OMIM), USA [29]. In addition, we obtained a group of obesity genes that may contribute to the pathogenesis of fat accumulation and obesity. For each disease, the proteins encoded by each disease gene and by obesity genes were mapped onto the protein–protein interaction (PPI) network retrieved from Search Tool for the Retrieval of Interacting Genes/Proteins (STRING). The interaction with the most robust linkage, which maximum interaction score was used to evaluate the linkage between the disease gene and obesity, was found. The permutation test was further built to evaluate the reliability of this score and reduce the noise of the PPI network that influences the calculation of maximum interaction score, hence resulting in a *p*-value. We proposed a quantitative analysis method based on the distribution of the *p*-values of all disease genes in one disease to measure the relationship between obesity and this disease, thereby extracting the diseases that are highly related to obesity. The results suggested that nutrition- and endocrine-related diseases are mostly related diseases, followed by psychiatric- and bone-related diseases. Several important disease proteins encoded by the genes of the four top diseases were analyzed to further uncover their associations with obesity. For the first time, our study partially revealed the quantitative correlation between obesity and diseases and genetic background of this potential obesity-related pathogenesis, hence establishing further experimental studies.

## 2. Materials and Methods 

### 2.1. Disease Genes of 22 Disease Classes

Several diseases are slightly similar. Thus, diseases with similar features were clustered into one class on the basis of the morbid map file with the Online Mendelian Inheritance in Man (OMIM) disease ID and class assignment reported in the study of Goh et al. [28]. In Goh’s study, a network of disorders and disease genes linked by known disorder-gene associations were presented. All the known disorder-gene associations were drawn from the Morbid Map of OMIM. The disorder clustering was mainly based on the physiological system affected by the disorder. Twenty primary disorder classes were classified manually at first. And another two remaining general categories summarized disorders with distinct multiple clinical features and with insufficient information for classification respectively, thus resulting in 22 disease classes (Column 1 of Table 1). The disease genes of each disease class were retrieved from the OMIM (accessed on January 2014) [14,30]. All disease genes were mapped onto their Ensembl IDs, and those that do not occur in this network were excluded because we used the PPI network reported in STRING to present our investigation. Column 2 of Table 1 lists the number of remaining disease genes for each disease class. Detailed information on remaining disease genes for each class is presented in Table S1.

### 2.2. Obesity Genes

Obesity genes were retrieved from the Human Obesity Gene Map [31], in which 379 obesity genes were curated based on published references with pieces of experimental evidence, such as transgenic or knockout phenotype relevant to obesity. Evidence linking such genes to obesity were obtained from 176 single-gene mutation obesity cases, 50 loci related to Mendelian disorders, transgenic and knockout murine models with 244 independent variants, 408 quantitative trait locis from animal cross-breeding experiments, 426 findings of association studies with candidate genes and linkages from genome scans, summarizing the genetic background of obesity [31]. We obtained 342 Ensembl IDs after mapping the genes onto Ensembl IDs and discarding those not occurring in the PPI network reported in STRING. Detailed information on 379 obesity genes and their Ensembl IDs are available in Table S2.

### 2.3. PPI Network

Several studies reported that proteins that can interact with each other are likely to share similar functions [19,20,21,22,32,33,34,35,36]. Currently, numerous public databases collect PPI information derived from various sources, such as Database of Interacting Proteins [37] and BioGRID [38]. This information is slightly helpful in investigating different protein-related problems. In this study, we aimed to measure the relationships between obesity and 22 disease classes. The access of proteins encoded by obesity genes and disease genes of 22 disease classes is easy. Then, we can infer the associations between obesity and 22 disease classes using the PPI information.

In this study, we adopted the PPI network reported in STRING (version 10.0) [39,40], a popular public database that collects known and predicted PPIs. The PPIs in this database cover 9,643,763 proteins from 2031 organisms and are derived from (1) genomic context predictions, (2) high-throughput laboratory experiments, (3) (conserved) co-expression, (4) automated text-mining, and (5) previous knowledge in databases. Thus, PPIs can measure the associations between proteins extensively. Furthermore, we downloaded the file, “9606.protein.links.v10.txt.gz,” to retrieve the human PPIs to construct the PPI network. This file contains 4,274,001 human PPIs that cover 19,247 human proteins. Each interaction consists of two proteins, represented by Ensembl IDs, and one score ranging between 150 and 999. This score can indicate the strength of the interaction, i.e., the interaction assigned with a high score is likely to occur. For convenience, we denoted the score of the PPI between proteins *p*_1_ and *p*_2_ by *S* (*p*_1_, *p*_2_). Accordingly, the PPI network was constructed by defining proteins as nodes and interactions as edges. Each interaction score was added to the corresponding edge as a weight.

### 2.4. Interaction Method

Section 2.3 discusses that we can use the proteins encoded by the obesity genes and disease genes of 22 disease classes to evaluate the associations between obesity and 22 disease classes. For each disease class, we investigated each disease gene to evaluate its associations to obesity genes. In detail, the disease gene denoted as *g* and the obesity genes were first mapped onto the PPI network mentioned in Section 2.3. Then, this gene was linked to the obesity genes, and the interaction with the highest score was found. This highest score was assigned to *g* as the maximum interaction score, denoted by MIS, which is defined as follows.

MIS(*g*) = max{*S*(*g*, *g*’): *g*’ is an obesity gene} (1)

A disease gene assigned a high MIS means the disease gene is highly related to at least one obesity gene, thereby inducing its strong associations with obesity. 

In this study, we used the PPI network reported in STRING. This network contains considerable noise, which may reduce the reliability of the outcome of Equation (1), although the PPIs in this network can measure the associations between proteins extensively. We performed a permutation test to further evaluate the MIS of each disease gene and increase the reliability of MIS. A total of 1000 Ensembl ID sets were randomly produced, denoted as *D*_1_, *D*_2_, *D*_3_,…, *D*_1000_, in which each set contained many Ensembl IDs similar to those of obesity genes. Then, the MIS of disease gene *g* is calculated for each produced set as follows:

MIS*_i_*(*g*) = max{*S*(*g*, *g*’): *g*’ ∈ *D_i_*} (*i* = 1, 2, …, 1000) (2)

We can further evaluate the reliability of MIS on the obesity gene set for each disease gene by comparing the MISs on 1000 randomly produced sets and MIS on obesity gene set. If the MIS on the obesity gene set of one disease gene is high, and the MISs on the randomly produced sets are also high, then this gene will not be highly related to obesity because this gene constantly has robust linkages with randomly produced gene sets and is indistinct for the obesity gene set. Thus, we computed a measurement, namely, *p*-value, for each disease gene *g* as follows.

(3) $$p\_\mathrm{value}\left(g\right)=\frac{\Lambda }{1000}$$

where Λ represents the number of MISs on randomly produced sets that were higher than the MIS on the obesity gene set. The *p*-value can clearly measure the relative strength of linkages between disease gene and obesity. A disease gene that is receiving a low *p*-value indicates its strong associations to obesity.

### 2.5. Quantitative Analysis Method

Each disease gene of one disease class was assigned a *p*-value through interaction method. We can infer the disease class that is highly related to obesity by a detailed investigation of the *p*-values of the disease genes of each disease class. In this study, we proposed a quantitative analysis method that is slightly similar to the receiver operating characteristic (ROC) curve analysis [41].

The proportion of disease genes receiving *p*-value less than this threshold was computed for each disease class given a threshold of the *p*-value. Then, a proportion–threshold (PT) curve was plotted for each disease class using the threshold of *p*-value as the X-axis and the corresponding proportion as the Y-axis. If a curve follows a sharply increasing trend similar to the ROC curve when the threshold of *p*-value is low, then the corresponding disease class is highly related to obesity. The area under each PT curve was calculated to provide a further quantitative evaluation of the relationship between the disease class and obesity. For convenience, this area was called Area Under Curve (AUC). A disease class that was assigned a high AUC is considered highly related to obesity.

## 3. Results

**Figure 1:**
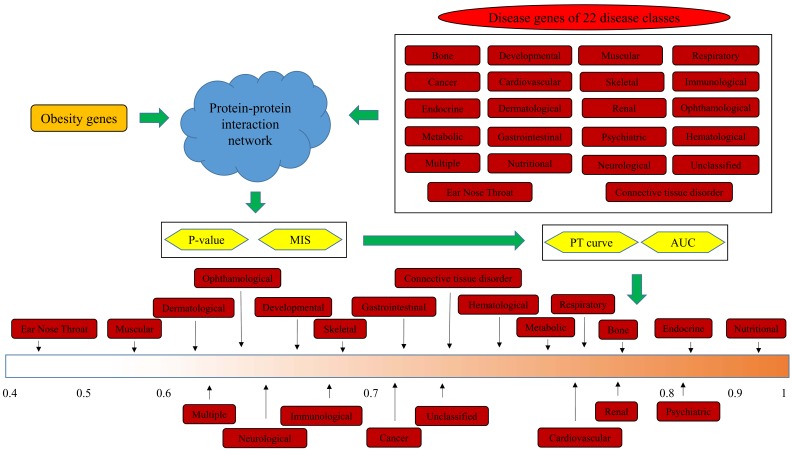
Flowchart of the investigation procedures of this study.

In this study, we inferred the relationship between obesity and 22 disease classes from a network perspective. Figure 1 illustrates the detailed procedures. This section presents the obtained results through the proposed method.

### 3.1. Results of the Interaction Method

According to the interaction method mentioned in Section 2.4, each disease gene in one disease class was first assigned an MIS, thereby indicating its associations with obesity genes. The MISs for all disease genes are provided in Supplementary Materials Table S3. Then, a *p*-value was further calculated for each disease gene; this *p*-value is also provided in Table S3.

### 3.2. Quantitative Analysis Results

**Figure 2:**
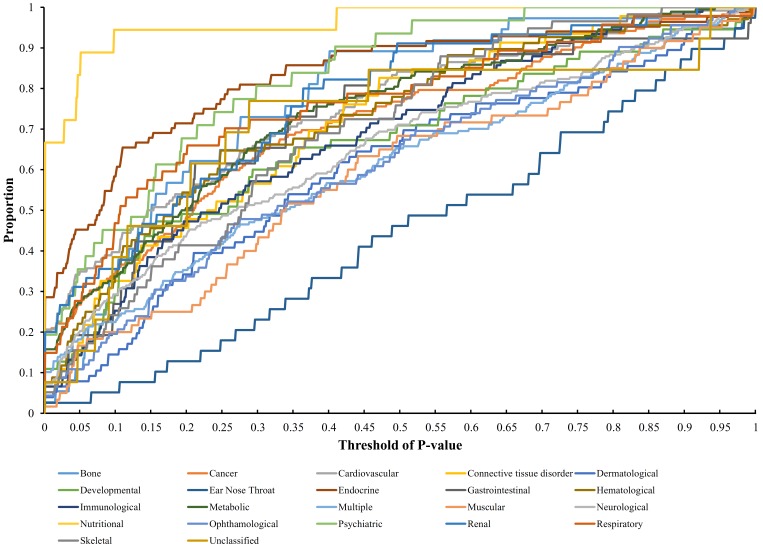
Proportion–threshold (PT) curves for 22 disease classes. X-axis represents the threshold of *p*-value (cf. Equation (3)), and Y-axis denotes the proportion of disease genes receiving the *p*-values less than the threshold.

**Figure 3:**
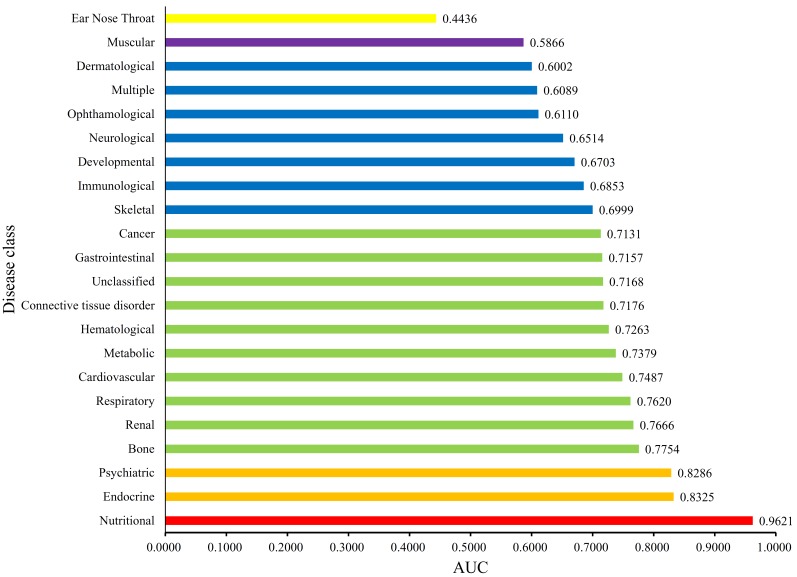
Bar chart of the Area Under Curves (AUCs) of 22 disease classes. The disease class “nutritional” received the highest AUC, thereby indicating that this class is inferred to be highly related to obesity.

Each disease gene was labeled by a *p*-value through the interaction method. The *p*-values of disease genes in one disease class were collected to evaluate the relationship between obesity and 22 disease classes. A series of proportions were obtained for each disease class after setting different thresholds for *p*-value, hence resulting in a PT curve as depicted in Figure 2. We further calculated the AUC for each PT curve to quantify the associations between obesity and 22 disease classes (Figure 3). The disease class “nutritional” received the highest AUC (0.9621), followed by “endocrine” and “psychiatric.” Intuitively, the top two disease classes “nutritional” and “endocrine” are highly related to obesity, thereby indicating the reliability of the results obtained through our method. In addition, the disease classes “psychiatric” and “bone” followed the aforementioned diseases. However, their associations with obesity are unnoticeable, thus providing new insights for connecting the two diseases and obesity.

## 4. Discussion

Obesity has been extensively reported as a health-threatening medical condition for humans, hence increasing the risk for various pathological diseases, including cardiovascular diseases, musculoskeletal disorders, and cancers. In this study, we revealed the quantitative correlation between obesity and 22 disease classes. According to our results, disease classes “nutritional” and “endocrine” are the top two relative diseases of obesity. Obesity, as a contributing and risk factor for the pathogenesis of the two diseases, is reasonable because obesity is induced by nutritional intake and consumption imbalance and may involve various endocrine regulation processes [42,43], thereby validating the efficacy and accuracy of our results. Furthermore, we can find additional pieces of evidence by detailed analyses of the disease genes of the two diseases, partially revealing the potential obesity-associated pathogenesis. In addition, disease classes “psychiatric” and “bone” acquired the third and fourth places, respectively, thereby indicating strong associations with obesity. However, their linkages to obesity are unnoticeable, unlike “nutritional” and “endocrine”. Notably, the following classifications include two specific subgroup named metabolic catalogue and cardiovascular catalogue. According to the classification rule provide by the Goh et al. [28], the general “macroscopic” metabolic catalogue can be detailed divided into nutritional diseases and other nutrition unrelated “metabolic” diseases, due to the different pathogenic emphasis (nutrition related or not). Therefore, such discriminative emphasis of metabolic disease and nutritional disease may lead to their different correlation ship with obesity. Even so, in our prediction list metabolic diseases and another effective category, cardiovascular diseases have also been shown to be related to obesity though ranking behind the optimal classes we have mentioned above. Due to the space limitation, in this study, we only selected the four diseases for our analysis. The analyses on disease genes with *p*-values less than 0.05 can help us uncover the relationship between the disease class and obesity because 0.05 is a widely accepted cutoff on the significance level of the test in statistics. Thus, we removed this type of disease genes of the aforementioned four disease classes and listed them in Table 2, Table 3, Table 4 and Table 5.

### 4.1. Analysis of Disease Genes of the “Nutritional” Class

Among the 22 disease classes, “nutritional” was inferred to have the highest correlation with obesity. Obesity is induced by abnormal energy intake and consuming imbalance; hence, this disease class can be induced as the potential obesity-associated disease subgroup reasonably. For the detailed pathological mechanisms, we can extract 15 functional proteins with *p*-values less than 0.05 according to the results of the interaction method (Table 2), which may mediate the relationships between obesity and the disease class.

NROB2 (ENSP00000254227), as a transcriptional regulator, is generally reported to contribute to steroid hormone metabolisms and co-activation function of the p300/CBP-mediated transcription complex [44,45]. Recent publications confirmed that NROB2-induced fat accumulation may have direct connections with initiating and progressing a nutritional disease, that is, nonalcoholic fatty liver disease [46]. NROB2 has been confirmed to participate in CD11b-mediated obesity-induced insulin resistance during the biological processes of fat accumulation, thereby contributing to the proliferation of adipose tissue macrophages [47] and promoting fat accumulation, resulting in obesity [48]. In addition to NROB2, another protein, namely, SIM1 (ENSP00000262901), is also a potential regulator that contributes to the pathogenesis of the nutritional disease. SIM1 has been reported to be related to appetite disorders, a specific subtype of nutritional diseases, because of its potential contribution to nutritional disease. It contributes to the regulation of human weight and has also been reported to be abnormally inactivated in obesity mouse model [49,50], implying that the inactivation of SIM1 may accompany the initiation and progression of obesity. UCP1 (ENSP00000262999), the uncoupling protein 1, has been confirmed to further participate in the pathogenesis of abnormal gastric emptying and gut hormone release processes, confirming its potential biological functions for nutritional disease [51,52]. Recent studies on energy intake and reproductive output reported that UCP1 participates in a specific biological process, namely, thermogenic respiration in adipose tissue, negatively regulating fat accumulation because of the potential contribution of UCP1 to obesity [53]. POMC (ENSP00000264708) is generally reported to participate in stimulating adrenal glands to release cortisol [54]. The abnormal expression and release of cortisol have been reported to participate in the pathogenesis of zinc metabolism disorders clinically [55]. Furthermore, the release of cortisol and fat accumulation have been reported to be abnormally regulated during the development of obesity [56]. Therefore, the abnormal expression of nutritional disease-associated gene POMC may definitely contribute to obesity [55]. PPARG (ENSP00000287820), a specific steroid hormone coactivator, has also been reported to participate in obesity-associated biological processes [57,58]. In 2015, a specific study on obese people with PPARG variants has confirmed that altered insulin, high low-density lipoprotein, and diastolic blood pressure are risk phenotypes that are induced by PPARG in obese populations [59]. PPARG becomes a functional nutritional disease-associated protein because these phenotypes (altered insulin level, high low-density lipoprotein, and abnormal blood pressure) are potential phenotypes for various nutritional diseases [60]. Therefore, obesity may directly contribute to the pathogenesis of a certain nutritional disease, and partially attributing the contribution of obesity to nutritional disease to PPARG is reasonable.

### 4.2. Analysis of Disease Genes of the “Endocrine” Class

**Figure 4:**
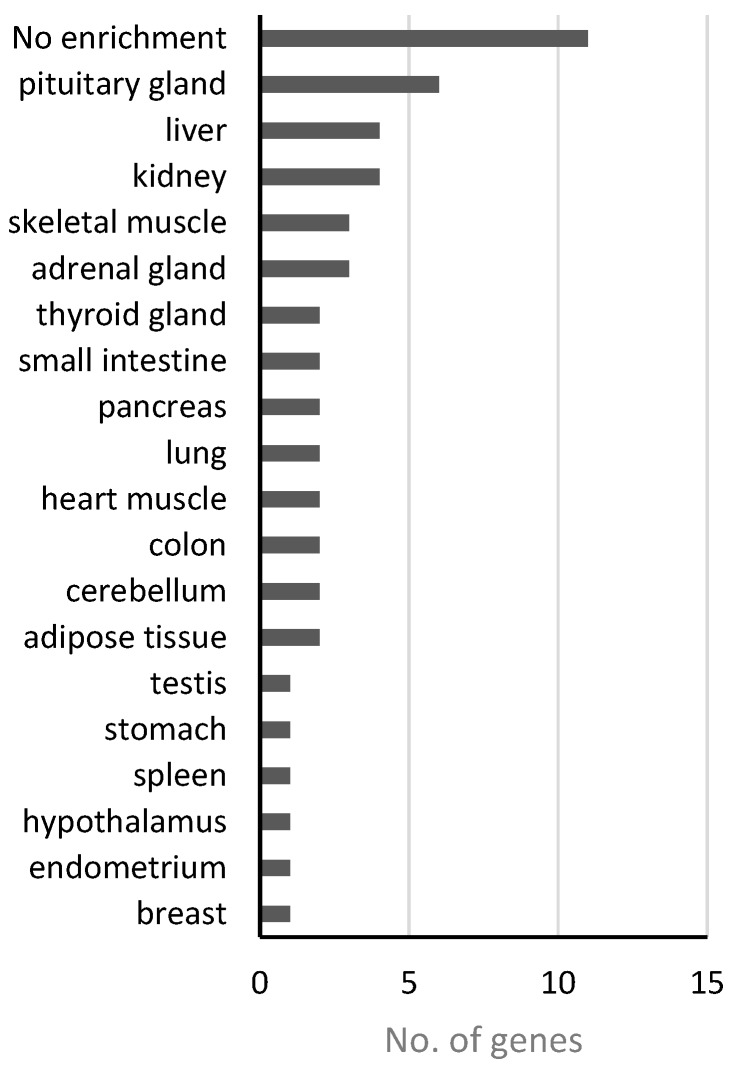
Gene expression enrichment in tissue/organ of 38 “endocrine”-class genes based on GTEx.

In addition to “nutritional,” obesity has also been inferred to promote the pathogenesis of “endocrine” disease class at the genetic level. Obesity, as an energy metabolism abnormality, may have potential relationships with the effective system in humans because the endocrine system contains various energy metabolism-regulating hormones. For the disease genes of this class, 38 functional genes received *p*-values less than 0.05 (Table 3), which may link obesity to this disease. We investigated the expression patterns of these genes across different tissues through the online databases from the Human Protein Atals [61], the Genotype-Tissue Expression (GTEx) [62] and FANTOM5 project [63], and obtained the tissues/organs where those 38 genes were significantly enriched (Table S4). Figure 4 shows the numbers of genes enriched in different tissues or organs based on genes expression levels from GTEx database, which has the fewest missing data comparing with the other two. Among 38 genes, 11 are not enriched in any tissue but widely-expressed in most or all of the tissues. However, we can see 6, 3, and 2 genes enriched in pituitary gland, adrenal gland and thyroid gland, respectively. Meanwhile, other tissues/organs such as liver, pancreas, and testis are also involved in endocrine functions, and the hypothalamus is well-known as the neural control center of endocrine system in human. These results demonstrated again the connection between endocrine system and obesity.

RETN (ENSP00000221515) is a functional hormone that mediates the insulin capability to stimulate glucose uptake into adipose cells, thereby contributing to the normal regulation of endocrine functions [64,65]. Secreted by adipocytes, the protein coding by RETN may link obesity to type II diabetes considering its functional biological functions on regulating insulin activity and biological source [66]. Therefore, obesity may be connected to the pathogenesis of endocrine disease via specific predicted genes, such as RETN. HGF (ENSP00000222390) has also been induced to reveal the linkage between obesity and endocrine diseases. Insulin resistance, as an endocrine-regulating abnormality, is directly related to various endocrine diseases, including diabetes [67,68]. Furthermore, HGF acts as a growth factor for a broad spectrum of tissues and cell types and is extensively reported to participate in regulating high-fat-diet-induced obesity and improve insulin resistance in mice [69,70]. In summary, obesity may also contribute to the pathogenesis of endocrine diseases. GCK (ENSP00000223366), a glucokinase, is also identified to link obesity to endocrine diseases. The abnormal biological function of such protein, as a mitochondria-locating energy-regulating gene, is widely reported in obese populations, indicating its specific biological functions for obesity occurrence [71,72]. Recent publications further confirmed that GCK may be associated with non-insulin-dependent diabetes mellitus [73,74], maturity-onset diabetes of the young, type 2 [74], and persistent hyperinsulinemic hypoglycemia of infancy [75], thereby validating the potential biological contributions of obesity to this endocrine pathogenesis. The protein, INS (ENSP00000250971), encodes the famous glucose homeostasis-regulating hormone insulin. INS regulates hormone stability and is also reported to contribute to obesity, confirming the potential relationship between obesity and endocrine diseases [76,77]. AVP (ENSP00000369647) as the following predicted gene has been confirmed to be abnormally hyperactivity in obesity [78]. Further study on the effective role of AVP in endocrine disease confirmed that such gene may be associated with specific syndrome induced by inappropriate antidiuretic hormone secretion (SIADH) [79], validating the co-related role of such gene in multiple pathogenesis. 

### 4.3. Analysis of Disease Genes of the “Psychiatric” Class

According to our results, psychiatric disease class is the third most related disease to obesity. Eleven disease genes of this class were assigned the *p*-values of less than 0.05, as listed in Table 4. Most of those genes have no enrichment in expression pattern across tissues, while four are enriched in central nervous system (Table S4), suggesting the potential role of those genes in psychiatric disorders. The analysis of several of these genes can validate the potential pathogenic connections between obesity and psychiatric disease. 

The first candidate, DRD4 (ENSP00000176183), is a G-protein coupled receptor that contributes to the neuronal signaling in the mesolimbic system of the brain that regulates emotion and behavior [80,81]. In addition, DRD4 is clustered as dopamine proteins and is reported to reveal the potential relationship between weight gain, obesity, and seasonal affective disorder [82] not only confirming the potential biological connections between obesity and psychiatric disease but also validating the regulator role of DRD4 during this pathogenesis. Functional gene SLC6A4 (ENSP00000270349) has also been predicted to be a correlated gene of obesity and psychiatric disease. Early in 2015, a specific study [83] on the serotonin transporter confiremd that our predicted gene SLC6A4 participate in processing emotional stimuli and abnormal brain function in pathogenic status. Another study [84] in 2016 implied that our predicted gene which also known as 5-HTT may be related to the obesity of Portuguese origin, validating our prediction. Moreover, APOL2 (ENSP00000249066) is a regulator for the movement of lipids in the cytoplasm and the binding of lipids to organelles [85,86]. In 2008, a GWAS study [87] on schizophrenia, a typical psychiatric disease, has confirmed that APOL2 may also contribute to the pathogenesis of schizophrenia given its potential contribution to psychiatric diseases. In addition to its lipid-regulating role, this gene is reported to contribute to a reduction in weight gain, stored fat, and circulating lipids, with a low expression profile in the obese population [88], thus confirming the potential contribution of APOL2 and obesity to the pathogenesis of psychiatric diseases. Then, CHI3L1 (ENSP00000255409) is generally reported as a carbohydrate-binding lectin with a preference for chitin [89]. YKL-40 is a pro-inflammatory glycoprotein regulated by CHI3L1 in obese populations and is extensively reported to contribute to the progression of Alzheimer’s disease given its contribution to psychiatric diseases [90,91]. Coincidentally, CHI3L1 and chitin enhances obese inflammation and upregulates the plasma levels of pro-inflammatory glycoprotein YKL-40 according to recent publications [89,92], hence revealing the specific inflammatory regulatory role of CHI3L1 in obese populations and validating the potential relationships between obesity and psychiatric diseases mediated by CHI3L1. COMT (ENSP00000354511) has also been predicted to be a co-related gene for both obesity and psychiatric diseases. A case study [93] in 2015 validated that the polymorphisms of COMT may be correlated with obesity occurrence. Further study on COMT confirmed that such gene turns out to be one of the potential pathogenic gene for Parkinson disease, validating the efficacy and accuracy of our prediction.

### 4.4. Analysis of Disease Genes of the “Bone” Class

According to our results, the disease class “bone” was the fourth obesity-related disease. Four disease genes received *p*-values less than 0.05, as listed in Table 5. All of these genes are widely expressed in most or all tissues (Table S4). The analyses on these genes can partly uncover the relationship between “Bone” and obesity. 

ANKH (ENSP00000284268) is a multipass transmembrane protein that mainly participates in regulating intra- and extracellular levels of inorganic pyrophosphate (PPi) as functional transporter [94]. Ankylosing spondylitis is a type of arthritis that involves the inflammation of the joints of the spine, hence involving the bone system [95]. ANKH is a specific bone disease-associated gene [96] and is also reported to participate in the biological processes of obesity, thereby corresponding to our results [97]. Obesity may also be linked to specific bone diseases through various functional genes, including ANKH, considering the specific contribution of ANKH in bone disease and fat accumulation. TNFRSF11B (ENSP00000297350) is a member of the TNF receptor superfamily, involving in osteoclastogenesis [98,99]. In 2016, TNFRSF11B was reported to participate in obesity-induced metabolic bone disease of women given its detailed relationship with bone disease [100]. Furthermore, a specific study on obesity-mediated inflammatory microenvironment in 2011 validated the specific contribution of TNFRSF11B as a bone disease-associated gene on the pathogenesis of obesity [101]. ALPL (ENSP00000363965) is a member of the alkaline phosphatase family of proteins, which participate in skeletal mineralization in bones [102,103]. The polymorphisms of mineralization genes, such as ALPL, are reported to be directly related to obesity according to recent publications, hence indicating the accompanying role of ALPL for obesity [97]. EXT1 (ENSP00000367446), encoding a member of transmembrane glycosyltransferase has been reported to participate in the obesity associated biological processes in the Lebanese Arab population [104]. As for its contribution on bone diseases, in 2013, a mutation screening [105] for EXT1 in Chinese population confirmed that such gene may be functional related with a specific bone disease, osteochondromas, validating the efficacy and accuracy of our prediction.

Based on the analyses, certain diseases of the four disease classes are highly related to obesity, thereby further inferring the close associations between these diseases and obesity. This study suggested that “psychiatric” and “bone” classes are obesity-related diseases, which may provide new insights for investigating their pathogenesis.

## 5. Conclusions

This study provided an investigation on the relationship between obesity and 22 disease classes. The relationship between the disease class and obesity was evaluated by the linkages between proteins encoded by the disease genes and obesity genes by using the PPI network reported in STRING. The results indicated that “nutritional” and “endocrine” were most related to obesity; these results are consistent with our general knowledge. In addition, “psychiatric” and “bone” classes followed the abovementioned two diseases, which do not obviously relate to obesity. However, further analysis uncovered their potential associations with obesity. Our new finding aims to provide new insights to investigating obesity-related diseases.

## Figures and Tables

**Table 1:** Number of disease genes of 22 Online Mendelian Inheritance in Man (OMIM) disease classes.

OMIM Disease Class	Number of Disease Genes
Bone	38
Cancer	172
Cardiovascular	126
Connective tissue disorder	46
Dermatological	76
Developmental	55
Ear Nose Throat	39
Endocrine	84
Gastrointestinal	26
Hematological	68
Immunological	91
Metabolic	184
Multiple	187
Muscular	60
Neurological	232
Nutritional	18
Ophthamological	92
Psychiatric	31
Renal	45
Respiratory	47
Skeletal	58
Unclassified	14

**Table 2:** Information on important disease genes of “nutritional”.

Gene Symbol	MIS	*p*-Value
*UCP1*	969	<0.001
*POMC*	998	<0.001
*PPARG*	999	<0.001
*AGRP*	999	<0.001
*MC4R*	999	<0.001
*PCSK1*	970	<0.001
*PPARGC1B*	978	<0.001
*UCP3*	960	<0.001
*GHRL*	994	<0.001
*ADRB3*	964	<0.001
*PYY*	993	<0.001
*ENPP1*	979	<0.001
*NR0B2*	993	0.027
*SIM1*	879	0.044
*HTR2A*	949	0.046

MIS: Maximum Interaction Score.

**Table 3:** Information on important disease genes of “endocrine”.

Gene Symbol	MIS	*p*-Value	Gene Symbol	MIS	*p*-Value
*RETN*	981	<0.001	*FSHB*	999	<0.001
*PPP1R3A*	954	<0.001	*CYP11B1*	973	<0.001
*NEUROD1*	994	<0.001	*IRS1*	999	<0.001
*MC2R*	998	<0.001	*KCNJ11*	999	<0.001
*CYP17A1*	994	<0.001	*AR*	999	<0.001
*AVP*	997	<0.001	*ABCC8*	999	<0.001
*INS*	999	<0.001	*IL6*	999	<0.001
*PPARG*	999	<0.001	*STAT5B*	999	<0.001
*LIPC*	984	<0.001	*HNF4A*	999	<0.001
*AVPR2*	804	<0.001	*ENPP1*	979	<0.001
*PTPN1*	999	<0.001	*IRS2*	999	<0.001
*IGF2BP2*	989	<0.001	*CAPN10*	923	<0.001
*SSTR5*	968	0.013	*PAX8*	954	0.014
*GCK*	989	0.017	*TBX19*	937	0.017
*TSHR*	980	0.017	*SLC2A4*	994	0.026
*PAX4*	925	0.03	*STAR*	942	0.032
*HGF*	992	0.033	*BMP15*	699	0.034
*HNF1A*	993	0.036	*GPD2*	924	0.038
*HMGA1*	984	0.041	*GCGR*	960	0.043

**Table 4:** Information on important disease genes of “psychiatric”.

Gene Symbol	MIS	*p*-Value
*DRD4*	972	<0.001
*SLC6A4*	961	<0.001
*AKT1*	999	<0.001
*APOL4*	742	<0.001
*COMT*	981	<0.001
*BDNF*	999	<0.001
*SLC6A3*	984	0.017
*CHI3L1*	842	0.024
*APOL2*	662	0.035
*HCRT*	967	0.043
*HTR2A*	949	0.046

**Table 5:** Information on important disease genes of “bone”.

Gene Symbol	MIS	*p*-Value
*ALPL*	969	<0.001
*ANKH*	610	0.015
*TNFRSF11B*	895	0.034
*EXT1*	968	0.043

## Supplementary Materials

The following are available online at www.mdpi.com/2073-4425/8/12/392/s1. Table S1: Disease genes of 22 OMIM disease classes and their Ensembl IDs, Table S2: Gene symbols of 379 obesity genes and their Ensembl IDs, Table S3: Maximum interaction scores and *p*-value for each disease gene of 22 disease classes, Table S4: The tissues/organs where genes related to “Endocrine”, “Psychiatric” and “Bone” were significantly enriched.
