# Supplementary material for: Deciphering the Relationship between Obesity and Various Diseases from a Network Perspective
Source: Genes (Basel). 2017 Dec 18;8(12):392. doi: 10.3390/genes8120392 (PMC5748710; doi:10.3390/genes8120392)
Supplement: Supplementary file 1 [file genes-08-00392-s001.zip › Table_S1.docx]

**Table S1.** Disease genes of 22 OMIM disease classes.

1. Disease genes of bone

| **Gene symbol** | **Ensembl ID** |
| --- | --- |
| ALPL | ENSP00000363965 |
| ANKH | ENSP00000284268 |
| TNFRSF11B | ENSP00000297350 |
| EXT1 | ENSP00000367446 |
| EIF2AK3 | ENSP00000307235 |
| EXT2 | ENSP00000379032 |
| CYP2R1 | ENSP00000334592 |
| GNAS | ENSP00000360141 |
| DLX3 | ENSP00000389870 |
| WISP3 | ENSP00000354734 |
| LRP5 | ENSP00000294304 |
| KLK4 | ENSP00000326159 |
| VDR | ENSP00000447173 |
| FGF23 | ENSP00000237837 |
| CLCN7 | ENSP00000372193 |
| COL1A1 | ENSP00000225964 |
| TREM2 | ENSP00000362205 |
| COL9A3 | ENSP00000341640 |
| COMP | ENSP00000222271 |
| LEMD3 | ENSP00000308369 |
| CALCR | ENSP00000352561 |
| ANO5 | ENSP00000315371 |
| ENAM | ENSP00000379383 |
| TNFRSF11A | ENSP00000465500 |
| TCIRG1 | ENSP00000265686 |
| COL9A2 | ENSP00000361834 |
| KL | ENSP00000369442 |
| DSPP | ENSP00000282478 |
| AMELX | ENSP00000370088 |
| COL11A2 | ENSP00000363840 |
| MATN3 | ENSP00000383894 |
| COL1A2 | ENSP00000297268 |
| TYROBP | ENSP00000262629 |
| COL2A1 | ENSP00000369889 |
| SQSTM1 | ENSP00000374455 |
| GALNT3 | ENSP00000376465 |
| SLC26A2 | ENSP00000286298 |

1. Disease genes of cancer

| **Gene symbol** | **Ensembl ID** |
| --- | --- |
| ESR1 | ENSP00000206249 |
| CCND1 | ENSP00000227507 |
| APC | ENSP00000257430 |
| PDGFRB | ENSP00000261799 |
| MSR1 | ENSP00000262101 |
| AXIN1 | ENSP00000262320 |
| EP300 | ENSP00000263253 |
| PIK3CA | ENSP00000263967 |
| TP53 | ENSP00000269305 |
| ERBB2 | ENSP00000269571 |
| AKT1 | ENSP00000270202 |
| EGFR | ENSP00000275493 |
| KIT | ENSP00000288135 |
| ASPSCR1 | ENSP00000302176 |
| AXIN2 | ENSP00000302625 |
| MN1 | ENSP00000304956 |
| TERT | ENSP00000309572 |
| PPP2R1B | ENSP00000311344 |
| MET | ENSP00000317272 |
| PLAG1 | ENSP00000325546 |
| TRIM24 | ENSP00000340507 |
| PTPN11 | ENSP00000340944 |
| RNF6 | ENSP00000342121 |
| CTNNB1 | ENSP00000344456 |
| IGF2R | ENSP00000349437 |
| MAX | ENSP00000351490 |
| TGFBR2 | ENSP00000351905 |
| PTEN | ENSP00000361021 |
| AR | ENSP00000363822 |
| MYC | ENSP00000367207 |
| JAK2 | ENSP00000371067 |
| CDKN2A | ENSP00000394932 |
| NCOA4 | ENSP00000395465 |
| BRCA1 | ENSP00000418960 |
| CEBPA | ENSP00000427514 |
| GPC3 | ENSP00000377836 |
| FLT3 | ENSP00000241453 |
| FLCN | ENSP00000285071 |
| RUNX1 | ENSP00000300305 |
| IRF1 | ENSP00000245414 |
| HNF1A | ENSP00000257555 |
| DCC | ENSP00000389140 |
| EXT1 | ENSP00000367446 |
| PCM1 | ENSP00000327077 |
| MXI1 | ENSP00000331152 |
| PDGFRA | ENSP00000257290 |
| MAP3K8 | ENSP00000263056 |
| TLR2 | ENSP00000260010 |
| BCR | ENSP00000303507 |
| GATA2 | ENSP00000345681 |
| KIF1B | ENSP00000263934 |
| KLF6 | ENSP00000419923 |
| CD82 | ENSP00000227155 |
| PLA2G2A | ENSP00000364252 |
| CDS1 | ENSP00000295887 |
| NSD1 | ENSP00000395929 |
| NTRK1 | ENSP00000431418 |
| MAD1L1 | ENSP00000265854 |
| RET | ENSP00000347942 |
| NF2 | ENSP00000344666 |
| NPM1 | ENSP00000296930 |
| POU6F2 | ENSP00000384004 |
| GATA1 | ENSP00000365858 |
| ETV6 | ENSP00000379658 |
| KRAS | ENSP00000256078 |
| BRAF | ENSP00000288602 |
| HMMR | ENSP00000377492 |
| PDGFB | ENSP00000330382 |
| RASA1 | ENSP00000274376 |
| WWOX | ENSP00000457230 |
| KDR | ENSP00000263923 |
| PAX7 | ENSP00000364524 |
| IL1B | ENSP00000263341 |
| DLC1 | ENSP00000276297 |
| PTCH1 | ENSP00000332353 |
| STK11 | ENSP00000324856 |
| NF1 | ENSP00000351015 |
| FGFR3 | ENSP00000339824 |
| GDNF | ENSP00000409007 |
| CCDC6 | ENSP00000263102 |
| NQO2 | ENSP00000337773 |
| RB1CC1 | ENSP00000025008 |
| PTCH2 | ENSP00000361266 |
| ZFHX3 | ENSP00000268489 |
| HNF1B | ENSP00000225893 |
| BAX | ENSP00000293288 |
| RNASEL | ENSP00000356530 |
| ODC1 | ENSP00000234111 |
| GMPS | ENSP00000419851 |
| NRAS | ENSP00000358548 |
| DLEC1 | ENSP00000308597 |
| FLT4 | ENSP00000261937 |
| PAX3 | ENSP00000375921 |
| RASSF1 | ENSP00000349547 |
| IL1RN | ENSP00000259206 |
| AURKA | ENSP00000216911 |
| NME1 | ENSP00000013034 |
| SMARCE1 | ENSP00000323967 |
| WT1 | ENSP00000331327 |
| HRAS | ENSP00000309845 |
| PHB | ENSP00000300408 |
| LPP | ENSP00000318089 |
| COL4A6 | ENSP00000361290 |
| PRKAR1A | ENSP00000351410 |
| PTPRJ | ENSP00000400010 |
| SMAD4 | ENSP00000341551 |
| IRF4 | ENSP00000370343 |
| MSH2 | ENSP00000233146 |
| SH3GL1 | ENSP00000269886 |
| MCC | ENSP00000386227 |
| PTPN12 | ENSP00000248594 |
| IDH1 | ENSP00000260985 |
| SUFU | ENSP00000358918 |
| PMS2 | ENSP00000265849 |
| RNF139 | ENSP00000304051 |
| PPM1D | ENSP00000306682 |
| TLR4 | ENSP00000363089 |
| CASP8 | ENSP00000351273 |
| RB1 | ENSP00000267163 |
| PICALM | ENSP00000377015 |
| TMEM127 | ENSP00000258439 |
| ANTXR1 | ENSP00000301945 |
| SH2D1A | ENSP00000360181 |
| CDH1 | ENSP00000261769 |
| FH | ENSP00000355518 |
| BRCA2 | ENSP00000369497 |
| CBFB | ENSP00000415151 |
| ATM | ENSP00000278616 |
| PRCC | ENSP00000271526 |
| CYP2A6 | ENSP00000301141 |
| CHEK2 | ENSP00000372023 |
| GOLGA5 | ENSP00000163416 |
| VHL | ENSP00000256474 |
| TRIM33 | ENSP00000351250 |
| DIRC2 | ENSP00000261038 |
| TSG101 | ENSP00000251968 |
| BUB1B | ENSP00000287598 |
| BACH1 | ENSP00000286800 |
| OGG1 | ENSP00000306561 |
| PDGFRL | ENSP00000251630 |
| FEZ1 | ENSP00000278919 |
| MLH3 | ENSP00000348020 |
| XRCC3 | ENSP00000343392 |
| NUP214 | ENSP00000352400 |
| CHIC2 | ENSP00000263921 |
| TACC3 | ENSP00000326550 |
| MINPP1 | ENSP00000361064 |
| MLH1 | ENSP00000231790 |
| PARK2 | ENSP00000355865 |
| ARHGAP26 | ENSP00000274498 |
| SDHB | ENSP00000364649 |
| CASP10 | ENSP00000286186 |
| LIG4 | ENSP00000349393 |
| BLM | ENSP00000347232 |
| BARD1 | ENSP00000260947 |
| SDHD | ENSP00000364699 |
| MSH6 | ENSP00000234420 |
| SDHC | ENSP00000356953 |
| PALB2 | ENSP00000261584 |
| SDHD | ENSP00000456434 |
| FLCN | ENSP00000394249 |
| LZTS1 | ENSP00000265801 |
| ERCC6 | ENSP00000348089 |
| ARHGEF12 | ENSP00000380942 |
| MLF1 | ENSP00000376568 |
| ATR | ENSP00000343741 |
| WHSC1L1 | ENSP00000313983 |
| RAD54L | ENSP00000361043 |
| BRIP1 | ENSP00000259008 |
| MUTYH | ENSP00000361170 |
| HIP1 | ENSP00000336747 |
| RAD54B | ENSP00000336606 |

1. Disease genes of cardiovascular

| **Gene symbol** | **Ensembl ID** |
| --- | --- |
| ESR1 | ENSP00000206249 |
| RETN | ENSP00000221515 |
| GHR | ENSP00000230882 |
| IL6 | ENSP00000258743 |
| PNMT | ENSP00000269582 |
| AGTR1 | ENSP00000273430 |
| PPARG | ENSP00000287820 |
| NEUROD1 | ENSP00000295108 |
| NOS3 | ENSP00000297494 |
| LIPC | ENSP00000299022 |
| LRP8 | ENSP00000303634 |
| IRS1 | ENSP00000304895 |
| OLR1 | ENSP00000309124 |
| HNF4A | ENSP00000312987 |
| KCNJ11 | ENSP00000345708 |
| GIP | ENSP00000350005 |
| ENPP1 | ENSP00000354238 |
| AGT | ENSP00000355627 |
| APOA2 | ENSP00000356969 |
| PTPN1 | ENSP00000360683 |
| ABCA1 | ENSP00000363868 |
| IRS2 | ENSP00000365016 |
| IGF2BP2 | ENSP00000371634 |
| ACVRL1 | ENSP00000373574 |
| ABCC8 | ENSP00000374467 |
| LDLR | ENSP00000454071 |
| ENG | ENSP00000362299 |
| GCK | ENSP00000223366 |
| ABCC9 | ENSP00000261200 |
| ADRB1 | ENSP00000358301 |
| ECE1 | ENSP00000364028 |
| ATP1B1 | ENSP00000356789 |
| SLC2A4 | ENSP00000320935 |
| PAX4 | ENSP00000339906 |
| LTA | ENSP00000403495 |
| HGF | ENSP00000222390 |
| GJA1 | ENSP00000282561 |
| HNF1A | ENSP00000257555 |
| GPD2 | ENSP00000308610 |
| HMGA1 | ENSP00000308227 |
| DMD | ENSP00000354923 |
| LGALS2 | ENSP00000215886 |
| GCGR | ENSP00000383558 |
| TEK | ENSP00000369375 |
| SGCD | ENSP00000338343 |
| PRKAG2 | ENSP00000287878 |
| KCNJ2 | ENSP00000243457 |
| RGS5 | ENSP00000319308 |
| MAPK8IP1 | ENSP00000241014 |
| AKT2 | ENSP00000375892 |
| EYA4 | ENSP00000347294 |
| SLC6A2 | ENSP00000219833 |
| CDKAL1 | ENSP00000274695 |
| PRKCH | ENSP00000329127 |
| SLC30A8 | ENSP00000415011 |
| MTNR1B | ENSP00000257068 |
| GNAI2 | ENSP00000312999 |
| F13A1 | ENSP00000264870 |
| F7 | ENSP00000364731 |
| NOS2 | ENSP00000327251 |
| TBX1 | ENSP00000331791 |
| F5 | ENSP00000356771 |
| RASA1 | ENSP00000274376 |
| GATA6 | ENSP00000269216 |
| ITIH4 | ENSP00000266041 |
| SELE | ENSP00000331736 |
| GCLC | ENSP00000229416 |
| NR3C2 | ENSP00000341390 |
| LMNA | ENSP00000357283 |
| HNF1B | ENSP00000225893 |
| CYP3A5 | ENSP00000222982 |
| F2 | ENSP00000308541 |
| GCLM | ENSP00000359258 |
| PTGIS | ENSP00000244043 |
| WFS1 | ENSP00000226760 |
| TCF7L2 | ENSP00000444972 |
| DTNA | ENSP00000382064 |
| PDE4D | ENSP00000345502 |
| NKX2-6 | ENSP00000320089 |
| EPHX2 | ENSP00000430269 |
| PRKAR1A | ENSP00000351410 |
| CAV3 | ENSP00000341940 |
| NKX2-5 | ENSP00000327758 |
| GNB3 | ENSP00000229264 |
| MEF2A | ENSP00000346389 |
| PKP2 | ENSP00000070846 |
| CRELD1 | ENSP00000321856 |
| KCNQ1 | ENSP00000155840 |
| JAG1 | ENSP00000254958 |
| ADD1 | ENSP00000264758 |
| TNFSF4 | ENSP00000281834 |
| SCN5A | ENSP00000328968 |
| TCF4 | ENSP00000346440 |
| FH | ENSP00000355518 |
| ALOX5AP | ENSP00000369858 |
| GATA4 | ENSP00000334458 |
| DSP | ENSP00000369129 |
| NAT1 | ENSP00000443194 |
| ZFPM2 | ENSP00000384179 |
| KRIT1 | ENSP00000344668 |
| ELN | ENSP00000252034 |
| TNNT2 | ENSP00000356286 |
| NOTCH3 | ENSP00000263388 |
| KCNMB1 | ENSP00000274629 |
| ANK2 | ENSP00000349588 |
| GDF1 | ENSP00000247005 |
| TPM1 | ENSP00000267996 |
| BMPR2 | ENSP00000363708 |
| ITGB3 | ENSP00000456711 |
| CSRP3 | ENSP00000265968 |
| EPHX1 | ENSP00000272167 |
| CFC1 | ENSP00000259216 |
| RYR2 | ENSP00000355533 |
| MYL3 | ENSP00000292327 |
| PSMA6 | ENSP00000261479 |
| MYBPC3 | ENSP00000442795 |
| DES | ENSP00000363071 |
| TCAP | ENSP00000312624 |
| NET1 | ENSP00000347134 |
| TNNI3 | ENSP00000341838 |
| SCO2 | ENSP00000252785 |
| MYLK2 | ENSP00000365152 |
| LIPH | ENSP00000296252 |
| MYL2 | ENSP00000228841 |
| TTN | ENSP00000343764 |
| MYH7 | ENSP00000347507 |

1. Disease genes of connective tissue disorder

| **gene symbol** | **Ensembl ID** |
| --- | --- |
| IL6 | ENSP00000258743 |
| ENPP1 | ENSP00000354238 |
| TGFBR1 | ENSP00000364133 |
| ANKH | ENSP00000284268 |
| SCT | ENSP00000176195 |
| HLA-DRB1 | ENSP00000353099 |
| HGF | ENSP00000222390 |
| COL3A1 | ENSP00000304408 |
| SLC22A4 | ENSP00000200652 |
| GNPAT | ENSP00000355607 |
| B4GALT7 | ENSP00000029410 |
| IL10 | ENSP00000412237 |
| ASPN | ENSP00000364694 |
| COL5A1 | ENSP00000360882 |
| ABCC6 | ENSP00000205557 |
| KRT4 | ENSP00000448220 |
| COL1A1 | ENSP00000225964 |
| SOS1 | ENSP00000384675 |
| EBP | ENSP00000417052 |
| HLA-B | ENSP00000399168 |
| TNXB | ENSP00000407685 |
| ARSE | ENSP00000370526 |
| FBLN5 | ENSP00000345008 |
| FBN1 | ENSP00000325527 |
| CD244 | ENSP00000357012 |
| FGFR2 | ENSP00000410294 |
| PTPN22 | ENSP00000352833 |
| MIF | ENSP00000215754 |
| FRZB | ENSP00000295113 |
| SMARCAL1 | ENSP00000349823 |
| PLOD1 | ENSP00000196061 |
| COL11A2 | ENSP00000363840 |
| COL1A2 | ENSP00000297268 |
| NFKBIL1 | ENSP00000365318 |
| COL5A2 | ENSP00000364000 |
| ADAMTS2 | ENSP00000251582 |
| ELN | ENSP00000252034 |
| COL6A1 | ENSP00000355180 |
| FLNB | ENSP00000420213 |
| ANTXR2 | ENSP00000306185 |
| ADAMTS10 | ENSP00000270328 |
| KRT13 | ENSP00000246635 |
| XYLT2 | ENSP00000017003 |
| XYLT1 | ENSP00000261381 |
| PADI4 | ENSP00000364597 |
| SLC26A2 | ENSP00000286298 |

1. Disease genes of dermatological

| **Gene symbol** | **Ensembl ID** |
| --- | --- |
| TYR | ENSP00000263321 |
| ABCA12 | ENSP00000272895 |
| HR | ENSP00000370826 |
| SOX18 | ENSP00000341815 |
| FLCN | ENSP00000285071 |
| LTA | ENSP00000403495 |
| MBTPS2 | ENSP00000368798 |
| HLA-A | ENSP00000366005 |
| KRT83 | ENSP00000293670 |
| KRT2 | ENSP00000310861 |
| SAT1 | ENSP00000368572 |
| MMP1 | ENSP00000322788 |
| KRT86 | ENSP00000293525 |
| KRT16 | ENSP00000301653 |
| CDSN | ENSP00000365465 |
| SPINK5 | ENSP00000352936 |
| KRT5 | ENSP00000252242 |
| SLURP1 | ENSP00000246515 |
| ZMPSTE24 | ENSP00000361845 |
| CYLD | ENSP00000308928 |
| SLC39A4 | ENSP00000301305 |
| HLA-B | ENSP00000399168 |
| HLA-C | ENSP00000365402 |
| PVRL1 | ENSP00000264025 |
| CDH3 | ENSP00000264012 |
| RAB27A | ENSP00000337761 |
| KRT9 | ENSP00000246662 |
| LMNA | ENSP00000357283 |
| KRT6B | ENSP00000252252 |
| NOD2 | ENSP00000300589 |
| PSTPIP1 | ENSP00000452746 |
| PKP1 | ENSP00000263946 |
| KRT10 | ENSP00000269576 |
| ATP2A2 | ENSP00000440045 |
| KRT6A | ENSP00000369317 |
| KRT17 | ENSP00000308452 |
| ITGA6 | ENSP00000386896 |
| LAMB3 | ENSP00000348384 |
| IKBKG | ENSP00000358622 |
| MYO5A | ENSP00000382177 |
| KRT1 | ENSP00000252244 |
| KRT14 | ENSP00000167586 |
| GJB2 | ENSP00000372295 |
| ITGB4 | ENSP00000200181 |
| ATP2C1 | ENSP00000376914 |
| KRT86 | ENSP00000452237 |
| LAMC2 | ENSP00000264144 |
| ALOX12B | ENSP00000315167 |
| DSP | ENSP00000369129 |
| LAMA3 | ENSP00000324532 |
| COL7A1 | ENSP00000332371 |
| DSG4 | ENSP00000352785 |
| TGM1 | ENSP00000206765 |
| DDB2 | ENSP00000256996 |
| TMC6 | ENSP00000313408 |
| COL17A1 | ENSP00000340937 |
| MPLKIP | ENSP00000304553 |
| KRT81 | ENSP00000369349 |
| EDA | ENSP00000363680 |
| ADAR | ENSP00000357459 |
| GJB4 | ENSP00000345868 |
| FLCN | ENSP00000394249 |
| GJB6 | ENSP00000241124 |
| EDAR | ENSP00000258443 |
| TMC8 | ENSP00000325561 |
| GTF2H5 | ENSP00000415032 |
| GJB3 | ENSP00000362460 |
| MLPH | ENSP00000264605 |
| POLH | ENSP00000361310 |
| ERCC3 | ENSP00000285398 |
| ERCC2 | ENSP00000375809 |
| DSG1 | ENSP00000257192 |
| ERCC5 | ENSP00000347978 |
| DKC1 | ENSP00000358563 |
| ERCC4 | ENSP00000310520 |
| PLEC | ENSP00000323856 |

1. Disease genes of developmental

| **Gene symbol** | **Ensembl ID** |
| --- | --- |
| SIX3 | ENSP00000260653 |
| LHCGR | ENSP00000294954 |
| TSC1 | ENSP00000298552 |
| INSR | ENSP00000303830 |
| PTPN11 | ENSP00000340944 |
| HSD17B3 | ENSP00000364412 |
| CYP1B1 | ENSP00000260630 |
| MKKS | ENSP00000246062 |
| ZEB2 | ENSP00000302501 |
| TWIST1 | ENSP00000242261 |
| PAX6 | ENSP00000368401 |
| MECP2 | ENSP00000395535 |
| NSD1 | ENSP00000395929 |
| SHH | ENSP00000297261 |
| T | ENSP00000296946 |
| CCL2 | ENSP00000225831 |
| TRPS1 | ENSP00000379065 |
| IGF1 | ENSP00000302665 |
| IRF6 | ENSP00000355988 |
| CDKL5 | ENSP00000369325 |
| AMH | ENSP00000221496 |
| ZIC2 | ENSP00000365514 |
| UBE3A | ENSP00000381045 |
| TBX1 | ENSP00000331791 |
| GATA6 | ENSP00000269216 |
| LEMD3 | ENSP00000308369 |
| TBX5 | ENSP00000309913 |
| AMHR2 | ENSP00000257863 |
| NKX2-6 | ENSP00000320089 |
| NSDHL | ENSP00000359297 |
| FUZ | ENSP00000313309 |
| FGFR2 | ENSP00000410294 |
| NKX2-5 | ENSP00000327758 |
| MIF | ENSP00000215754 |
| MTHFR | ENSP00000365775 |
| MED13L | ENSP00000281928 |
| EZH2 | ENSP00000320147 |
| PITX2 | ENSP00000304169 |
| GDF1 | ENSP00000247005 |
| MSX1 | ENSP00000372170 |
| TPM2 | ENSP00000354219 |
| EZH1 | ENSP00000404658 |
| MTRR | ENSP00000264668 |
| TCOF1 | ENSP00000421655 |
| CFC1 | ENSP00000259216 |
| MTR | ENSP00000355536 |
| TNNI2 | ENSP00000252898 |
| TNNT3 | ENSP00000278317 |
| VANGL1 | ENSP00000310800 |
| TBX22 | ENSP00000362390 |
| ATR | ENSP00000343741 |
| NODAL | ENSP00000287139 |
| MYH3 | ENSP00000226209 |
| ESCO2 | ENSP00000306999 |
| NIPBL | ENSP00000282516 |

1. Disease genes of ear nose throat

| **Gene symbol** | **Ensembl ID** |
| --- | --- |
| POU3F4 | ENSP00000362296 |
| KCNJ10 | ENSP00000357068 |
| EYA4 | ENSP00000347294 |
| CTSC | ENSP00000227266 |
| MYO7A | ENSP00000386331 |
| OTOA | ENSP00000373610 |
| WFS1 | ENSP00000226760 |
| MYH14 | ENSP00000262269 |
| CDH23 | ENSP00000381768 |
| KCNQ4 | ENSP00000262916 |
| DSPP | ENSP00000282478 |
| COL11A2 | ENSP00000363840 |
| GJB2 | ENSP00000372295 |
| POU4F3 | ENSP00000230732 |
| DIAPH1 | ENSP00000381565 |
| GRHL2 | ENSP00000251808 |
| USH1C | ENSP00000005226 |
| MYH9 | ENSP00000216181 |
| TAS2R38 | ENSP00000448219 |
| ESPN | ENSP00000367059 |
| FOXI1 | ENSP00000304286 |
| ATP2B2 | ENSP00000324172 |
| TMC1 | ENSP00000297784 |
| TECTA | ENSP00000264037 |
| COCH | ENSP00000216361 |
| PCDH15 | ENSP00000354950 |
| SLC26A4 | ENSP00000265715 |
| GJB6 | ENSP00000241124 |
| DFNB31 | ENSP00000354623 |
| OTOF | ENSP00000272371 |
| TMIE | ENSP00000324775 |
| GJB3 | ENSP00000362460 |
| TMPRSS3 | ENSP00000291532 |
| STRC | ENSP00000401513 |
| MYO1A | ENSP00000300119 |
| MYO15A | ENSP00000205890 |
| MYO6 | ENSP00000358994 |
| MYO3A | ENSP00000265944 |
| ACTG1 | ENSP00000331514 |

1. Disease genes of endocrine

| **Gene symbol** | **Ensembl ID** |
| --- | --- |
| RETN | ENSP00000221515 |
| INS | ENSP00000250971 |
| FSHB | ENSP00000254122 |
| IL6 | ENSP00000258743 |
| PPP1R3A | ENSP00000284601 |
| PPARG | ENSP00000287820 |
| CYP11B1 | ENSP00000292427 |
| STAT5B | ENSP00000293328 |
| NEUROD1 | ENSP00000295108 |
| LIPC | ENSP00000299022 |
| IRS1 | ENSP00000304895 |
| HNF4A | ENSP00000312987 |
| MC2R | ENSP00000333821 |
| AVPR2 | ENSP00000338072 |
| KCNJ11 | ENSP00000345708 |
| ENPP1 | ENSP00000354238 |
| CYP17A1 | ENSP00000358903 |
| PTPN1 | ENSP00000360683 |
| AR | ENSP00000363822 |
| IRS2 | ENSP00000365016 |
| AVP | ENSP00000369647 |
| IGF2BP2 | ENSP00000371634 |
| ABCC8 | ENSP00000374467 |
| CAPN10 | ENSP00000375844 |
| SSTR5 | ENSP00000293897 |
| PAX8 | ENSP00000395498 |
| GCK | ENSP00000223366 |
| TSHR | ENSP00000298171 |
| TBX19 | ENSP00000356795 |
| SLC2A4 | ENSP00000320935 |
| PAX4 | ENSP00000339906 |
| STAR | ENSP00000276449 |
| HGF | ENSP00000222390 |
| BMP15 | ENSP00000252677 |
| HNF1A | ENSP00000257555 |
| GPD2 | ENSP00000308610 |
| HMGA1 | ENSP00000308227 |
| GCGR | ENSP00000383558 |
| GNAS | ENSP00000360141 |
| TPO | ENSP00000318820 |
| TG | ENSP00000220616 |
| THRB | ENSP00000348827 |
| CASR | ENSP00000420194 |
| PROP1 | ENSP00000311290 |
| MAPK8IP1 | ENSP00000241014 |
| ITPR3 | ENSP00000363435 |
| AKT2 | ENSP00000375892 |
| FSHR | ENSP00000384708 |
| CTLA4 | ENSP00000303939 |
| FOXP3 | ENSP00000365380 |
| MRAP | ENSP00000306697 |
| CDKAL1 | ENSP00000274695 |
| FOXE1 | ENSP00000364265 |
| SLC30A8 | ENSP00000415011 |
| MTNR1B | ENSP00000257068 |
| PTH | ENSP00000282091 |
| HESX1 | ENSP00000295934 |
| AQP2 | ENSP00000199280 |
| DHH | ENSP00000266991 |
| NR3C2 | ENSP00000341390 |
| HNF1B | ENSP00000225893 |
| POR | ENSP00000419970 |
| SLC5A5 | ENSP00000222248 |
| AIP | ENSP00000279146 |
| WFS1 | ENSP00000226760 |
| TCF7L2 | ENSP00000444972 |
| PTF1A | ENSP00000365687 |
| GNRHR | ENSP00000226413 |
| SCNN1G | ENSP00000300061 |
| PTPN22 | ENSP00000352833 |
| CACNA1S | ENSP00000355192 |
| SCNN1A | ENSP00000353292 |
| SCNN1B | ENSP00000345751 |
| DUOX2 | ENSP00000373691 |
| TCF4 | ENSP00000346440 |
| GATA3 | ENSP00000368632 |
| OAS1 | ENSP00000388001 |
| STX16 | ENSP00000360183 |
| TBCE | ENSP00000355560 |
| SYCP3 | ENSP00000266743 |
| LIPH | ENSP00000296252 |
| FGD2 | ENSP00000274963 |
| SUMO4 | ENSP00000318635 |
| TTF2 | ENSP00000358478 |

1. Disease genes of gastrointestinal

| **Gene symbol** | **Ensembl ID** |
| --- | --- |
| IL6 | ENSP00000258743 |
| ABCB4 | ENSP00000265723 |
| HGF | ENSP00000222390 |
| HSD3B7 | ENSP00000297679 |
| EDNRB | ENSP00000366416 |
| ABCB11 | ENSP00000263817 |
| RET | ENSP00000347942 |
| SAR1B | ENSP00000385432 |
| BAAT | ENSP00000259407 |
| SLC26A3 | ENSP00000345873 |
| FGF10 | ENSP00000264664 |
| UGT1A1 | ENSP00000304845 |
| CFTR | ENSP00000003084 |
| ATP8B1 | ENSP00000283684 |
| NOD2 | ENSP00000300589 |
| TJP2 | ENSP00000438262 |
| SEC63 | ENSP00000357998 |
| PRKCSH | ENSP00000252455 |
| CTRC | ENSP00000365116 |
| SPINK1 | ENSP00000296695 |
| KRT8 | ENSP00000293308 |
| KRT18 | ENSP00000373487 |
| EPHX1 | ENSP00000272167 |
| PRSS1 | ENSP00000308720 |
| VPS33B | ENSP00000327650 |
| CIRH1A | ENSP00000327179 |

1. Disease genes of hematological

| **Gene symbol** | **Ensembl ID** |
| --- | --- |
| CFHR1 | ENSP00000314299 |
| CR1 | ENSP00000356016 |
| CFHR3 | ENSP00000356395 |
| CFH | ENSP00000356399 |
| JAK2 | ENSP00000371067 |
| A4GALT | ENSP00000249005 |
| THPO | ENSP00000204615 |
| CD36 | ENSP00000308165 |
| APOE | ENSP00000252486 |
| AMN | ENSP00000299155 |
| RUNX1 | ENSP00000300305 |
| IFNG | ENSP00000229135 |
| CPN1 | ENSP00000359446 |
| FOXC2 | ENSP00000326371 |
| PDGFRA | ENSP00000257290 |
| MPL | ENSP00000361548 |
| FGA | ENSP00000306361 |
| TPO | ENSP00000318820 |
| GSS | ENSP00000216951 |
| EPOR | ENSP00000222139 |
| CUBN | ENSP00000367064 |
| GIF | ENSP00000257248 |
| AQP3 | ENSP00000297991 |
| ANKRD26 | ENSP00000365255 |
| AQP1 | ENSP00000421315 |
| GATA1 | ENSP00000365858 |
| GGCX | ENSP00000233838 |
| ELANE | ENSP00000263621 |
| SLC19A2 | ENSP00000236137 |
| SH2B3 | ENSP00000345492 |
| AQP1 | ENSP00000311165 |
| GP9 | ENSP00000303942 |
| TF | ENSP00000385834 |
| GCLC | ENSP00000229416 |
| GP1BB | ENSP00000383382 |
| SLC4A1 | ENSP00000262418 |
| BSG | ENSP00000333769 |
| UNC13D | ENSP00000207549 |
| FLT4 | ENSP00000261937 |
| GP1BA | ENSP00000329380 |
| RHAG | ENSP00000360217 |
| ITGA2B | ENSP00000262407 |
| ADAMTS13 | ENSP00000360997 |
| ACHE | ENSP00000303211 |
| VKORC1 | ENSP00000431371 |
| NT5C3A | ENSP00000242210 |
| VKORC1 | ENSP00000378426 |
| CP | ENSP00000264613 |
| LBR | ENSP00000272163 |
| HBB | ENSP00000333994 |
| ATRX | ENSP00000362441 |
| CYP2A6 | ENSP00000301141 |
| MYH9 | ENSP00000216181 |
| VHL | ENSP00000256474 |
| F9 | ENSP00000218099 |
| EPX | ENSP00000225371 |
| CDAN1 | ENSP00000348564 |
| FGB | ENSP00000306099 |
| WAS | ENSP00000365891 |
| ITGB3 | ENSP00000456711 |
| MASTL | ENSP00000365107 |
| GCNT2 | ENSP00000265012 |
| CYP2C9 | ENSP00000260682 |
| ABCB7 | ENSP00000253577 |
| FHL3 | ENSP00000362107 |
| LMAN1 | ENSP00000251047 |
| RPS14 | ENSP00000311028 |
| RPS19 | ENSP00000470004 |

1. Disease genes of immunological

| **Gene symbol** | **Ensembl ID** |
| --- | --- |
| CEBPE | ENSP00000206513 |
| MC3R | ENSP00000243911 |
| IL6 | ENSP00000258743 |
| ITGB2 | ENSP00000303242 |
| STAT1 | ENSP00000354394 |
| JAK3 | ENSP00000391676 |
| CISH | ENSP00000409346 |
| ICOS | ENSP00000319476 |
| HLA-DRB1 | ENSP00000353099 |
| C4A | ENSP00000396688 |
| IFNG | ENSP00000229135 |
| HGF | ENSP00000222390 |
| TLR5 | ENSP00000340089 |
| TLR2 | ENSP00000260010 |
| SLC11A1 | ENSP00000233202 |
| MS4A2 | ENSP00000278888 |
| IL10 | ENSP00000412237 |
| SELP | ENSP00000263686 |
| IL21R | ENSP00000338010 |
| CCL3 | ENSP00000225245 |
| NCF1 | ENSP00000289473 |
| IFNGR2 | ENSP00000290219 |
| CCL2 | ENSP00000225831 |
| IFNGR1 | ENSP00000356713 |
| FCGR2B | ENSP00000351497 |
| HAVCR1 | ENSP00000344844 |
| PLA2G7 | ENSP00000274793 |
| IL12RB1 | ENSP00000403103 |
| TAP1 | ENSP00000346206 |
| KIR3DS1 | ENSP00000383672 |
| TNFRSF13B | ENSP00000261652 |
| CXCR4 | ENSP00000386884 |
| SPINK5 | ENSP00000352936 |
| IL4R | ENSP00000170630 |
| IL12B | ENSP00000231228 |
| TNFRSF1A | ENSP00000162749 |
| AIRE | ENSP00000291582 |
| CCL11 | ENSP00000302234 |
| HLA-C | ENSP00000365402 |
| CYBB | ENSP00000367851 |
| DNASE1 | ENSP00000246949 |
| BTK | ENSP00000308176 |
| CD209 | ENSP00000315477 |
| IL2RG | ENSP00000363318 |
| SERPING1 | ENSP00000278407 |
| IL13 | ENSP00000304915 |
| KIR3DL1 | ENSP00000375608 |
| CD40 | ENSP00000361359 |
| CD40LG | ENSP00000359663 |
| NCF2 | ENSP00000356505 |
| CYBA | ENSP00000261623 |
| IRGM | ENSP00000428220 |
| PTPN22 | ENSP00000352833 |
| CASP8 | ENSP00000351273 |
| FAS | ENSP00000347979 |
| CXCL12 | ENSP00000379140 |
| TIRAP | ENSP00000376445 |
| TAP2 | ENSP00000391806 |
| CCL3L1 | ENSP00000408984 |
| TLR3 | ENSP00000296795 |
| ATM | ENSP00000278616 |
| MVK | ENSP00000228510 |
| PTPRZ1 | ENSP00000377047 |
| TAP2 | ENSP00000364034 |
| MRE11A | ENSP00000325863 |
| NLRP3 | ENSP00000337383 |
| CD8A | ENSP00000283635 |
| CXCR1 | ENSP00000295683 |
| ADA | ENSP00000361965 |
| RAG1 | ENSP00000299440 |
| TAPBP | ENSP00000404833 |
| MEFV | ENSP00000219596 |
| WAS | ENSP00000365891 |
| RAG2 | ENSP00000308620 |
| SP110 | ENSP00000258381 |
| RFX5 | ENSP00000290524 |
| CX3CR1 | ENSP00000351059 |
| RAC2 | ENSP00000249071 |
| IRAK4 | ENSP00000390651 |
| PARK2 | ENSP00000355865 |
| RFXANK | ENSP00000305071 |
| CASP10 | ENSP00000286186 |
| LIG4 | ENSP00000349393 |
| AICDA | ENSP00000229335 |
| UNG | ENSP00000242576 |
| BANK1 | ENSP00000320509 |
| MPO | ENSP00000225275 |
| RFXAP | ENSP00000255476 |
| PHF11 | ENSP00000367570 |
| DCLRE1C | ENSP00000367527 |
| TREX1 | ENSP00000390478 |

1. Disease genes of metabolic

| **Gene symbol** | **Ensembl ID** |
| --- | --- |
| CETP | ENSP00000200676 |
| APOA5 | ENSP00000227665 |
| GHR | ENSP00000230882 |
| APOB | ENSP00000233242 |
| APOA1 | ENSP00000236850 |
| CYP27A1 | ENSP00000258415 |
| MLYCD | ENSP00000262430 |
| LCAT | ENSP00000264005 |
| SLC5A1 | ENSP00000266088 |
| ABCG8 | ENSP00000272286 |
| PPP1R3A | ENSP00000284601 |
| PPARG | ENSP00000287820 |
| PCSK9 | ENSP00000303208 |
| MPI | ENSP00000318318 |
| TALDO1 | ENSP00000321259 |
| HLCS | ENSP00000338387 |
| DDC | ENSP00000350616 |
| APOA2 | ENSP00000356969 |
| LDLRAP1 | ENSP00000363458 |
| ABCA1 | ENSP00000363868 |
| ALPL | ENSP00000363965 |
| H6PD | ENSP00000366620 |
| GK | ENSP00000368226 |
| PDX1 | ENSP00000370421 |
| ABCC8 | ENSP00000374467 |
| DBH | ENSP00000376776 |
| NAGA | ENSP00000379680 |
| LDLR | ENSP00000454071 |
| APOC2 | ENSP00000466775 |
| RPIA | ENSP00000283646 |
| GCK | ENSP00000223366 |
| CPT1A | ENSP00000265641 |
| CPT2 | ENSP00000360541 |
| FMO3 | ENSP00000356729 |
| SCD | ENSP00000359380 |
| GNS | ENSP00000258145 |
| D2HGDH | ENSP00000315351 |
| CYP11B2 | ENSP00000325822 |
| SLC17A5 | ENSP00000348019 |
| SLC2A1 | ENSP00000416293 |
| AMN | ENSP00000299155 |
| BSCL2 | ENSP00000354032 |
| AGPAT2 | ENSP00000360761 |
| HAL | ENSP00000261208 |
| USF1 | ENSP00000356999 |
| SLC2A2 | ENSP00000323568 |
| PHEX | ENSP00000368682 |
| LIPI | ENSP00000343331 |
| ABCG5 | ENSP00000260645 |
| SLC22A5 | ENSP00000245407 |
| MANBA | ENSP00000226578 |
| GBE1 | ENSP00000410833 |
| MCCC2 | ENSP00000343657 |
| PYGM | ENSP00000164139 |
| IDUA | ENSP00000247933 |
| PPOX | ENSP00000343943 |
| GSS | ENSP00000216951 |
| MAN2B1 | ENSP00000395473 |
| L2HGDH | ENSP00000267436 |
| NPC2 | ENSP00000451112 |
| GALT | ENSP00000451792 |
| GLUD1 | ENSP00000277865 |
| ABHD5 | ENSP00000390849 |
| HSD17B4 | ENSP00000420914 |
| HGD | ENSP00000283871 |
| ALG2 | ENSP00000417764 |
| HMGCS2 | ENSP00000358414 |
| MGAT2 | ENSP00000307423 |
| NPC1 | ENSP00000269228 |
| ACAT1 | ENSP00000265838 |
| ECM1 | ENSP00000358045 |
| MMAB | ENSP00000445920 |
| DHCR24 | ENSP00000360316 |
| HEXB | ENSP00000261416 |
| ACADVL | ENSP00000349297 |
| HEXA | ENSP00000268097 |
| ALDH4A1 | ENSP00000290597 |
| HYAL1 | ENSP00000266031 |
| GRHPR | ENSP00000313432 |
| PHKB | ENSP00000313504 |
| MMAA | ENSP00000281317 |
| SLC7A7 | ENSP00000285850 |
| GYS2 | ENSP00000261195 |
| GNMT | ENSP00000361894 |
| ALG9 | ENSP00000456645 |
| ITIH4 | ENSP00000266041 |
| UGT1A1 | ENSP00000304845 |
| SLC6A19 | ENSP00000305302 |
| CPS1 | ENSP00000402608 |
| ARG1 | ENSP00000357066 |
| PRODH | ENSP00000349577 |
| MCOLN1 | ENSP00000264079 |
| GALK1 | ENSP00000225614 |
| AUH | ENSP00000364883 |
| LMNA | ENSP00000357283 |
| ATIC | ENSP00000236959 |
| ALG8 | ENSP00000299626 |
| ACADM | ENSP00000409612 |
| BCKDHA | ENSP00000443246 |
| MCCC1 | ENSP00000265594 |
| BTD | ENSP00000306477 |
| ALDH3A2 | ENSP00000345774 |
| ALG6 | ENSP00000360149 |
| MUT | ENSP00000274813 |
| GNPTAB | ENSP00000299314 |
| NEU1 | ENSP00000364782 |
| WFS1 | ENSP00000226760 |
| BCKDHB | ENSP00000318351 |
| PCCB | ENSP00000419027 |
| PANK2 | ENSP00000313377 |
| GBA | ENSP00000314508 |
| ADSL | ENSP00000216194 |
| ASS1 | ENSP00000253004 |
| EPHX2 | ENSP00000430269 |
| PCCA | ENSP00000365462 |
| NAGS | ENSP00000293404 |
| CAV3 | ENSP00000341940 |
| IVD | ENSP00000418397 |
| UROS | ENSP00000357775 |
| BCKDHA | ENSP00000269980 |
| GALT | ENSP00000368119 |
| ETFB | ENSP00000346173 |
| GNE | ENSP00000379839 |
| ABCC2 | ENSP00000359478 |
| MADD | ENSP00000310933 |
| FTCD | ENSP00000291670 |
| XDH | ENSP00000368727 |
| ETFA | ENSP00000452762 |
| DPM1 | ENSP00000360644 |
| ALG1 | ENSP00000262374 |
| GCDH | ENSP00000222214 |
| ETFDH | ENSP00000426638 |
| MTHFR | ENSP00000365775 |
| SLC35C1 | ENSP00000313318 |
| ACADS | ENSP00000242592 |
| DPAGT1 | ENSP00000346142 |
| ASPA | ENSP00000263080 |
| GAA | ENSP00000305692 |
| PC | ENSP00000377527 |
| CP | ENSP00000264613 |
| HSD17B10 | ENSP00000168216 |
| FH | ENSP00000355518 |
| TFR2 | ENSP00000223051 |
| HFE | ENSP00000417404 |
| MPDU1 | ENSP00000250124 |
| GLDC | ENSP00000370737 |
| ETHE1 | ENSP00000292147 |
| DBT | ENSP00000359151 |
| SMPD1 | ENSP00000340409 |
| GALE | ENSP00000363621 |
| COG7 | ENSP00000305442 |
| CTH | ENSP00000359976 |
| SLC40A1 | ENSP00000261024 |
| ALG3 | ENSP00000380793 |
| MCM6 | ENSP00000264156 |
| ATP7B | ENSP00000242839 |
| AGXT | ENSP00000302620 |
| MTRR | ENSP00000264668 |
| ABCD1 | ENSP00000218104 |
| LAMP2 | ENSP00000408411 |
| OTC | ENSP00000039007 |
| GCSH | ENSP00000319531 |
| MTR | ENSP00000355536 |
| PRODH2 | ENSP00000301175 |
| PHGDH | ENSP00000358417 |
| SUOX | ENSP00000266971 |
| SLC25A13 | ENSP00000400101 |
| AASS | ENSP00000377040 |
| SUMF1 | ENSP00000272902 |
| ALG12 | ENSP00000333813 |
| HPRT1 | ENSP00000298556 |
| BCS1L | ENSP00000352219 |
| SGSH | ENSP00000314606 |
| PMM2 | ENSP00000268261 |
| AMT | ENSP00000273588 |
| DMGDH | ENSP00000255189 |
| OCRL | ENSP00000360154 |
| MOCS1 | ENSP00000362282 |
| ASL | ENSP00000307188 |
| OPA3 | ENSP00000319817 |
| ALG9 | ENSP00000435517 |
| RP1 | ENSP00000220676 |
| B4GALT1 | ENSP00000369055 |
| SLC25A15 | ENSP00000342267 |

1. Disease genes of multiple

| **Gene symbol** | **Ensembl ID** |
| --- | --- |
| BBS9 | ENSP00000242067 |
| BBS2 | ENSP00000245157 |
| CREBBP | ENSP00000262367 |
| TYR | ENSP00000263321 |
| LIFR | ENSP00000263409 |
| BBS7 | ENSP00000264499 |
| BBS4 | ENSP00000268057 |
| NDUFAF2 | ENSP00000296597 |
| TSC1 | ENSP00000298552 |
| INSR | ENSP00000303830 |
| BBS12 | ENSP00000319062 |
| ARL6 | ENSP00000337722 |
| PTPN11 | ENSP00000340944 |
| VPS13B | ENSP00000351346 |
| PTEN | ENSP00000361021 |
| CCDC28B | ENSP00000362704 |
| GRIP1 | ENSP00000381098 |
| PAX2 | ENSP00000396259 |
| BBS1 | ENSP00000398526 |
| GPC3 | ENSP00000377836 |
| BBS5 | ENSP00000295240 |
| DHCR7 | ENSP00000347717 |
| TTC8 | ENSP00000370031 |
| BBS5 | ENSP00000424363 |
| FOXL2 | ENSP00000333188 |
| CRLF1 | ENSP00000376188 |
| BBS1 | ENSP00000317469 |
| SALL4 | ENSP00000217086 |
| JUP | ENSP00000311113 |
| MKKS | ENSP00000246062 |
| BBS10 | ENSP00000376946 |
| CHD7 | ENSP00000392028 |
| EDNRB | ENSP00000366416 |
| TFAP2B | ENSP00000377265 |
| CEP290 | ENSP00000448012 |
| HSPG2 | ENSP00000363827 |
| RPS6KA3 | ENSP00000368884 |
| SHANK3 | ENSP00000442518 |
| MITF | ENSP00000295600 |
| GNAS | ENSP00000360141 |
| DLX3 | ENSP00000389870 |
| NSD1 | ENSP00000395929 |
| FOXP3 | ENSP00000365380 |
| SNAI2 | ENSP00000020945 |
| IRF6 | ENSP00000355988 |
| GATA1 | ENSP00000365858 |
| FREM2 | ENSP00000280481 |
| GLI3 | ENSP00000379258 |
| TBX1 | ENSP00000331791 |
| EGR2 | ENSP00000242480 |
| FLNA | ENSP00000358866 |
| RASA1 | ENSP00000274376 |
| HESX1 | ENSP00000295934 |
| NDN | ENSP00000332643 |
| ZMPSTE24 | ENSP00000361845 |
| CTSC | ENSP00000227266 |
| LEMD3 | ENSP00000308369 |
| PTCH1 | ENSP00000332353 |
| SDS | ENSP00000257549 |
| SALL1 | ENSP00000251020 |
| TP63 | ENSP00000264731 |
| UGT1A1 | ENSP00000304845 |
| MKS1 | ENSP00000376827 |
| CDKN1C | ENSP00000411552 |
| FRAS1 | ENSP00000264895 |
| AHI1 | ENSP00000265602 |
| LMX1B | ENSP00000347684 |
| NUBPL | ENSP00000281081 |
| NOD2 | ENSP00000300589 |
| WRN | ENSP00000298139 |
| TRIM37 | ENSP00000262294 |
| SLC12A1 | ENSP00000370381 |
| TRIM32 | ENSP00000363095 |
| PAX3 | ENSP00000375921 |
| GFAP | ENSP00000253408 |
| WNT3 | ENSP00000225512 |
| WT1 | ENSP00000331327 |
| PEX1 | ENSP00000248633 |
| HRAS | ENSP00000309845 |
| MYCN | ENSP00000281043 |
| FGFR1 | ENSP00000393312 |
| BSND | ENSP00000360312 |
| TMEM67 | ENSP00000389998 |
| TAZ | ENSP00000299328 |
| MGP | ENSP00000228938 |
| COL18A1 | ENSP00000347665 |
| PRKAR1A | ENSP00000351410 |
| FGFR2 | ENSP00000410294 |
| CDH23 | ENSP00000381768 |
| SMS | ENSP00000385746 |
| NOG | ENSP00000328181 |
| SKI | ENSP00000367797 |
| LYST | ENSP00000374443 |
| KCNQ1 | ENSP00000155840 |
| JAG1 | ENSP00000254958 |
| DYM | ENSP00000269445 |
| KCNJ1 | ENSP00000376432 |
| ROR2 | ENSP00000364860 |
| COL11A2 | ENSP00000363840 |
| GJB2 | ENSP00000372295 |
| MPZ | ENSP00000431538 |
| AAAS | ENSP00000209873 |
| FANCA | ENSP00000373952 |
| CACNA1C | ENSP00000266376 |
| SEMA3E | ENSP00000303212 |
| CLRN1 | ENSP00000329158 |
| BRCA2 | ENSP00000369497 |
| DSP | ENSP00000369129 |
| CLDN1 | ENSP00000295522 |
| ATP7A | ENSP00000345728 |
| GPR98 | ENSP00000384582 |
| LAMA3 | ENSP00000324532 |
| USH1C | ENSP00000005226 |
| ATRX | ENSP00000362441 |
| PHF6 | ENSP00000329097 |
| FOXRED1 | ENSP00000263578 |
| NLRP3 | ENSP00000337383 |
| DNMT3B | ENSP00000328547 |
| PITX2 | ENSP00000304169 |
| MYH9 | ENSP00000216181 |
| CLCNKB | ENSP00000364831 |
| COL11A1 | ENSP00000359114 |
| TBX3 | ENSP00000257566 |
| PMP22 | ENSP00000308937 |
| ARX | ENSP00000368332 |
| MYH8 | ENSP00000384330 |
| FANCB | ENSP00000326819 |
| CUL7 | ENSP00000438788 |
| COL2A1 | ENSP00000369889 |
| PRX | ENSP00000326018 |
| USH1G | ENSP00000320076 |
| BACH1 | ENSP00000286800 |
| MID1 | ENSP00000312678 |
| AGPS | ENSP00000264167 |
| PDZD7 | ENSP00000359234 |
| HOXA11 | ENSP00000006015 |
| AP3B1 | ENSP00000255194 |
| MYOC | ENSP00000037502 |
| CFC1 | ENSP00000259216 |
| USH2A | ENSP00000305941 |
| BCOR | ENSP00000367705 |
| NDUFS1 | ENSP00000392709 |
| NDUFV1 | ENSP00000322450 |
| NDUFAF5 | ENSP00000367346 |
| TBCE | ENSP00000355560 |
| POMT1 | ENSP00000361302 |
| ERCC8 | ENSP00000265038 |
| LIG4 | ENSP00000349393 |
| PCDH15 | ENSP00000354950 |
| ZIC3 | ENSP00000287538 |
| KIAA1279 | ENSP00000354848 |
| RAI1 | ENSP00000323074 |
| PEX7 | ENSP00000315680 |
| NDUFA1 | ENSP00000360492 |
| SDHA | ENSP00000264932 |
| SBDS | ENSP00000246868 |
| BCS1L | ENSP00000352219 |
| HOXA13 | ENSP00000222753 |
| NDUFAF3 | ENSP00000323076 |
| INPP5E | ENSP00000360777 |
| RAB3GAP1 | ENSP00000411418 |
| SDHAF1 | ENSP00000368165 |
| SNRPN | ENSP00000306223 |
| TYMP | ENSP00000252029 |
| ERCC6 | ENSP00000348089 |
| LOR | ENSP00000357731 |
| HPS1 | ENSP00000326649 |
| NDUFB9 | ENSP00000276689 |
| GFM1 | ENSP00000419038 |
| DGUOK | ENSP00000264093 |
| L1CAM | ENSP00000359074 |
| ATPAF2 | ENSP00000417190 |
| DNAI1 | ENSP00000242317 |
| NDUFS4 | ENSP00000296684 |
| PUS1 | ENSP00000365837 |
| POMGNT1 | ENSP00000361052 |
| KIF7 | ENSP00000377934 |
| NDUFS2 | ENSP00000356972 |
| NDUFA11 | ENSP00000389160 |
| NDUFAF1 | ENSP00000260361 |
| NDUFS6 | ENSP00000274137 |
| BRIP1 | ENSP00000259008 |
| NDUFB3 | ENSP00000237889 |
| NDUFAF4 | ENSP00000358272 |
| NDUFV2 | ENSP00000327268 |
| CHM | ENSP00000350386 |
| NDUFS3 | ENSP00000263774 |

1. Disease genes of muscular

| **Gene symbol** | **Ensembl ID** |
| --- | --- |
| AR | ENSP00000363822 |
| CPT2 | ENSP00000360541 |
| EMD | ENSP00000358857 |
| BSCL2 | ENSP00000354032 |
| MYOT | ENSP00000239926 |
| CHRND | ENSP00000258385 |
| DYSF | ENSP00000386881 |
| DMD | ENSP00000354923 |
| SCG3 | ENSP00000220478 |
| ATP2A1 | ENSP00000349595 |
| SGCD | ENSP00000338343 |
| SGCB | ENSP00000370839 |
| CRYAB | ENSP00000227251 |
| SGCA | ENSP00000262018 |
| SGCG | ENSP00000218867 |
| LMNA | ENSP00000357283 |
| RYR1 | ENSP00000352608 |
| TRIM32 | ENSP00000363095 |
| CHRNA1 | ENSP00000261007 |
| FKRP | ENSP00000326570 |
| CHAT | ENSP00000337103 |
| MTM1 | ENSP00000359423 |
| SMN2 | ENSP00000370119 |
| CAV3 | ENSP00000341940 |
| GNE | ENSP00000379839 |
| IGHMBP2 | ENSP00000255078 |
| SMN1 | ENSP00000370083 |
| SEPN1 | ENSP00000355141 |
| MUSK | ENSP00000363571 |
| MYH2 | ENSP00000245503 |
| DNM2 | ENSP00000347890 |
| SMAD1 | ENSP00000305769 |
| VCP | ENSP00000351777 |
| GARS | ENSP00000373918 |
| COL6A2 | ENSP00000300527 |
| TNNT1 | ENSP00000467176 |
| CAPN3 | ENSP00000380349 |
| DMPK | ENSP00000345997 |
| COL6A1 | ENSP00000355180 |
| CLCN1 | ENSP00000339867 |
| COL6A3 | ENSP00000295550 |
| SCN4A | ENSP00000396320 |
| ACTA1 | ENSP00000355645 |
| CHRNB1 | ENSP00000304290 |
| POMT1 | ENSP00000361302 |
| NEB | ENSP00000380505 |
| MTMR14 | ENSP00000296003 |
| DES | ENSP00000363071 |
| LARGE | ENSP00000347088 |
| TCAP | ENSP00000312624 |
| RAPSN | ENSP00000298854 |
| FKTN | ENSP00000223528 |
| VAPB | ENSP00000417175 |
| CHRNE | ENSP00000293780 |
| DGUOK | ENSP00000264093 |
| LAMA2 | ENSP00000400365 |
| TTN | ENSP00000343764 |
| PABPN1 | ENSP00000216727 |
| MYH7 | ENSP00000347507 |
| PLEC | ENSP00000323856 |

1. Disease genes of neurological

| **Gene symbol** | **Ensembl ID** |
| --- | --- |
| ESR1 | ENSP00000206249 |
| APOA1 | ENSP00000236850 |
| SIX3 | ENSP00000260653 |
| ACE | ENSP00000290866 |
| NDUFAF2 | ENSP00000296597 |
| NOS3 | ENSP00000297494 |
| ATP1A3 | ENSP00000302397 |
| SNCA | ENSP00000338345 |
| MAPT | ENSP00000340820 |
| ATP1A2 | ENSP00000354490 |
| DRD2 | ENSP00000354859 |
| GJC2 | ENSP00000355675 |
| SOX3 | ENSP00000359567 |
| PLAU | ENSP00000361850 |
| PDCD10 | ENSP00000376506 |
| RELN | ENSP00000392423 |
| ACE | ENSP00000464149 |
| NHLRC1 | ENSP00000345464 |
| VLDLR | ENSP00000371532 |
| FGF14 | ENSP00000365301 |
| NDRG1 | ENSP00000319977 |
| SLC6A8 | ENSP00000253122 |
| MLC1 | ENSP00000310375 |
| SLC12A6 | ENSP00000452776 |
| PRPH | ENSP00000257860 |
| ACSL4 | ENSP00000339787 |
| VPS13A | ENSP00000353422 |
| SERPINI1 | ENSP00000295777 |
| EPM2A | ENSP00000356489 |
| NDUFAF6 | ENSP00000379430 |
| TNF | ENSP00000398698 |
| APP | ENSP00000284981 |
| APOE | ENSP00000252486 |
| SORL1 | ENSP00000260197 |
| SYN1 | ENSP00000295987 |
| AMN | ENSP00000299155 |
| HLA-DRB1 | ENSP00000353099 |
| BSCL2 | ENSP00000354032 |
| LRRK2 | ENSP00000298910 |
| THBS2 | ENSP00000355751 |
| NKX2-1 | ENSP00000346879 |
| CILP | ENSP00000261883 |
| LGI1 | ENSP00000360472 |
| GSN | ENSP00000362924 |
| PDCD1 | ENSP00000335062 |
| SOD1 | ENSP00000270142 |
| NEFH | ENSP00000311997 |
| HSPB1 | ENSP00000248553 |
| SLC16A2 | ENSP00000276033 |
| EDNRA | ENSP00000315011 |
| HSPG2 | ENSP00000363827 |
| SCN1A | ENSP00000303540 |
| FGA | ENSP00000306361 |
| KIF1B | ENSP00000263934 |
| GCH1 | ENSP00000378890 |
| HLA-DQB1 | ENSP00000382034 |
| SLC19A3 | ENSP00000258403 |
| SGCE | ENSP00000398930 |
| ASPN | ENSP00000364694 |
| MECP2 | ENSP00000395535 |
| HOXA1 | ENSP00000343246 |
| GJB1 | ENSP00000354900 |
| PLP1 | ENSP00000305152 |
| SHH | ENSP00000297261 |
| NTRK1 | ENSP00000431418 |
| DCX | ENSP00000337697 |
| PNKD | ENSP00000273077 |
| RET | ENSP00000347942 |
| PSEN1 | ENSP00000326366 |
| CSTB | ENSP00000291568 |
| LITAF | ENSP00000340118 |
| KCNA1 | ENSP00000371985 |
| ARFGEF2 | ENSP00000360985 |
| EYA1 | ENSP00000342626 |
| ATXN1 | ENSP00000244769 |
| CST3 | ENSP00000366124 |
| CCM2 | ENSP00000370503 |
| FOXP2 | ENSP00000386200 |
| PER2 | ENSP00000254657 |
| SPG20 | ENSP00000347314 |
| EGR2 | ENSP00000242480 |
| FLNA | ENSP00000358866 |
| KCNMA1 | ENSP00000385806 |
| LYZ | ENSP00000261267 |
| COL9A3 | ENSP00000341640 |
| LRPPRC | ENSP00000260665 |
| CHRNA4 | ENSP00000359285 |
| ATXN3 | ENSP00000376965 |
| PRNP | ENSP00000368752 |
| SNCB | ENSP00000308057 |
| PPP2R2B | ENSP00000336591 |
| PARK7 | ENSP00000340278 |
| TOR1A | ENSP00000345719 |
| APTX | ENSP00000369141 |
| GABRG2 | ENSP00000410732 |
| ITM2B | ENSP00000367828 |
| NIPA1 | ENSP00000337452 |
| SCN9A | ENSP00000386306 |
| ALMS1 | ENSP00000264448 |
| MFN2 | ENSP00000235329 |
| ATL1 | ENSP00000351155 |
| PEX5 | ENSP00000407401 |
| TTPA | ENSP00000260116 |
| SOX10 | ENSP00000354130 |
| PRKCG | ENSP00000263431 |
| SPAST | ENSP00000320885 |
| TPP1 | ENSP00000299427 |
| RYR1 | ENSP00000352608 |
| CACNA1A | ENSP00000353362 |
| PINK1 | ENSP00000364204 |
| TDP1 | ENSP00000337353 |
| ME2 | ENSP00000321070 |
| FTL | ENSP00000366525 |
| PANK2 | ENSP00000313377 |
| GBA | ENSP00000314508 |
| NLGN3 | ENSP00000351591 |
| WNK1 | ENSP00000313059 |
| COL9A2 | ENSP00000361834 |
| HSPD1 | ENSP00000340019 |
| EIF2B5 | ENSP00000273783 |
| SMS | ENSP00000385746 |
| PSEN2 | ENSP00000355747 |
| KIF5A | ENSP00000408979 |
| SCN2A | ENSP00000283256 |
| PDHA1 | ENSP00000369134 |
| CACNA1S | ENSP00000355192 |
| DNM2 | ENSP00000347890 |
| CLN6 | ENSP00000249806 |
| CLN3 | ENSP00000353073 |
| SH3TC2 | ENSP00000423660 |
| GLRA1 | ENSP00000411593 |
| ATXN7 | ENSP00000381590 |
| HSPB8 | ENSP00000281938 |
| ZNF81 | ENSP00000341151 |
| MPZ | ENSP00000431538 |
| SMAD1 | ENSP00000305769 |
| KCNQ3 | ENSP00000373648 |
| CP | ENSP00000264613 |
| ARHGEF10 | ENSP00000340297 |
| COLQ | ENSP00000373298 |
| SBF2 | ENSP00000256190 |
| CLCN2 | ENSP00000265593 |
| GARS | ENSP00000373918 |
| SLC25A22 | ENSP00000322020 |
| APBB2 | ENSP00000427211 |
| MTMR2 | ENSP00000345752 |
| GAN | ENSP00000248272 |
| ARSA | ENSP00000216124 |
| KCNQ2 | ENSP00000352035 |
| PHYH | ENSP00000263038 |
| HFE | ENSP00000417404 |
| CACNB4 | ENSP00000438949 |
| PQBP1 | ENSP00000218224 |
| COL4A1 | ENSP00000364979 |
| ATP7A | ENSP00000345728 |
| PAFAH1B1 | ENSP00000380378 |
| GPR98 | ENSP00000384582 |
| PDC | ENSP00000342033 |
| GDAP1 | ENSP00000220822 |
| KRIT1 | ENSP00000344668 |
| FOXRED1 | ENSP00000263578 |
| KDM5C | ENSP00000364550 |
| COL11A1 | ENSP00000359114 |
| CENPJ | ENSP00000371308 |
| SLC25A19 | ENSP00000319574 |
| DCTN1 | ENSP00000354791 |
| EIF2B1 | ENSP00000416250 |
| CHRNB2 | ENSP00000357461 |
| PMP22 | ENSP00000308937 |
| ARX | ENSP00000368332 |
| CLN5 | ENSP00000366673 |
| TBP | ENSP00000230354 |
| JPH3 | ENSP00000284262 |
| SCN4A | ENSP00000396320 |
| ATN1 | ENSP00000349076 |
| CDK5RAP2 | ENSP00000343818 |
| PPT1 | ENSP00000394863 |
| EIF2B3 | ENSP00000353575 |
| IGBP1 | ENSP00000348784 |
| SPTLC1 | ENSP00000262554 |
| ATXN2 | ENSP00000366843 |
| MR1 | ENSP00000356552 |
| ABCD1 | ENSP00000218104 |
| CLN8 | ENSP00000328182 |
| PRSS12 | ENSP00000296498 |
| PARK2 | ENSP00000355865 |
| NDUFA2 | ENSP00000252102 |
| ROBO3 | ENSP00000380903 |
| HCFC1 | ENSP00000309555 |
| ATCAY | ENSP00000390941 |
| SPG21 | ENSP00000204566 |
| GALC | ENSP00000261304 |
| SDHA | ENSP00000264932 |
| FXN | ENSP00000366482 |
| ASPM | ENSP00000356379 |
| BLMH | ENSP00000261714 |
| ALS2 | ENSP00000264276 |
| IKBKAP | ENSP00000363779 |
| BCS1L | ENSP00000352219 |
| OPHN1 | ENSP00000347710 |
| SNAP29 | ENSP00000215730 |
| SACS | ENSP00000371729 |
| EFHC1 | ENSP00000360107 |
| CRBN | ENSP00000231948 |
| SPG7 | ENSP00000268704 |
| MPO | ENSP00000225275 |
| GPR56 | ENSP00000369018 |
| SCN1B | ENSP00000396915 |
| NDUFA12 | ENSP00000330737 |
| VAPB | ENSP00000417175 |
| POLG | ENSP00000268124 |
| EIF2B4 | ENSP00000394869 |
| SURF1 | ENSP00000361042 |
| L1CAM | ENSP00000359074 |
| CLN3 | ENSP00000454253 |
| SETX | ENSP00000224140 |
| PAK3 | ENSP00000353864 |
| NDUFS4 | ENSP00000296684 |
| PEX12 | ENSP00000225873 |
| NDUFS7 | ENSP00000233627 |
| ARHGEF6 | ENSP00000250617 |
| COX15 | ENSP00000016171 |
| MCPH1 | ENSP00000342924 |
| FTSJ1 | ENSP00000326948 |
| NDUFA10 | ENSP00000252711 |
| TIMM8A | ENSP00000361993 |
| EIF2B2 | ENSP00000266126 |
| NDUFA9 | ENSP00000266544 |
| CHM | ENSP00000350386 |
| NDUFS8 | ENSP00000315774 |
| NDUFS3 | ENSP00000263774 |
| TTF1 | ENSP00000333920 |

1. Disease genes of nutritional

| **Gene symbol** | **Ensembl ID** |
| --- | --- |
| UCP1 | ENSP00000262999 |
| POMC | ENSP00000264708 |
| PPARG | ENSP00000287820 |
| AGRP | ENSP00000290953 |
| MC4R | ENSP00000299766 |
| PCSK1 | ENSP00000308024 |
| PPARGC1B | ENSP00000312649 |
| UCP3 | ENSP00000323740 |
| GHRL | ENSP00000335074 |
| ADRB3 | ENSP00000343782 |
| PYY | ENSP00000353198 |
| ENPP1 | ENSP00000354238 |
| NR0B2 | ENSP00000254227 |
| SIM1 | ENSP00000262901 |
| HTR2A | ENSP00000367959 |
| SDC3 | ENSP00000344468 |
| ADRB2 | ENSP00000305372 |
| SLC6A14 | ENSP00000360967 |

1. Disease genes of ophthamological

| **Gene symbol** | **Ensembl ID** |
| --- | --- |
| PLG | ENSP00000308938 |
| CFHR1 | ENSP00000314299 |
| FOXE3 | ENSP00000334472 |
| CFHR3 | ENSP00000356395 |
| GJA3 | ENSP00000241125 |
| PITX3 | ENSP00000359019 |
| CYP1B1 | ENSP00000260630 |
| PRPH | ENSP00000257860 |
| APOE | ENSP00000252486 |
| TGFBI | ENSP00000416330 |
| PHOX2A | ENSP00000298231 |
| VSX1 | ENSP00000365899 |
| PAX6 | ENSP00000368401 |
| SAG | ENSP00000386444 |
| TACSTD2 | ENSP00000360269 |
| NDP | ENSP00000367301 |
| OPN1MW | ENSP00000358951 |
| LRP5 | ENSP00000294304 |
| RGS9BP | ENSP00000334134 |
| AIM1 | ENSP00000358062 |
| VCAN | ENSP00000265077 |
| KRT12 | ENSP00000251643 |
| HSF4 | ENSP00000264009 |
| DRD5 | ENSP00000306129 |
| CYP4V2 | ENSP00000368079 |
| PIKFYVE | ENSP00000264380 |
| SOX2 | ENSP00000323588 |
| GJA8 | ENSP00000240986 |
| KRT3 | ENSP00000413479 |
| ABCA4 | ENSP00000359245 |
| TIMP3 | ENSP00000266085 |
| OPN1LW | ENSP00000358967 |
| RGS9 | ENSP00000262406 |
| RPGRIP1 | ENSP00000382895 |
| FBLN5 | ENSP00000345008 |
| ELOVL4 | ENSP00000358831 |
| FOXC1 | ENSP00000370256 |
| FBN1 | ENSP00000325527 |
| EFEMP1 | ENSP00000347596 |
| FTL | ENSP00000366525 |
| PLEKHA1 | ENSP00000357986 |
| ROM1 | ENSP00000278833 |
| HMCN1 | ENSP00000271588 |
| SPATA7 | ENSP00000377176 |
| CRYGC | ENSP00000282141 |
| OPTN | ENSP00000263036 |
| KERA | ENSP00000266719 |
| SLC45A2 | ENSP00000296589 |
| BEST1 | ENSP00000399709 |
| RHO | ENSP00000296271 |
| FZD4 | ENSP00000434034 |
| CRYBA1 | ENSP00000225387 |
| CRYBB2 | ENSP00000381273 |
| CHST6 | ENSP00000328983 |
| PRPH2 | ENSP00000230381 |
| RLBP1 | ENSP00000268125 |
| CNGB3 | ENSP00000316605 |
| PITX2 | ENSP00000304169 |
| NHS | ENSP00000369400 |
| AIPL1 | ENSP00000370521 |
| CA4 | ENSP00000300900 |
| C1QTNF5 | ENSP00000402389 |
| CRYAA | ENSP00000291554 |
| COL8A2 | ENSP00000305913 |
| RPE65 | ENSP00000262340 |
| IMPDH1 | ENSP00000345096 |
| RP9 | ENSP00000297157 |
| CRX | ENSP00000221996 |
| CTDP1 | ENSP00000299543 |
| NYX | ENSP00000340328 |
| PRPF8 | ENSP00000304350 |
| MYOC | ENSP00000037502 |
| PRPF31 | ENSP00000324122 |
| TULP1 | ENSP00000229771 |
| CRYGD | ENSP00000264376 |
| OPA1 | ENSP00000354681 |
| CERKL | ENSP00000341159 |
| PDE6B | ENSP00000420295 |
| GUCY2D | ENSP00000254854 |
| LAMB2 | ENSP00000307156 |
| CNGA3 | ENSP00000272602 |
| CRB1 | ENSP00000356370 |
| RDH5 | ENSP00000257895 |
| OPA3 | ENSP00000319817 |
| POLG | ENSP00000268124 |
| CACNA1F | ENSP00000365441 |
| RP1 | ENSP00000220676 |
| KIF21A | ENSP00000354878 |
| FSCN2 | ENSP00000334665 |
| RPGR | ENSP00000367766 |
| GUCA1A | ENSP00000053469 |
| CHM | ENSP00000350386 |

1. Disease genes of psychiatric

| **Gene symbol** | **Ensembl ID** |
| --- | --- |
| DRD4 | ENSP00000176183 |
| SLC6A4 | ENSP00000261707 |
| AKT1 | ENSP00000270202 |
| APOL4 | ENSP00000338260 |
| COMT | ENSP00000354511 |
| BDNF | ENSP00000414303 |
| SLC6A3 | ENSP00000270349 |
| CHI3L1 | ENSP00000255409 |
| APOL2 | ENSP00000249066 |
| HCRT | ENSP00000293330 |
| HTR2A | ENSP00000367959 |
| DAOA | ENSP00000365103 |
| GABBR2 | ENSP00000259455 |
| DRD3 | ENSP00000373169 |
| TPH2 | ENSP00000329093 |
| DLX2 | ENSP00000234198 |
| DRD5 | ENSP00000306129 |
| CHRNA4 | ENSP00000359285 |
| PRNP | ENSP00000368752 |
| PRODH | ENSP00000349577 |
| RTN4R | ENSP00000043402 |
| DISC1 | ENSP00000355593 |
| HTT | ENSP00000347184 |
| NLGN3 | ENSP00000351591 |
| DYX1C1 | ENSP00000323275 |
| MTHFR | ENSP00000365775 |
| DAO | ENSP00000228476 |
| KIAA0319 | ENSP00000367459 |
| CYP2A6 | ENSP00000301141 |
| DTNBP1 | ENSP00000341680 |
| PRODH2 | ENSP00000301175 |

1. Disease genes of renal

| **Gene symbol** | **Ensembl ID** |
| --- | --- |
| REN | ENSP00000272190 |
| AGTR1 | ENSP00000273430 |
| ACE | ENSP00000290866 |
| AVPR2 | ENSP00000338072 |
| SLC4A5 | ENSP00000350475 |
| CD2AP | ENSP00000352264 |
| TRPM6 | ENSP00000354006 |
| AGT | ENSP00000355627 |
| ACE | ENSP00000464149 |
| COL4A4 | ENSP00000379866 |
| FOXL2 | ENSP00000333188 |
| FXYD2 | ENSP00000292079 |
| SLC7A9 | ENSP00000023064 |
| COL4A3 | ENSP00000379823 |
| SLC5A2 | ENSP00000327943 |
| INSL3 | ENSP00000321724 |
| UMOD | ENSP00000306279 |
| SLC22A12 | ENSP00000366797 |
| INVS | ENSP00000262457 |
| ACTN4 | ENSP00000252699 |
| NPHS1 | ENSP00000368190 |
| IQCB1 | ENSP00000311505 |
| NPHP3 | ENSP00000338766 |
| CLCN5 | ENSP00000365256 |
| HNF1B | ENSP00000225893 |
| NPHS2 | ENSP00000356587 |
| WT1 | ENSP00000331327 |
| ATP6V0A4 | ENSP00000253856 |
| SLC3A1 | ENSP00000260649 |
| ZNF365 | ENSP00000387091 |
| SCNN1G | ENSP00000300061 |
| CACNA1S | ENSP00000355192 |
| TRPC6 | ENSP00000340913 |
| NPHP4 | ENSP00000367398 |
| PKHD1 | ENSP00000360158 |
| SLC12A3 | ENSP00000402152 |
| SCNN1B | ENSP00000345751 |
| NPHP1 | ENSP00000313169 |
| COL4A5 | ENSP00000331902 |
| SLC4A4 | ENSP00000393557 |
| CTNS | ENSP00000371294 |
| CD151 | ENSP00000324101 |
| CLDN16 | ENSP00000264734 |
| PKD1 | ENSP00000262304 |
| DIAPH2 | ENSP00000321348 |

1. Disease genes of respiratory

| **Gene symbol** | **Ensembl ID** |
| --- | --- |
| TSC2 | ENSP00000219476 |
| MC3R | ENSP00000243911 |
| SCGB1A1 | ENSP00000278282 |
| HNMT | ENSP00000280097 |
| TSC1 | ENSP00000298552 |
| PTGDR | ENSP00000303424 |
| BDNF | ENSP00000414303 |
| FLCN | ENSP00000285071 |
| TNF | ENSP00000398698 |
| CISH | ENSP00000409346 |
| IFNG | ENSP00000229135 |
| PHOX2B | ENSP00000226382 |
| FCGR2A | ENSP00000271450 |
| TLR2 | ENSP00000260010 |
| SLC11A1 | ENSP00000233202 |
| SFTPB | ENSP00000377409 |
| HMOX1 | ENSP00000216117 |
| ALOX5 | ENSP00000363512 |
| EDN3 | ENSP00000337128 |
| RET | ENSP00000347942 |
| ADRB2 | ENSP00000305372 |
| CCL2 | ENSP00000225831 |
| MMP1 | ENSP00000322788 |
| IFNGR1 | ENSP00000356713 |
| PLA2G7 | ENSP00000274793 |
| TGFB1 | ENSP00000221930 |
| IL12B | ENSP00000231228 |
| CCL11 | ENSP00000302234 |
| GDNF | ENSP00000409007 |
| CD209 | ENSP00000315477 |
| CFTR | ENSP00000003084 |
| IL13 | ENSP00000304915 |
| SERPINA1 | ENSP00000348068 |
| IRGM | ENSP00000428220 |
| TIRAP | ENSP00000376445 |
| ASCL1 | ENSP00000266744 |
| SCGB3A2 | ENSP00000296694 |
| SFTPA1 | ENSP00000397082 |
| SFTPA2 | ENSP00000361400 |
| SP110 | ENSP00000258381 |
| MUC5B | ENSP00000436812 |
| HLA-G | ENSP00000353472 |
| NPSR1 | ENSP00000352839 |
| FLCN | ENSP00000394249 |
| MUC7 | ENSP00000302021 |
| PHF11 | ENSP00000367570 |
| DNAH5 | ENSP00000265104 |

1. Disease genes of skeletal

| **Gene symbol** | **Ensembl ID** |
| --- | --- |
| SCT | ENSP00000176195 |
| EFNB1 | ENSP00000204961 |
| DLL3 | ENSP00000205143 |
| TGFB1 | ENSP00000221930 |
| COMP | ENSP00000222271 |
| GHR | ENSP00000230882 |
| MSX2 | ENSP00000239243 |
| TBX4 | ENSP00000240335 |
| GHSR | ENSP00000241256 |
| SOX9 | ENSP00000245479 |
| HOXD10 | ENSP00000249501 |
| MNX1 | ENSP00000252971 |
| CILP | ENSP00000261883 |
| BMPR1B | ENSP00000264568 |
| TP63 | ENSP00000264731 |
| EVC | ENSP00000264956 |
| DYM | ENSP00000269445 |
| CTSK | ENSP00000271651 |
| LBR | ENSP00000272163 |
| GJA1 | ENSP00000282561 |
| SLC26A2 | ENSP00000286298 |
| IHH | ENSP00000295731 |
| SHH | ENSP00000297261 |
| SOST | ENSP00000301691 |
| LMBR1 | ENSP00000326604 |
| NOG | ENSP00000328181 |
| FBLN1 | ENSP00000331544 |
| SRA1 | ENSP00000337513 |
| FGFR3 | ENSP00000339824 |
| NPR2 | ENSP00000341083 |
| COL9A3 | ENSP00000341640 |
| EVC2 | ENSP00000342144 |
| MESP2 | ENSP00000342392 |
| OFD1 | ENSP00000344314 |
| WISP3 | ENSP00000354734 |
| PAX9 | ENSP00000355245 |
| THBS2 | ENSP00000355751 |
| IRF6 | ENSP00000355988 |
| FLNA | ENSP00000358866 |
| COL11A1 | ENSP00000359114 |
| RUNX2 | ENSP00000360493 |
| COL9A2 | ENSP00000361834 |
| GDF5 | ENSP00000363489 |
| ASPN | ENSP00000364694 |
| ROR2 | ENSP00000364860 |
| BMP2 | ENSP00000368104 |
| COL2A1 | ENSP00000369889 |
| SHOX | ENSP00000370990 |
| MSX1 | ENSP00000372170 |
| HOXD13 | ENSP00000376322 |
| GLI3 | ENSP00000379258 |
| MATN3 | ENSP00000383894 |
| ACAN | ENSP00000387356 |
| TRAPPC2 | ENSP00000392495 |
| FGFR1 | ENSP00000393312 |
| PRG4 | ENSP00000399679 |
| FGFR2 | ENSP00000410294 |
| FLNB | ENSP00000420213 |

1. Unclassified

| **Gene symbol** | **Ensembl ID** |
| --- | --- |
| CFTR | ENSP00000003084 |
| TAS2R16 | ENSP00000249284 |
| RCBTB1 | ENSP00000258646 |
| PLOD2 | ENSP00000282903 |
| LRP5 | ENSP00000294304 |
| ADH1B | ENSP00000306606 |
| GABRA2 | ENSP00000348897 |
| TSPYL1 | ENSP00000357597 |
| PTEN | ENSP00000361021 |
| HTR2A | ENSP00000367959 |
| FSHR | ENSP00000384708 |
| FGFR2 | ENSP00000410294 |
| SH3BP2 | ENSP00000422168 |
