# Supplementary material for: Deciphering the Relationship between Obesity and Various Diseases from a Network Perspective
Source: Genes (Basel). 2017 Dec 18;8(12):392. doi: 10.3390/genes8120392 (PMC5748710; doi:10.3390/genes8120392)
Supplement: Supplementary file 1 [file genes-08-00392-s001.zip › Table_S2.docx]

**Table S2.** Gene symbols of 379 obesity genes and their Ensembl IDs.

| **Gene symbol** | **Ensembl ID** |
| --- | --- |
| ACE | ENSP00000290866 ENSP00000464149 |
| ADA | ENSP00000361965 |
| AGT | ENSP00000355627 |
| AK1 | ENSP00000362249 |
| AR | ENSP00000363822 |
| BF | - |
| C3 | ENSP00000245907 |
| CBL | ENSP00000264033 |
| CHM | ENSP00000350386 |
| CPE | ENSP00000386104 |
| CRH | ENSP00000276571 |
| DBH | ENSP00000376776 |
| DF | - |
| DPT | ENSP00000356791 |
| ESD | ENSP00000367992 |
| GAL | ENSP00000265643 |
| GCK | ENSP00000223366 |
| GH1 | ENSP00000312673 |
| GHR | ENSP00000230882 |
| HD | - |
| HDC | ENSP00000267845 |
| IDE | ENSP00000265986 |
| IL6 | ENSP00000258743 |
| INS | ENSP00000250971 |
| IPW | - |
| KEL | ENSP00000347409 |
| LEP | ENSP00000312652 |
| LHB | ENSP00000221421 |
| LPL | ENSP00000309757 |
| LTA | ENSP00000403495 |
| MYC | ENSP00000367207 |
| NDN | ENSP00000332643 |
| NMB | ENSP00000378089 |
| NMU | ENSP00000264218 |
| NPB | ENSP00000332766 |
| NPY | ENSP00000242152 |
| PAH | ENSP00000448059 |
| PGD | ENSP00000270776 |
| PGR | ENSP00000325120 |
| POR | ENSP00000419970 |
| PPY | ENSP00000225992 |
| PYY | ENSP00000353198 |
| REN | ENSP00000272190 |
| SAH | - |
| SGK | - |
| TH | ENSP00000370571 |
| TKT | ENSP00000405455 |
| TNF | ENSP00000398698 |
| TUB | ENSP00000305426 |
| VDR | ENSP00000447173 |
| VGF | ENSP00000249330 |
| WT1 | ENSP00000331327 |
| WTS | - |
| A4GALT | ENSP00000249005 |
| ABCA7 | ENSP00000263094 |
| ABCC8 | ENSP00000374467 |
| ABCG5 | ENSP00000260645 |
| ACACB | ENSP00000341044 |
| ACADVL | ENSP00000349297 |
| ACP1 | ENSP00000272065 |
| ADAM12 | ENSP00000357668 |
| ADAMTS1 | ENSP00000284984 |
| ADCYAP1 | ENSP00000411658 |
| ADIPOQ | ENSP00000320709 |
| ADRA1B | ENSP00000306662 |
| ADRA2A | ENSP00000280155 |
| ADRA2B | ENSP00000387281 |
| ADRB1 | ENSP00000358301 |
| ADRB2 | ENSP00000305372 |
| ADRB3 | ENSP00000343782 |
| AEBP1 | ENSP00000223357 |
| AGPAT2 | ENSP00000360761 |
| AGRP | ENSP00000290953 |
| AGTR2 | ENSP00000360973 |
| AHO2 | - |
| AHSG | ENSP00000393887 |
| AKT1 | ENSP00000270202 |
| AKT2 | ENSP00000375892 |
| ALMS1 | ENSP00000264448 |
| ALPI | ENSP00000295463 |
| AMACR | ENSP00000371517 |
| ANGPTL4 | ENSP00000301455 |
| ANGPTL6 | ENSP00000253109 |
| ANMA | - |
| APOA1 | ENSP00000236850 |
| APOA2 | ENSP00000356969 |
| APOA4 | ENSP00000350425 |
| APOA5 | ENSP00000227665 |
| APOB | ENSP00000233242 |
| APOC1 | ENSP00000252491 |
| APOC3 | ENSP00000227667 |
| APOD | ENSP00000345179 |
| APOE | ENSP00000252486 |
| AQP7 | ENSP00000297988 |
| ARID5B | ENSP00000279873 |
| ARL6 | ENSP00000337722 |
| ASIP | ENSP00000364092 |
| ASPA | ENSP00000263080 |
| ATP12A | ENSP00000218548 |
| ATP1A2 | ENSP00000354490 |
| ATP1B1 | ENSP00000356789 |
| ATP8B1 | ENSP00000283684 |
| BATF | ENSP00000286639 |
| BBS1 | ENSP00000317469 ENSP00000398526 |
| BBS2 | ENSP00000245157 |
| BBS4 | ENSP00000268057 |
| BBS5 | ENSP00000295240 ENSP00000424363 |
| BBS7 | ENSP00000264499 |
| BDNF | ENSP00000414303 |
| BRS3 | ENSP00000359682 |
| BSCL2 | ENSP00000354032 |
| BUB1B | ENSP00000287598 |
| CAPN10 | ENSP00000375844 |
| CART | - |
| CAV1 | ENSP00000339191 |
| CAV3 | ENSP00000341940 |
| CBFA2T1 | - |
| CCKAR | ENSP00000295589 |
| CCKBR | ENSP00000335544 |
| CCND3 | ENSP00000362082 |
| CD36 | ENSP00000308165 |
| CDH2 | ENSP00000269141 |
| CDKN1A | ENSP00000244741 |
| CDKN1B | ENSP00000228872 |
| CEBPA | ENSP00000427514 |
| CEBPB | ENSP00000305422 |
| CEBPD | ENSP00000386165 |
| CHRM3 | ENSP00000255380 |
| CIDEA | ENSP00000320209 |
| CLOCK | ENSP00000308741 |
| CNR1 | ENSP00000358511 |
| CNTFR | ENSP00000242338 |
| COH1 | - |
| COL8A2 | ENSP00000305913 |
| COMT | ENSP00000354511 |
| CORIN | ENSP00000273857 |
| CPT1A | ENSP00000265641 |
| CRHR1 | ENSP00000381333 |
| CRHR2 | ENSP00000340943 |
| CYB5R4 | ENSP00000358695 |
| CYP11B2 | ENSP00000325822 |
| CYP19A1 | ENSP00000260433 |
| CYP2D6 | ENSP00000353820 |
| CYP7A1 | ENSP00000301645 |
| DGAT1 | ENSP00000332258 |
| DHCR24 | ENSP00000360316 |
| DIO1 | ENSP00000354643 |
| DLK1 | ENSP00000340292 |
| DNAJC3 | ENSP00000365991 |
| DRD2 | ENSP00000354859 |
| DRD3 | ENSP00000373169 |
| DRD4 | ENSP00000176183 |
| ENPP1 | ENSP00000354238 |
| EREG | ENSP00000244869 |
| ESR1 | ENSP00000206249 |
| ESR2 | ENSP00000343925 |
| ESRRA | ENSP00000000442 |
| FABP1 | ENSP00000295834 |
| FABP2 | ENSP00000274024 |
| FABP4 | ENSP00000256104 |
| FABP5 | ENSP00000297258 |
| FASN | ENSP00000304592 |
| FGF21 | ENSP00000222157 |
| FGFR3 | ENSP00000339824 |
| FKHL18 | - |
| FMR1 | ENSP00000359506 |
| FOSB | ENSP00000245919 |
| FOXA2 | ENSP00000315955 |
| FOXC2 | ENSP00000326371 |
| FSHR | ENSP00000384708 |
| FXYD4 | - |
| GABRG3 | ENSP00000331912 |
| GAD2 | ENSP00000259271 |
| GAMT | ENSP00000403536 |
| GAS6 | ENSP00000331831 |
| GAST | ENSP00000331358 |
| GCGR | ENSP00000383558 |
| GDF3 | ENSP00000331745 |
| GDF8 | - |
| GFPT1 | ENSP00000354347 |
| GFRA2 | ENSP00000428518 |
| GHRH | ENSP00000237527 |
| GHRHR | ENSP00000320180 |
| GHRL | ENSP00000335074 |
| GHSR | ENSP00000241256 |
| GIPR | ENSP00000467494 |
| GLO1 | ENSP00000362463 |
| GNAS | ENSP00000360141 |
| GNB3 | ENSP00000229264 |
| GNG3 | ENSP00000294117 |
| GPAM | ENSP00000265276 |
| GPC1 | ENSP00000264039 |
| GPC3 | ENSP00000377836 |
| GPC4 | ENSP00000359864 |
| GPD2 | ENSP00000308610 |
| GPHB5 | - |
| GPR10 | - |
| GPR109A | - |
| GPR24 | - |
| GPR35 | ENSP00000415890 |
| GPR40 | - |
| GPR7 | - |
| GPX1 | ENSP00000407375 |
| GRM5 | ENSP00000306138 |
| GRM8 | ENSP00000344173 |
| GSK3B | ENSP00000324806 |
| GYPA | ENSP00000354003 |
| GYS1 | ENSP00000317904 |
| H6PD | ENSP00000366620 |
| HCRT | ENSP00000293330 |
| HEXB | ENSP00000261416 |
| HMGA2 | ENSP00000384026 |
| HRH1 | ENSP00000380247 |
| HRH3 | ENSP00000342560 |
| HSD11B1 | ENSP00000261465 |
| HSD11B2 | ENSP00000316786 |
| HSD3B1 | ENSP00000358421 |
| HSPA1B | ENSP00000364801 |
| HTR1B | ENSP00000358963 |
| HTR2A | ENSP00000367959 |
| HTR2C | ENSP00000276198 |
| ICAM1 | ENSP00000264832 |
| IDH1 | ENSP00000260985 |
| IFRD1 | ENSP00000005558 |
| IGF1 | ENSP00000302665 |
| IGF2 | ENSP00000391826 |
| IGFBP6 | ENSP00000301464 |
| IGKC | - |
| IL1RN | ENSP00000259206 |
| IL6R | ENSP00000357470 |
| INPPL1 | ENSP00000298229 |
| INSR | ENSP00000303830 |
| IRS1 | ENSP00000304895 |
| IRS2 | ENSP00000365016 |
| ISL1 | ENSP00000230658 |
| KCNA3 | ENSP00000358784 |
| KCNJ11 | ENSP00000345708 |
| KLF5 | ENSP00000366915 |
| LDLR | ENSP00000454071 |
| LEPR | ENSP00000330393 |
| LIPA | ENSP00000337354 |
| LIPC | ENSP00000299022 |
| LIPE | ENSP00000244289 |
| LMNA | ENSP00000357283 |
| LPIN1 | ENSP00000256720 |
| LRPAP1 | ENSP00000421922 |
| MACS2 | - |
| MAGEL2 | ENSP00000433433 |
| MAOA | ENSP00000340684 |
| MAPK3 | ENSP00000263025 |
| MC3R | ENSP00000243911 |
| MC4R | ENSP00000299766 |
| MC5R | ENSP00000318077 |
| MECP2 | ENSP00000395535 |
| MED12 | ENSP00000363193 |
| MEHMO | - |
| MEN1 | ENSP00000337088 |
| MEST | ENSP00000223215 |
| MKKS | ENSP00000246062 |
| MKRN3 | ENSP00000313881 |
| MLXIPL | ENSP00000320886 |
| MMP11 | ENSP00000215743 |
| MMP19 | ENSP00000313437 |
| MRXS11 | - |
| MRXS7 | - |
| MT1A | ENSP00000290705 |
| MTTP | ENSP00000265517 |
| NCB5OR | - |
| NCOA3 | ENSP00000361066 |
| NHLH2 | ENSP00000322087 |
| NOS2A | - |
| NPR3 | ENSP00000265074 |
| NPY1R | ENSP00000354652 |
| NPY2R | ENSP00000332591 |
| NPY5R | ENSP00000339377 |
| NR0B2 | ENSP00000254227 |
| NR1H2 | ENSP00000253727 |
| NR1I3 | ENSP00000356958 |
| NR3C1 | ENSP00000231509 |
| NTRK2 | ENSP00000277120 |
| NTSR1 | ENSP00000359532 |
| OPRM1 | ENSP00000394624 |
| ORM1 | ENSP00000259396 |
| PARP1 | ENSP00000355759 |
| PAX6 | ENSP00000368401 |
| PCSK1 | ENSP00000308024 |
| PCSK1N | ENSP00000218230 |
| PEG3 | ENSP00000326581 |
| PEMT | ENSP00000255389 |
| PHF6 | ENSP00000329097 |
| PIK3R1 | ENSP00000274335 |
| PIP5K2B | - |
| PLA2G1B | ENSP00000312286 |
| PLIN | - |
| PLSCR1 | ENSP00000345494 |
| PLSCR3 | ENSP00000316021 |
| PMCH | ENSP00000332225 |
| PMM2 | ENSP00000268261 |
| PNMT | ENSP00000269582 |
| POMC | ENSP00000264708 |
| PON1 | ENSP00000222381 |
| PON2 | ENSP00000222572 |
| PPARA | ENSP00000262735 |
| PPARD | ENSP00000310928 |
| PPARG | ENSP00000287820 |
| PPP1R3A | ENSP00000284601 |
| PPP1R3C | ENSP00000238994 |
| PRKAA2 | ENSP00000360290 |
| PRKAG3 | ENSP00000233944 |
| PRKAR1A | ENSP00000351410 |
| PRKAR2B | ENSP00000265717 |
| PRKCQ | ENSP00000263125 |
| PRLHR | ENSP00000239032 |
| PROP1 | ENSP00000311290 |
| PROX1 | ENSP00000261454 |
| PTPN1 | ENSP00000360683 |
| PTPN11 | ENSP00000340944 |
| PTPNS1 | - |
| PTPRF | ENSP00000353030 |
| PTTG1 | ENSP00000344936 |
| PWCR1 | - |
| PWLSX | - |
| RAI1 | ENSP00000323074 |
| RASGRF1 | ENSP00000405963 |
| RETN | ENSP00000221515 |
| RETNLB | ENSP00000295755 |
| RPS6KA3 | ENSP00000368884 |
| RPS6KB1 | ENSP00000225577 |
| RSC1A1 | ENSP00000341963 |
| RXRG | ENSP00000352900 |
| SCARB1 | ENSP00000261693 |
| SCD1 | - |
| SDC1 | ENSP00000254351 |
| SDC3 | ENSP00000344468 |
| SFRP1 | ENSP00000220772 |
| SGBS2 | - |
| SH2B | - |
| SIM1 | ENSP00000262901 |
| SLC2A2 | ENSP00000323568 |
| SLC2A4 | ENSP00000320935 |
| SLC6A1 | ENSP00000287766 |
| SLC6A14 | ENSP00000360967 |
| SLC6A3 | ENSP00000270349 |
| SNRPN | ENSP00000306223 |
| SOAT2 | ENSP00000301466 |
| SOCS3 | ENSP00000330341 |
| SORBS1 | ENSP00000355136 ENSP00000360272 |
| SOX8 | ENSP00000293894 |
| SPARC | ENSP00000231061 |
| SREBF1 | ENSP00000348069 |
| STAT3 | ENSP00000264657 |
| STAT5B | ENSP00000293328 |
| STK25 | ENSP00000325748 |
| TBX3 | ENSP00000257566 |
| TCF1 | - |
| TGFB1 | ENSP00000221930 |
| THRA | ENSP00000264637 |
| THRB | ENSP00000348827 |
| TXNIP | ENSP00000358323 |
| UBL5 | ENSP00000351492 |
| UBR1 | ENSP00000290650 |
| UCP1 | ENSP00000262999 |
| UCP2 | ENSP00000312029 |
| UCP3 | ENSP00000323740 |
| UNC5C | ENSP00000406022 |
| VLDLR | ENSP00000371532 |
| VSX1 | ENSP00000365899 |
| WBSCR14 | - |
| WNT10B | ENSP00000301061 |
| ZFP36 | ENSP00000248673 |
| BBS3(ARL6) | - |
| BBS8(TTC8) | - |
| C19orf20 | - |
| EIF4EBP1 | ENSP00000340691 |
| MAPK8IP1 | ENSP00000241014 |
| PPARGC1A | ENSP00000264867 |
| PPARGC1B | ENSP00000312649 |
| SERPINE1 | ENSP00000223095 |
| TNFRSF1B | ENSP00000365435 |
