# Supplementary material for: Deciphering the Relationship between Obesity and Various Diseases from a Network Perspective
Source: Genes (Basel). 2017 Dec 18;8(12):392. doi: 10.3390/genes8120392 (PMC5748710; doi:10.3390/genes8120392)
Supplement: Supplementary file 1 [file genes-08-00392-s001.zip › Table_S3.docx]

**Table S3.** Maximum interaction scores and p-value for each disease gene of 22 disease classes.

1. Disease genes of bone

| **Gene symbol** | **MIS** | **P-value** |
| --- | --- | --- |
| ALPL | 969 | <0.001 |
| ANKH | 610 | 0.015 |
| TNFRSF11B | 895 | 0.034 |
| EXT1 | 968 | 0.043 |
| EIF2AK3 | 993 | 0.054 |
| EXT2 | 970 | 0.056 |
| CYP2R1 | 700 | 0.060 |
| GNAS | 988 | 0.061 |
| DLX3 | 832 | 0.068 |
| WISP3 | 458 | 0.086 |
| LRP5 | 992 | 0.090 |
| KLK4 | 824 | 0.090 |
| VDR | 992 | 0.099 |
| FGF23 | 965 | 0.103 |
| CLCN7 | 644 | 0.118 |
| COL1A1 | 951 | 0.129 |
| TREM2 | 900 | 0.131 |
| COL9A3 | 910 | 0.149 |
| COMP | 840 | 0.150 |
| LEMD3 | 724 | 0.156 |
| CALCR | 933 | 0.171 |
| ANO5 | 499 | 0.194 |
| ENAM | 590 | 0.204 |
| TNFRSF11A | 580 | 0.246 |
| TCIRG1 | 953 | 0.271 |
| COL9A2 | 908 | 0.272 |
| KL | 873 | 0.275 |
| DSPP | 519 | 0.338 |
| AMELX | 433 | 0.370 |
| COL11A2 | 568 | 0.371 |
| MATN3 | 400 | 0.384 |
| COL1A2 | 921 | 0.395 |
| TYROBP | 902 | 0.400 |
| COL2A1 | 900 | 0.546 |
| SQSTM1 | 827 | 0.642 |
| GALNT3 | 503 | 0.652 |
| SLC26A2 | 220 | 0.867 |

1. Disease genes of cancer

| **Gene symbol** | **MIS** | **P-value** |
| --- | --- | --- |
| ESR1 | 999 | <0.001 |
| CCND1 | 999 | <0.001 |
| APC | 999 | <0.001 |
| PDGFRB | 999 | <0.001 |
| MSR1 | 931 | <0.001 |
| AXIN1 | 999 | <0.001 |
| EP300 | 999 | <0.001 |
| PIK3CA | 999 | <0.001 |
| TP53 | 999 | <0.001 |
| ERBB2 | 999 | <0.001 |
| AKT1 | 999 | <0.001 |
| EGFR | 999 | <0.001 |
| KIT | 999 | <0.001 |
| ASPSCR1 | 990 | <0.001 |
| AXIN2 | 999 | <0.001 |
| MN1 | 902 | <0.001 |
| TERT | 999 | <0.001 |
| PPP2R1B | 999 | <0.001 |
| MET | 999 | <0.001 |
| PLAG1 | 862 | <0.001 |
| TRIM24 | 998 | <0.001 |
| PTPN11 | 999 | <0.001 |
| RNF6 | 926 | <0.001 |
| CTNNB1 | 999 | <0.001 |
| IGF2R | 999 | <0.001 |
| MAX | 999 | <0.001 |
| TGFBR2 | 999 | <0.001 |
| PTEN | 999 | <0.001 |
| AR | 999 | <0.001 |
| MYC | 999 | <0.001 |
| JAK2 | 999 | <0.001 |
| CDKN2A | 999 | <0.001 |
| NCOA4 | 576 | <0.001 |
| BRCA1 | 999 | <0.001 |
| CEBPA | 999 | <0.001 |
| GPC3 | 990 | 0.009 |
| FLT3 | 996 | 0.016 |
| FLCN | 874 | 0.017 |
| RUNX1 | 995 | 0.031 |
| IRF1 | 996 | 0.034 |
| HNF1A | 993 | 0.036 |
| DCC | 990 | 0.041 |
| EXT1 | 968 | 0.043 |
| PCM1 | 981 | 0.044 |
| MXI1 | 814 | 0.045 |
| PDGFRA | 997 | 0.046 |
| MAP3K8 | 973 | 0.051 |
| TLR2 | 992 | 0.052 |
| BCR | 994 | 0.055 |
| GATA2 | 980 | 0.059 |
| KIF1B | 822 | 0.063 |
| KLF6 | 870 | 0.063 |
| CD82 | 921 | 0.073 |
| PLA2G2A | 933 | 0.077 |
| CDS1 | 950 | 0.079 |
| NSD1 | 874 | 0.082 |
| NTRK1 | 997 | 0.087 |
| MAD1L1 | 984 | 0.092 |
| RET | 992 | 0.096 |
| NF2 | 968 | 0.100 |
| NPM1 | 989 | 0.103 |
| POU6F2 | 581 | 0.103 |
| GATA1 | 979 | 0.107 |
| ETV6 | 853 | 0.113 |
| KRAS | 981 | 0.119 |
| BRAF | 993 | 0.138 |
| HMMR | 838 | 0.139 |
| PDGFB | 954 | 0.142 |
| RASA1 | 993 | 0.144 |
| WWOX | 909 | 0.150 |
| KDR | 996 | 0.152 |
| PAX7 | 812 | 0.154 |
| IL1B | 976 | 0.157 |
| DLC1 | 926 | 0.162 |
| PTCH1 | 973 | 0.163 |
| STK11 | 991 | 0.168 |
| NF1 | 942 | 0.180 |
| FGFR3 | 980 | 0.183 |
| GDNF | 919 | 0.185 |
| CCDC6 | 576 | 0.192 |
| NQO2 | 475 | 0.197 |
| RB1CC1 | 866 | 0.198 |
| PTCH2 | 748 | 0.198 |
| ZFHX3 | 639 | 0.204 |
| HNF1B | 725 | 0.208 |
| BAX | 966 | 0.208 |
| RNASEL | 945 | 0.210 |
| ODC1 | 926 | 0.212 |
| GMPS | 975 | 0.212 |
| NRAS | 979 | 0.216 |
| DLEC1 | 503 | 0.220 |
| FLT4 | 944 | 0.224 |
| PAX3 | 875 | 0.229 |
| RASSF1 | 873 | 0.236 |
| IL1RN | 515 | 0.239 |
| AURKA | 994 | 0.240 |
| NME1 | 955 | 0.245 |
| SMARCE1 | 974 | 0.245 |
| WT1 | 926 | 0.246 |
| HRAS | 995 | 0.251 |
| PHB | 878 | 0.256 |
| LPP | 737 | 0.256 |
| COL4A6 | 910 | 0.259 |
| PRKAR1A | 963 | 0.274 |
| PTPRJ | 817 | 0.278 |
| SMAD4 | 997 | 0.282 |
| IRF4 | 920 | 0.289 |
| MSH2 | 968 | 0.292 |
| SH3GL1 | 823 | 0.299 |
| MCC | 282 | 0.303 |
| PTPN12 | 800 | 0.307 |
| IDH1 | 819 | 0.308 |
| SUFU | 788 | 0.316 |
| PMS2 | 786 | 0.317 |
| RNF139 | 718 | 0.329 |
| PPM1D | 713 | 0.330 |
| TLR4 | 961 | 0.338 |
| CASP8 | 977 | 0.342 |
| RB1 | 998 | 0.352 |
| PICALM | 702 | 0.361 |
| TMEM127 | 370 | 0.388 |
| ANTXR1 | 358 | 0.397 |
| SH2D1A | 535 | 0.398 |
| CDH1 | 988 | 0.404 |
| FH | 758 | 0.414 |
| BRCA2 | 947 | 0.415 |
| CBFB | 313 | 0.434 |
| ATM | 984 | 0.438 |
| PRCC | 357 | 0.465 |
| CYP2A6 | 915 | 0.465 |
| CHEK2 | 937 | 0.480 |
| GOLGA5 | 396 | 0.487 |
| VHL | 911 | 0.505 |
| TRIM33 | 658 | 0.509 |
| DIRC2 | 340 | 0.517 |
| TSG101 | 925 | 0.520 |
| BUB1B | 969 | 0.525 |
| BACH1 | 0 | 0.624 |
| OGG1 | 534 | 0.626 |
| PDGFRL | 481 | 0.632 |
| FEZ1 | 233 | 0.637 |
| MLH3 | 575 | 0.651 |
| XRCC3 | 727 | 0.663 |
| NUP214 | 900 | 0.663 |
| CHIC2 | 213 | 0.668 |
| TACC3 | 497 | 0.670 |
| MINPP1 | 354 | 0.677 |
| MLH1 | 773 | 0.685 |
| PARK2 | 792 | 0.685 |
| ARHGAP26 | 576 | 0.697 |
| SDHB | 718 | 0.700 |
| CASP10 | 674 | 0.713 |
| LIG4 | 591 | 0.718 |
| BLM | 854 | 0.726 |
| BARD1 | 771 | 0.745 |
| SDHD | 480 | 0.755 |
| MSH6 | 636 | 0.757 |
| SDHC | 645 | 0.768 |
| PALB2 | 300 | 0.778 |
| SDHD | 407 | 0.779 |
| FLCN | 0 | 0.782 |
| LZTS1 | 242 | 0.815 |
| ERCC6 | 632 | 0.831 |
| ARHGEF12 | 900 | 0.843 |
| MLF1 | 240 | 0.847 |
| ATR | 896 | 0.849 |
| WHSC1L1 | 337 | 0.866 |
| RAD54L | 353 | 0.900 |
| BRIP1 | 303 | 0.952 |
| MUTYH | 258 | 0.960 |
| HIP1 | 288 | 0.974 |
| RAD54B | 266 | 0.997 |

1. Disease genes of cardiovascular

| **Gene symbol** | **MIS** | **P-value** |
| --- | --- | --- |
| ESR1 | 999 | <0.001 |
| RETN | 981 | <0.001 |
| GHR | 999 | <0.001 |
| IL6 | 999 | <0.001 |
| PNMT | 986 | <0.001 |
| AGTR1 | 998 | <0.001 |
| PPARG | 999 | <0.001 |
| NEUROD1 | 994 | <0.001 |
| NOS3 | 999 | <0.001 |
| LIPC | 984 | <0.001 |
| LRP8 | 999 | <0.001 |
| IRS1 | 999 | <0.001 |
| OLR1 | 952 | <0.001 |
| HNF4A | 999 | <0.001 |
| KCNJ11 | 999 | <0.001 |
| GIP | 996 | <0.001 |
| ENPP1 | 979 | <0.001 |
| AGT | 999 | <0.001 |
| APOA2 | 997 | <0.001 |
| PTPN1 | 999 | <0.001 |
| ABCA1 | 999 | <0.001 |
| IRS2 | 999 | <0.001 |
| IGF2BP2 | 989 | <0.001 |
| ACVRL1 | 996 | <0.001 |
| ABCC8 | 999 | <0.001 |
| LDLR | 999 | <0.001 |
| ENG | 990 | 0.015 |
| GCK | 989 | 0.017 |
| ABCC9 | 983 | 0.017 |
| ADRB1 | 943 | 0.017 |
| ECE1 | 856 | 0.017 |
| ATP1B1 | 989 | 0.021 |
| SLC2A4 | 994 | 0.026 |
| PAX4 | 925 | 0.030 |
| LTA | 963 | 0.031 |
| HGF | 992 | 0.033 |
| GJA1 | 994 | 0.033 |
| HNF1A | 993 | 0.036 |
| GPD2 | 924 | 0.038 |
| HMGA1 | 984 | 0.041 |
| DMD | 991 | 0.041 |
| LGALS2 | 585 | 0.042 |
| GCGR | 960 | 0.043 |
| TEK | 996 | 0.049 |
| SGCD | 936 | 0.059 |
| PRKAG2 | 988 | 0.070 |
| KCNJ2 | 907 | 0.071 |
| RGS5 | 747 | 0.079 |
| MAPK8IP1 | 974 | 0.084 |
| AKT2 | 994 | 0.094 |
| EYA4 | 885 | 0.105 |
| SLC6A2 | 750 | 0.106 |
| CDKAL1 | 773 | 0.106 |
| PRKCH | 902 | 0.106 |
| SLC30A8 | 772 | 0.108 |
| MTNR1B | 919 | 0.109 |
| GNAI2 | 849 | 0.121 |
| F13A1 | 936 | 0.124 |
| F7 | 920 | 0.137 |
| NOS2 | 963 | 0.139 |
| TBX1 | 830 | 0.140 |
| F5 | 971 | 0.143 |
| RASA1 | 993 | 0.144 |
| GATA6 | 923 | 0.153 |
| ITIH4 | 899 | 0.171 |
| SELE | 900 | 0.175 |
| GCLC | 850 | 0.180 |
| NR3C2 | 616 | 0.183 |
| LMNA | 881 | 0.207 |
| HNF1B | 725 | 0.208 |
| CYP3A5 | 936 | 0.209 |
| F2 | 977 | 0.234 |
| GCLM | 812 | 0.242 |
| PTGIS | 806 | 0.246 |
| WFS1 | 634 | 0.247 |
| TCF7L2 | 965 | 0.253 |
| DTNA | 660 | 0.255 |
| PDE4D | 914 | 0.268 |
| NKX2-6 | 575 | 0.271 |
| EPHX2 | 508 | 0.273 |
| PRKAR1A | 963 | 0.274 |
| CAV3 | 840 | 0.280 |
| NKX2-5 | 947 | 0.292 |
| GNB3 | 763 | 0.297 |
| MEF2A | 971 | 0.308 |
| PKP2 | 665 | 0.314 |
| CRELD1 | 457 | 0.324 |
| KCNQ1 | 922 | 0.340 |
| JAG1 | 873 | 0.345 |
| ADD1 | 644 | 0.355 |
| TNFSF4 | 504 | 0.361 |
| SCN5A | 816 | 0.402 |
| TCF4 | 846 | 0.409 |
| FH | 758 | 0.414 |
| ALOX5AP | 392 | 0.421 |
| GATA4 | 938 | 0.436 |
| DSP | 654 | 0.438 |
| NAT1 | 399 | 0.439 |
| ZFPM2 | 538 | 0.447 |
| KRIT1 | 487 | 0.466 |
| ELN | 173 | 0.469 |
| TNNT2 | 761 | 0.471 |
| NOTCH3 | 922 | 0.479 |
| KCNMB1 | 370 | 0.482 |
| ANK2 | 643 | 0.488 |
| GDF1 | 463 | 0.495 |
| TPM1 | 900 | 0.555 |
| BMPR2 | 900 | 0.557 |
| ITGB3 | 828 | 0.566 |
| CSRP3 | 396 | 0.605 |
| EPHX1 | 505 | 0.632 |
| CFC1 | 258 | 0.665 |
| RYR2 | 658 | 0.699 |
| MYL3 | 423 | 0.721 |
| PSMA6 | 913 | 0.738 |
| MYBPC3 | 359 | 0.739 |
| DES | 372 | 0.764 |
| TCAP | 396 | 0.790 |
| NET1 | 627 | 0.805 |
| TNNI3 | 637 | 0.818 |
| SCO2 | 325 | 0.844 |
| MYLK2 | 371 | 0.864 |
| LIPH | 0 | 0.865 |
| MYL2 | 506 | 0.867 |
| TTN | 392 | 0.921 |
| MYH7 | 453 | 0.978 |

1. Disease genes of connective tissue disorder

| **Gene symbol** | **MIS** | **P-value** |
| --- | --- | --- |
| IL6 | 999 | <0.001 |
| ENPP1 | 979 | <0.001 |
| TGFBR1 | 999 | <0.001 |
| ANKH | 610 | 0.015 |
| SCT | 970 | 0.019 |
| HLA-DRB1 | 921 | 0.031 |
| HGF | 992 | 0.033 |
| COL3A1 | 951 | 0.047 |
| SLC22A4 | 812 | 0.054 |
| GNPAT | 922 | 0.055 |
| B4GALT7 | 931 | 0.069 |
| IL10 | 979 | 0.069 |
| ASPN | 760 | 0.070 |
| COL5A1 | 932 | 0.077 |
| ABCC6 | 709 | 0.078 |
| KRT4 | 689 | 0.126 |
| COL1A1 | 951 | 0.129 |
| SOS1 | 996 | 0.134 |
| EBP | 398 | 0.135 |
| HLA-B | 922 | 0.159 |
| TNXB | 0 | 0.192 |
| ARSE | 804 | 0.202 |
| FBLN5 | 852 | 0.228 |
| FBN1 | 897 | 0.241 |
| CD244 | 438 | 0.278 |
| FGFR2 | 945 | 0.287 |
| PTPN22 | 634 | 0.318 |
| MIF | 649 | 0.325 |
| FRZB | 651 | 0.353 |
| SMARCAL1 | 567 | 0.357 |
| PLOD1 | 815 | 0.365 |
| COL11A2 | 568 | 0.371 |
| COL1A2 | 921 | 0.395 |
| NFKBIL1 | 349 | 0.418 |
| COL5A2 | 904 | 0.420 |
| ADAMTS2 | 396 | 0.452 |
| ELN | 173 | 0.469 |
| COL6A1 | 906 | 0.470 |
| FLNB | 784 | 0.516 |
| ANTXR2 | 361 | 0.597 |
| ADAMTS10 | 216 | 0.625 |
| KRT13 | 193 | 0.640 |
| XYLT2 | 292 | 0.704 |
| XYLT1 | 256 | 0.770 |
| PADI4 | 242 | 0.809 |
| SLC26A2 | 220 | 0.867 |

1. Disease genes of dermatological

| **Gene symbol** | **MIS** | **P-value** |
| --- | --- | --- |
| TYR | 963 | <0.001 |
| ABCA12 | 917 | <0.001 |
| HR | 883 | <0.001 |
| SOX18 | 933 | 0.012 |
| FLCN | 874 | 0.017 |
| LTA | 963 | 0.031 |
| MBTPS2 | 963 | 0.062 |
| HLA-A | 956 | 0.072 |
| KRT83 | 499 | 0.079 |
| KRT2 | 741 | 0.089 |
| SAT1 | 910 | 0.089 |
| MMP1 | 988 | 0.104 |
| KRT86 | 0 | 0.116 |
| KRT16 | 878 | 0.123 |
| CDSN | 0 | 0.129 |
| SPINK5 | 648 | 0.133 |
| KRT5 | 723 | 0.138 |
| SLURP1 | 497 | 0.140 |
| ZMPSTE24 | 780 | 0.150 |
| CYLD | 939 | 0.152 |
| SLC39A4 | 833 | 0.159 |
| HLA-B | 922 | 0.159 |
| HLA-C | 931 | 0.166 |
| PVRL1 | 909 | 0.170 |
| CDH3 | 906 | 0.180 |
| RAB27A | 933 | 0.194 |
| KRT9 | 482 | 0.207 |
| LMNA | 881 | 0.207 |
| KRT6B | 493 | 0.209 |
| NOD2 | 889 | 0.209 |
| PSTPIP1 | 662 | 0.250 |
| PKP1 | 623 | 0.267 |
| KRT10 | 642 | 0.280 |
| ATP2A2 | 826 | 0.295 |
| KRT6A | 499 | 0.308 |
| KRT17 | 482 | 0.311 |
| ITGA6 | 929 | 0.316 |
| LAMB3 | 906 | 0.331 |
| IKBKG | 983 | 0.337 |
| MYO5A | 914 | 0.337 |
| KRT1 | 613 | 0.342 |
| KRT14 | 580 | 0.371 |
| GJB2 | 578 | 0.375 |
| ITGB4 | 919 | 0.387 |
| ATP2C1 | 475 | 0.407 |
| KRT86 | 235 | 0.411 |
| LAMC2 | 901 | 0.413 |
| ALOX12B | 900 | 0.428 |
| DSP | 654 | 0.438 |
| LAMA3 | 904 | 0.459 |
| COL7A1 | 904 | 0.499 |
| DSG4 | 301 | 0.503 |
| TGM1 | 442 | 0.504 |
| DDB2 | 768 | 0.522 |
| TMC6 | 252 | 0.541 |
| COL17A1 | 900 | 0.570 |
| MPLKIP | 226 | 0.606 |
| KRT81 | 227 | 0.606 |
| EDA | 567 | 0.667 |
| ADAR | 566 | 0.708 |
| GJB4 | 150 | 0.750 |
| FLCN | 0 | 0.782 |
| GJB6 | 268 | 0.786 |
| EDAR | 279 | 0.795 |
| TMC8 | 0 | 0.819 |
| GTF2H5 | 177 | 0.843 |
| GJB3 | 177 | 0.859 |
| MLPH | 167 | 0.875 |
| POLH | 422 | 0.895 |
| ERCC3 | 721 | 0.899 |
| ERCC2 | 815 | 0.902 |
| DSG1 | 241 | 0.910 |
| ERCC5 | 330 | 0.980 |
| DKC1 | 483 | 0.982 |
| ERCC4 | 347 | 0.988 |
| PLEC | 326 | 0.997 |

1. Disease genes of developmental

| **Gene symbol** | **MIS** | **P-value** |
| --- | --- | --- |
| SIX3 | 996 | <0.001 |
| LHCGR | 979 | <0.001 |
| TSC1 | 999 | <0.001 |
| INSR | 999 | <0.001 |
| PTPN11 | 999 | <0.001 |
| HSD17B3 | 979 | <0.001 |
| CYP1B1 | 971 | 0.018 |
| MKKS | 987 | 0.038 |
| ZEB2 | 928 | 0.052 |
| TWIST1 | 948 | 0.060 |
| PAX6 | 990 | 0.069 |
| MECP2 | 921 | 0.077 |
| NSD1 | 874 | 0.082 |
| SHH | 997 | 0.083 |
| T | 824 | 0.087 |
| CCL2 | 974 | 0.098 |
| TRPS1 | 833 | 0.103 |
| IGF1 | 996 | 0.104 |
| IRF6 | 913 | 0.104 |
| CDKL5 | 704 | 0.113 |
| AMH | 929 | 0.115 |
| ZIC2 | 863 | 0.132 |
| UBE3A | 948 | 0.139 |
| TBX1 | 830 | 0.140 |
| GATA6 | 923 | 0.153 |
| LEMD3 | 724 | 0.156 |
| TBX5 | 871 | 0.192 |
| AMHR2 | 843 | 0.258 |
| NKX2-6 | 575 | 0.271 |
| NSDHL | 684 | 0.272 |
| FUZ | 418 | 0.286 |
| FGFR2 | 945 | 0.287 |
| NKX2-5 | 947 | 0.292 |
| MIF | 649 | 0.325 |
| MTHFR | 720 | 0.347 |
| MED13L | 916 | 0.350 |
| EZH2 | 954 | 0.402 |
| PITX2 | 822 | 0.485 |
| GDF1 | 463 | 0.495 |
| MSX1 | 709 | 0.553 |
| TPM2 | 901 | 0.555 |
| EZH1 | 636 | 0.562 |
| MTRR | 440 | 0.589 |
| TCOF1 | 341 | 0.620 |
| CFC1 | 258 | 0.665 |
| MTR | 481 | 0.673 |
| TNNI2 | 513 | 0.716 |
| TNNT3 | 418 | 0.734 |
| VANGL1 | 422 | 0.752 |
| TBX22 | 223 | 0.837 |
| ATR | 896 | 0.849 |
| NODAL | 372 | 0.869 |
| MYH3 | 459 | 0.971 |
| ESCO2 | 228 | 0.981 |
| NIPBL | 168 | 0.997 |

1. Disease genes of ear nose throat

| **Gene symbol** | **MIS** | **P-value** |
| --- | --- | --- |
| POU3F4 | 945 | <0.001 |
| KCNJ10 | 790 | 0.065 |
| EYA4 | 885 | 0.105 |
| CTSC | 623 | 0.155 |
| MYO7A | 623 | 0.172 |
| OTOA | 407 | 0.219 |
| WFS1 | 634 | 0.247 |
| MYH14 | 954 | 0.268 |
| CDH23 | 608 | 0.295 |
| KCNQ4 | 918 | 0.316 |
| DSPP | 519 | 0.338 |
| COL11A2 | 568 | 0.371 |
| GJB2 | 578 | 0.375 |
| POU4F3 | 475 | 0.417 |
| DIAPH1 | 636 | 0.440 |
| GRHL2 | 607 | 0.441 |
| USH1C | 495 | 0.460 |
| MYH9 | 791 | 0.488 |
| TAS2R38 | 158 | 0.511 |
| ESPN | 415 | 0.565 |
| FOXI1 | 457 | 0.593 |
| ATP2B2 | 488 | 0.658 |
| TMC1 | 288 | 0.680 |
| TECTA | 288 | 0.695 |
| COCH | 225 | 0.696 |
| PCDH15 | 329 | 0.724 |
| SLC26A4 | 469 | 0.725 |
| GJB6 | 268 | 0.786 |
| DFNB31 | 271 | 0.792 |
| OTOF | 264 | 0.812 |
| TMIE | 0 | 0.830 |
| GJB3 | 177 | 0.859 |
| TMPRSS3 | 228 | 0.872 |
| STRC | 151 | 0.894 |
| MYO1A | 415 | 0.909 |
| MYO15A | 324 | 0.955 |
| MYO6 | 370 | 0.970 |
| MYO3A | 324 | 0.983 |
| ACTG1 | 539 | 0.999 |

1. Disease genes of endocrine

| **Gene symbol** | **MIS** | **P-value** |
| --- | --- | --- |
| RETN | 981 | <0.001 |
| INS | 999 | <0.001 |
| FSHB | 999 | <0.001 |
| IL6 | 999 | <0.001 |
| PPP1R3A | 954 | <0.001 |
| PPARG | 999 | <0.001 |
| CYP11B1 | 973 | <0.001 |
| STAT5B | 999 | <0.001 |
| NEUROD1 | 994 | <0.001 |
| LIPC | 984 | <0.001 |
| IRS1 | 999 | <0.001 |
| HNF4A | 999 | <0.001 |
| MC2R | 998 | <0.001 |
| AVPR2 | 804 | <0.001 |
| KCNJ11 | 999 | <0.001 |
| ENPP1 | 979 | <0.001 |
| CYP17A1 | 994 | <0.001 |
| PTPN1 | 999 | <0.001 |
| AR | 999 | <0.001 |
| IRS2 | 999 | <0.001 |
| AVP | 997 | <0.001 |
| IGF2BP2 | 989 | <0.001 |
| ABCC8 | 999 | <0.001 |
| CAPN10 | 923 | <0.001 |
| SSTR5 | 968 | 0.013 |
| PAX8 | 954 | 0.014 |
| GCK | 989 | 0.017 |
| TSHR | 980 | 0.017 |
| TBX19 | 937 | 0.017 |
| SLC2A4 | 994 | 0.026 |
| PAX4 | 925 | 0.030 |
| STAR | 942 | 0.032 |
| HGF | 992 | 0.033 |
| BMP15 | 699 | 0.034 |
| HNF1A | 993 | 0.036 |
| GPD2 | 924 | 0.038 |
| HMGA1 | 984 | 0.041 |
| GCGR | 960 | 0.043 |
| GNAS | 988 | 0.061 |
| TPO | 923 | 0.071 |
| TG | 928 | 0.073 |
| THRB | 972 | 0.077 |
| CASR | 936 | 0.080 |
| PROP1 | 746 | 0.083 |
| MAPK8IP1 | 974 | 0.084 |
| ITPR3 | 981 | 0.084 |
| AKT2 | 994 | 0.094 |
| FSHR | 925 | 0.095 |
| CTLA4 | 987 | 0.097 |
| FOXP3 | 984 | 0.100 |
| MRAP | 725 | 0.101 |
| CDKAL1 | 773 | 0.106 |
| FOXE1 | 700 | 0.107 |
| SLC30A8 | 772 | 0.108 |
| MTNR1B | 919 | 0.109 |
| PTH | 969 | 0.128 |
| HESX1 | 687 | 0.146 |
| AQP2 | 915 | 0.152 |
| DHH | 889 | 0.178 |
| NR3C2 | 616 | 0.183 |
| HNF1B | 725 | 0.208 |
| POR | 687 | 0.210 |
| SLC5A5 | 624 | 0.223 |
| AIP | 628 | 0.241 |
| WFS1 | 634 | 0.247 |
| TCF7L2 | 965 | 0.253 |
| PTF1A | 667 | 0.257 |
| GNRHR | 925 | 0.274 |
| SCNN1G | 836 | 0.317 |
| PTPN22 | 634 | 0.318 |
| CACNA1S | 832 | 0.338 |
| SCNN1A | 840 | 0.339 |
| SCNN1B | 642 | 0.392 |
| DUOX2 | 556 | 0.403 |
| TCF4 | 846 | 0.409 |
| GATA3 | 909 | 0.460 |
| OAS1 | 901 | 0.536 |
| STX16 | 681 | 0.629 |
| TBCE | 351 | 0.705 |
| SYCP3 | 263 | 0.810 |
| LIPH | 0 | 0.865 |
| FGD2 | 206 | 0.931 |
| SUMO4 | 394 | 0.961 |
| TTF2 | 0 | 0.993 |

1. Disease genes of gastrointestinal

| **Gene symbol** | **MIS** | **P-value** |
| --- | --- | --- |
| IL6 | 999 | <0.001 |
| ABCB4 | 945 | <0.001 |
| HGF | 992 | 0.033 |
| HSD3B7 | 972 | 0.033 |
| EDNRB | 988 | 0.039 |
| ABCB11 | 871 | 0.095 |
| RET | 992 | 0.096 |
| SAR1B | 973 | 0.107 |
| BAAT | 900 | 0.123 |
| SLC26A3 | 814 | 0.126 |
| FGF10 | 940 | 0.127 |
| UGT1A1 | 972 | 0.179 |
| CFTR | 969 | 0.200 |
| ATP8B1 | 564 | 0.208 |
| NOD2 | 889 | 0.209 |
| TJP2 | 910 | 0.211 |
| SEC63 | 773 | 0.296 |
| PRKCSH | 538 | 0.339 |
| CTRC | 418 | 0.342 |
| SPINK1 | 503 | 0.382 |
| KRT8 | 602 | 0.421 |
| KRT18 | 603 | 0.457 |
| EPHX1 | 505 | 0.632 |
| PRSS1 | 313 | 0.743 |
| VPS33B | 219 | 0.989 |
| CIRH1A | 264 | 0.993 |

1. Disease genes of hematological

| **Gene symbol** | **MIS** | **P-value** |
| --- | --- | --- |
| CFHR1 | 992 | <0.001 |
| CR1 | 998 | <0.001 |
| CFHR3 | 970 | <0.001 |
| CFH | 999 | <0.001 |
| JAK2 | 999 | <0.001 |
| A4GALT | 900 | 0.010 |
| THPO | 990 | 0.022 |
| CD36 | 995 | 0.023 |
| APOE | 997 | 0.030 |
| AMN | 937 | 0.031 |
| RUNX1 | 995 | 0.031 |
| IFNG | 994 | 0.032 |
| CPN1 | 829 | 0.037 |
| FOXC2 | 891 | 0.040 |
| PDGFRA | 997 | 0.046 |
| MPL | 963 | 0.053 |
| FGA | 983 | 0.057 |
| TPO | 923 | 0.071 |
| GSS | 969 | 0.076 |
| EPOR | 998 | 0.078 |
| CUBN | 951 | 0.089 |
| GIF | 825 | 0.096 |
| AQP3 | 877 | 0.099 |
| ANKRD26 | 576 | 0.102 |
| AQP1 | 677 | 0.104 |
| GATA1 | 979 | 0.107 |
| GGCX | 920 | 0.108 |
| ELANE | 922 | 0.110 |
| SLC19A2 | 507 | 0.122 |
| SH2B3 | 916 | 0.131 |
| AQP1 | 799 | 0.148 |
| GP9 | 900 | 0.166 |
| TF | 957 | 0.167 |
| GCLC | 850 | 0.180 |
| GP1BB | 900 | 0.180 |
| SLC4A1 | 800 | 0.190 |
| BSG | 960 | 0.193 |
| UNC13D | 571 | 0.212 |
| FLT4 | 944 | 0.224 |
| GP1BA | 900 | 0.231 |
| RHAG | 561 | 0.243 |
| ITGA2B | 932 | 0.246 |
| ADAMTS13 | 548 | 0.246 |
| ACHE | 741 | 0.247 |
| VKORC1 | 0 | 0.309 |
| NT5C3A | 902 | 0.349 |
| VKORC1 | 336 | 0.379 |
| CP | 620 | 0.397 |
| LBR | 659 | 0.415 |
| HBB | 590 | 0.416 |
| ATRX | 642 | 0.461 |
| CYP2A6 | 915 | 0.465 |
| MYH9 | 791 | 0.488 |
| VHL | 911 | 0.505 |
| F9 | 445 | 0.517 |
| EPX | 457 | 0.538 |
| CDAN1 | 349 | 0.555 |
| FGB | 459 | 0.563 |
| WAS | 576 | 0.566 |
| ITGB3 | 828 | 0.566 |
| MASTL | 336 | 0.603 |
| GCNT2 | 398 | 0.673 |
| CYP2C9 | 901 | 0.695 |
| ABCB7 | 318 | 0.785 |
| FHL3 | 336 | 0.816 |
| LMAN1 | 281 | 0.960 |
| RPS14 | 759 | 0.989 |
| RPS19 | 461 | 0.992 |

1. Disease genes of immunological

| **Gene symbol** | **MIS** | **P-value** |
| --- | --- | --- |
| CEBPE | 858 | <0.001 |
| MC3R | 991 | <0.001 |
| IL6 | 999 | <0.001 |
| ITGB2 | 999 | <0.001 |
| STAT1 | 999 | <0.001 |
| JAK3 | 999 | <0.001 |
| CISH | 989 | 0.024 |
| ICOS | 803 | 0.025 |
| HLA-DRB1 | 921 | 0.031 |
| C4A | 919 | 0.031 |
| IFNG | 994 | 0.032 |
| HGF | 992 | 0.033 |
| TLR5 | 878 | 0.046 |
| TLR2 | 992 | 0.052 |
| SLC11A1 | 863 | 0.054 |
| MS4A2 | 955 | 0.059 |
| IL10 | 979 | 0.069 |
| SELP | 950 | 0.074 |
| IL21R | 837 | 0.077 |
| CCL3 | 955 | 0.079 |
| NCF1 | 991 | 0.082 |
| IFNGR2 | 910 | 0.093 |
| CCL2 | 974 | 0.098 |
| IFNGR1 | 955 | 0.106 |
| FCGR2B | 916 | 0.107 |
| HAVCR1 | 581 | 0.112 |
| PLA2G7 | 929 | 0.114 |
| IL12RB1 | 939 | 0.114 |
| TAP1 | 570 | 0.126 |
| KIR3DS1 | 602 | 0.126 |
| TNFRSF13B | 863 | 0.131 |
| CXCR4 | 962 | 0.131 |
| SPINK5 | 648 | 0.133 |
| IL4R | 976 | 0.144 |
| IL12B | 952 | 0.144 |
| TNFRSF1A | 995 | 0.158 |
| AIRE | 800 | 0.159 |
| CCL11 | 959 | 0.165 |
| HLA-C | 931 | 0.166 |
| CYBB | 808 | 0.181 |
| DNASE1 | 506 | 0.186 |
| BTK | 905 | 0.195 |
| CD209 | 864 | 0.195 |
| IL2RG | 942 | 0.216 |
| SERPING1 | 922 | 0.219 |
| IL13 | 924 | 0.245 |
| KIR3DL1 | 239 | 0.248 |
| CD40 | 972 | 0.258 |
| CD40LG | 925 | 0.268 |
| NCF2 | 942 | 0.279 |
| CYBA | 898 | 0.282 |
| IRGM | 378 | 0.288 |
| PTPN22 | 634 | 0.318 |
| CASP8 | 977 | 0.342 |
| FAS | 916 | 0.348 |
| CXCL12 | 959 | 0.350 |
| TIRAP | 836 | 0.359 |
| TAP2 | 315 | 0.376 |
| CCL3L1 | 383 | 0.387 |
| TLR3 | 913 | 0.394 |
| ATM | 984 | 0.438 |
| MVK | 563 | 0.439 |
| PTPRZ1 | 499 | 0.444 |
| TAP2 | 315 | 0.452 |
| MRE11A | 958 | 0.457 |
| NLRP3 | 512 | 0.469 |
| CD8A | 944 | 0.502 |
| CXCR1 | 905 | 0.508 |
| ADA | 709 | 0.553 |
| RAG1 | 573 | 0.558 |
| TAPBP | 267 | 0.560 |
| MEFV | 418 | 0.564 |
| WAS | 576 | 0.566 |
| RAG2 | 548 | 0.573 |
| SP110 | 232 | 0.591 |
| RFX5 | 468 | 0.601 |
| CX3CR1 | 440 | 0.613 |
| RAC2 | 942 | 0.635 |
| IRAK4 | 654 | 0.641 |
| PARK2 | 792 | 0.685 |
| RFXANK | 358 | 0.699 |
| CASP10 | 674 | 0.713 |
| LIG4 | 591 | 0.718 |
| AICDA | 342 | 0.741 |
| UNG | 394 | 0.769 |
| BANK1 | 217 | 0.790 |
| MPO | 181 | 0.810 |
| RFXAP | 0 | 0.826 |
| PHF11 | 427 | 0.828 |
| DCLRE1C | 289 | 0.898 |
| TREX1 | 207 | 0.925 |

1. Disease genes of metabolic

| **Gene symbol** | **MIS** | **P-value** |
| --- | --- | --- |
| CETP | 987 | <0.001 |
| APOA5 | 989 | <0.001 |
| GHR | 999 | <0.001 |
| APOB | 999 | <0.001 |
| APOA1 | 999 | <0.001 |
| CYP27A1 | 973 | <0.001 |
| MLYCD | 913 | <0.001 |
| LCAT | 999 | <0.001 |
| SLC5A1 | 988 | <0.001 |
| ABCG8 | 985 | <0.001 |
| PPP1R3A | 954 | <0.001 |
| PPARG | 999 | <0.001 |
| PCSK9 | 999 | <0.001 |
| MPI | 988 | <0.001 |
| TALDO1 | 998 | <0.001 |
| HLCS | 997 | <0.001 |
| DDC | 988 | <0.001 |
| APOA2 | 997 | <0.001 |
| LDLRAP1 | 990 | <0.001 |
| ABCA1 | 999 | <0.001 |
| ALPL | 969 | <0.001 |
| H6PD | 996 | <0.001 |
| GK | 950 | <0.001 |
| PDX1 | 995 | <0.001 |
| ABCC8 | 999 | <0.001 |
| DBH | 986 | <0.001 |
| NAGA | 940 | <0.001 |
| LDLR | 999 | <0.001 |
| APOC2 | 983 | <0.001 |
| RPIA | 972 | 0.015 |
| GCK | 989 | 0.017 |
| CPT1A | 993 | 0.017 |
| CPT2 | 971 | 0.017 |
| FMO3 | 940 | 0.019 |
| SCD | 975 | 0.020 |
| GNS | 914 | 0.021 |
| D2HGDH | 749 | 0.025 |
| CYP11B2 | 965 | 0.025 |
| SLC17A5 | 833 | 0.028 |
| SLC2A1 | 935 | 0.030 |
| AMN | 937 | 0.031 |
| BSCL2 | 841 | 0.031 |
| AGPAT2 | 979 | 0.031 |
| HAL | 916 | 0.036 |
| USF1 | 951 | 0.036 |
| SLC2A2 | 975 | 0.037 |
| PHEX | 942 | 0.038 |
| LIPI | 747 | 0.040 |
| ABCG5 | 847 | 0.044 |
| SLC22A5 | 924 | 0.046 |
| MANBA | 749 | 0.051 |
| GBE1 | 994 | 0.052 |
| MCCC2 | 866 | 0.055 |
| PYGM | 949 | 0.061 |
| IDUA | 908 | 0.066 |
| PPOX | 744 | 0.068 |
| GSS | 969 | 0.076 |
| MAN2B1 | 700 | 0.081 |
| L2HGDH | 807 | 0.082 |
| NPC2 | 826 | 0.089 |
| GALT | 800 | 0.095 |
| GLUD1 | 944 | 0.097 |
| ABHD5 | 908 | 0.102 |
| HSD17B4 | 969 | 0.106 |
| HGD | 668 | 0.110 |
| ALG2 | 796 | 0.110 |
| HMGCS2 | 978 | 0.114 |
| MGAT2 | 810 | 0.116 |
| NPC1 | 813 | 0.123 |
| ACAT1 | 979 | 0.124 |
| ECM1 | 517 | 0.125 |
| MMAB | 750 | 0.129 |
| DHCR24 | 919 | 0.132 |
| HEXB | 900 | 0.137 |
| ACADVL | 902 | 0.137 |
| HEXA | 905 | 0.139 |
| ALDH4A1 | 928 | 0.139 |
| HYAL1 | 183 | 0.142 |
| GRHPR | 905 | 0.152 |
| PHKB | 835 | 0.152 |
| MMAA | 689 | 0.154 |
| SLC7A7 | 553 | 0.159 |
| GYS2 | 956 | 0.166 |
| GNMT | 856 | 0.166 |
| ALG9 | 499 | 0.168 |
| ITIH4 | 899 | 0.171 |
| UGT1A1 | 972 | 0.179 |
| SLC6A19 | 539 | 0.180 |
| CPS1 | 974 | 0.180 |
| ARG1 | 920 | 0.188 |
| PRODH | 821 | 0.190 |
| MCOLN1 | 662 | 0.192 |
| GALK1 | 689 | 0.198 |
| AUH | 695 | 0.205 |
| LMNA | 881 | 0.207 |
| ATIC | 961 | 0.208 |
| ALG8 | 609 | 0.215 |
| ACADM | 950 | 0.216 |
| BCKDHA | 803 | 0.216 |
| MCCC1 | 824 | 0.217 |
| BTD | 472 | 0.218 |
| ALDH3A2 | 923 | 0.220 |
| ALG6 | 758 | 0.220 |
| MUT | 854 | 0.228 |
| GNPTAB | 416 | 0.240 |
| NEU1 | 192 | 0.244 |
| WFS1 | 634 | 0.247 |
| BCKDHB | 801 | 0.249 |
| PCCB | 768 | 0.257 |
| PANK2 | 446 | 0.258 |
| GBA | 585 | 0.262 |
| ADSL | 934 | 0.263 |
| ASS1 | 911 | 0.264 |
| EPHX2 | 508 | 0.273 |
| PCCA | 826 | 0.274 |
| NAGS | 800 | 0.279 |
| CAV3 | 840 | 0.280 |
| IVD | 632 | 0.280 |
| UROS | 485 | 0.284 |
| BCKDHA | 806 | 0.285 |
| GALT | 511 | 0.295 |
| ETFB | 683 | 0.296 |
| GNE | 677 | 0.297 |
| ABCC2 | 720 | 0.303 |
| MADD | 816 | 0.309 |
| FTCD | 800 | 0.312 |
| XDH | 810 | 0.316 |
| ETFA | 757 | 0.329 |
| DPM1 | 703 | 0.334 |
| ALG1 | 640 | 0.338 |
| GCDH | 657 | 0.340 |
| ETFDH | 604 | 0.345 |
| MTHFR | 720 | 0.347 |
| SLC35C1 | 562 | 0.353 |
| ACADS | 696 | 0.354 |
| DPAGT1 | 713 | 0.355 |
| ASPA | 803 | 0.359 |
| GAA | 653 | 0.378 |
| PC | 891 | 0.378 |
| CP | 620 | 0.397 |
| HSD17B10 | 438 | 0.406 |
| FH | 758 | 0.414 |
| TFR2 | 459 | 0.421 |
| HFE | 450 | 0.437 |
| MPDU1 | 416 | 0.450 |
| GLDC | 631 | 0.457 |
| ETHE1 | 286 | 0.479 |
| DBT | 625 | 0.491 |
| SMPD1 | 907 | 0.497 |
| GALE | 507 | 0.497 |
| COG7 | 429 | 0.498 |
| CTH | 679 | 0.500 |
| SLC40A1 | 398 | 0.515 |
| ALG3 | 472 | 0.520 |
| MCM6 | 924 | 0.521 |
| ATP7B | 456 | 0.563 |
| AGXT | 522 | 0.586 |
| MTRR | 440 | 0.589 |
| ABCD1 | 293 | 0.628 |
| LAMP2 | 566 | 0.648 |
| OTC | 489 | 0.671 |
| GCSH | 263 | 0.671 |
| MTR | 481 | 0.673 |
| PRODH2 | 321 | 0.674 |
| PHGDH | 457 | 0.694 |
| SUOX | 365 | 0.702 |
| SLC25A13 | 235 | 0.705 |
| AASS | 297 | 0.719 |
| SUMF1 | 282 | 0.720 |
| ALG12 | 341 | 0.728 |
| HPRT1 | 523 | 0.759 |
| BCS1L | 516 | 0.775 |
| SGSH | 207 | 0.776 |
| PMM2 | 360 | 0.778 |
| AMT | 309 | 0.797 |
| DMGDH | 424 | 0.809 |
| OCRL | 869 | 0.837 |
| MOCS1 | 379 | 0.839 |
| ASL | 357 | 0.842 |
| OPA3 | 177 | 0.842 |
| ALG9 | 198 | 0.862 |
| RP1 | 219 | 0.880 |
| B4GALT1 | 227 | 0.915 |
| SLC25A15 | 159 | 0.942 |

1. Disease genes of multiple

| **Gene symbol** | **MIS** | **P-value** |
| --- | --- | --- |
| BBS9 | 999 | <0.001 |
| BBS2 | 999 | <0.001 |
| CREBBP | 999 | <0.001 |
| TYR | 963 | <0.001 |
| LIFR | 999 | <0.001 |
| BBS7 | 999 | <0.001 |
| BBS4 | 998 | <0.001 |
| NDUFAF2 | 982 | <0.001 |
| TSC1 | 999 | <0.001 |
| INSR | 999 | <0.001 |
| BBS12 | 991 | <0.001 |
| ARL6 | 980 | <0.001 |
| PTPN11 | 999 | <0.001 |
| VPS13B | 885 | <0.001 |
| PTEN | 999 | <0.001 |
| CCDC28B | 846 | <0.001 |
| GRIP1 | 998 | <0.001 |
| PAX2 | 992 | <0.001 |
| BBS1 | 718 | <0.001 |
| GPC3 | 990 | 0.009 |
| BBS5 | 998 | 0.012 |
| DHCR7 | 984 | 0.012 |
| TTC8 | 997 | 0.012 |
| BBS5 | 892 | 0.012 |
| FOXL2 | 967 | 0.018 |
| CRLF1 | 686 | 0.018 |
| BBS1 | 998 | 0.022 |
| SALL4 | 985 | 0.028 |
| JUP | 987 | 0.035 |
| MKKS | 987 | 0.038 |
| BBS10 | 980 | 0.038 |
| CHD7 | 957 | 0.038 |
| EDNRB | 988 | 0.039 |
| TFAP2B | 845 | 0.046 |
| CEP290 | 954 | 0.052 |
| HSPG2 | 989 | 0.054 |
| RPS6KA3 | 996 | 0.054 |
| SHANK3 | 913 | 0.057 |
| MITF | 972 | 0.058 |
| GNAS | 988 | 0.061 |
| DLX3 | 832 | 0.068 |
| NSD1 | 874 | 0.082 |
| FOXP3 | 984 | 0.100 |
| SNAI2 | 971 | 0.102 |
| IRF6 | 913 | 0.104 |
| GATA1 | 979 | 0.107 |
| FREM2 | 535 | 0.113 |
| GLI3 | 976 | 0.126 |
| TBX1 | 830 | 0.140 |
| EGR2 | 933 | 0.142 |
| FLNA | 919 | 0.143 |
| RASA1 | 993 | 0.144 |
| HESX1 | 687 | 0.146 |
| NDN | 709 | 0.150 |
| ZMPSTE24 | 780 | 0.150 |
| CTSC | 623 | 0.155 |
| LEMD3 | 724 | 0.156 |
| PTCH1 | 973 | 0.163 |
| SDS | 800 | 0.164 |
| SALL1 | 846 | 0.166 |
| TP63 | 871 | 0.166 |
| UGT1A1 | 972 | 0.179 |
| MKS1 | 724 | 0.180 |
| CDKN1C | 947 | 0.181 |
| FRAS1 | 524 | 0.183 |
| AHI1 | 802 | 0.194 |
| LMX1B | 751 | 0.201 |
| NUBPL | 625 | 0.205 |
| NOD2 | 889 | 0.209 |
| WRN | 987 | 0.211 |
| TRIM37 | 556 | 0.217 |
| SLC12A1 | 700 | 0.222 |
| TRIM32 | 815 | 0.225 |
| PAX3 | 875 | 0.229 |
| GFAP | 576 | 0.233 |
| WNT3 | 932 | 0.239 |
| WT1 | 926 | 0.246 |
| PEX1 | 564 | 0.247 |
| HRAS | 995 | 0.251 |
| MYCN | 886 | 0.254 |
| FGFR1 | 989 | 0.257 |
| BSND | 396 | 0.261 |
| TMEM67 | 699 | 0.265 |
| TAZ | 576 | 0.267 |
| MGP | 583 | 0.268 |
| COL18A1 | 927 | 0.270 |
| PRKAR1A | 963 | 0.274 |
| FGFR2 | 945 | 0.287 |
| CDH23 | 608 | 0.295 |
| SMS | 609 | 0.305 |
| NOG | 912 | 0.313 |
| SKI | 901 | 0.319 |
| LYST | 487 | 0.331 |
| KCNQ1 | 922 | 0.340 |
| JAG1 | 873 | 0.345 |
| DYM | 457 | 0.352 |
| KCNJ1 | 646 | 0.360 |
| ROR2 | 866 | 0.366 |
| COL11A2 | 568 | 0.371 |
| GJB2 | 578 | 0.375 |
| MPZ | 511 | 0.375 |
| AAAS | 900 | 0.384 |
| FANCA | 946 | 0.393 |
| CACNA1C | 866 | 0.394 |
| SEMA3E | 383 | 0.394 |
| CLRN1 | 360 | 0.396 |
| BRCA2 | 947 | 0.415 |
| DSP | 654 | 0.438 |
| CLDN1 | 632 | 0.444 |
| ATP7A | 563 | 0.451 |
| GPR98 | 370 | 0.451 |
| LAMA3 | 904 | 0.459 |
| USH1C | 495 | 0.460 |
| ATRX | 642 | 0.461 |
| PHF6 | 370 | 0.467 |
| FOXRED1 | 495 | 0.469 |
| NLRP3 | 512 | 0.469 |
| DNMT3B | 890 | 0.476 |
| PITX2 | 822 | 0.485 |
| MYH9 | 791 | 0.488 |
| CLCNKB | 540 | 0.490 |
| COL11A1 | 900 | 0.493 |
| TBX3 | 570 | 0.504 |
| PMP22 | 450 | 0.525 |
| ARX | 462 | 0.526 |
| MYH8 | 789 | 0.527 |
| FANCB | 349 | 0.540 |
| CUL7 | 720 | 0.544 |
| COL2A1 | 900 | 0.546 |
| PRX | 0 | 0.581 |
| USH1G | 364 | 0.596 |
| BACH1 | 0 | 0.624 |
| MID1 | 245 | 0.624 |
| AGPS | 501 | 0.629 |
| PDZD7 | 243 | 0.640 |
| HOXA11 | 376 | 0.644 |
| AP3B1 | 570 | 0.646 |
| MYOC | 390 | 0.662 |
| CFC1 | 258 | 0.665 |
| USH2A | 439 | 0.677 |
| BCOR | 284 | 0.677 |
| NDUFS1 | 669 | 0.680 |
| NDUFV1 | 645 | 0.683 |
| NDUFAF5 | 342 | 0.701 |
| TBCE | 351 | 0.705 |
| POMT1 | 331 | 0.707 |
| ERCC8 | 752 | 0.712 |
| LIG4 | 591 | 0.718 |
| PCDH15 | 329 | 0.724 |
| ZIC3 | 393 | 0.730 |
| KIAA1279 | 242 | 0.730 |
| RAI1 | 173 | 0.735 |
| PEX7 | 300 | 0.749 |
| NDUFA1 | 499 | 0.752 |
| SDHA | 655 | 0.754 |
| SBDS | 416 | 0.768 |
| BCS1L | 516 | 0.775 |
| HOXA13 | 327 | 0.786 |
| NDUFAF3 | 302 | 0.790 |
| INPP5E | 244 | 0.805 |
| RAB3GAP1 | 266 | 0.805 |
| SDHAF1 | 0 | 0.820 |
| SNRPN | 609 | 0.826 |
| TYMP | 349 | 0.829 |
| ERCC6 | 632 | 0.831 |
| LOR | 307 | 0.834 |
| HPS1 | 167 | 0.852 |
| NDUFB9 | 416 | 0.859 |
| GFM1 | 609 | 0.868 |
| DGUOK | 441 | 0.871 |
| L1CAM | 216 | 0.872 |
| ATPAF2 | 243 | 0.874 |
| DNAI1 | 263 | 0.876 |
| NDUFS4 | 346 | 0.881 |
| PUS1 | 426 | 0.886 |
| POMGNT1 | 194 | 0.890 |
| KIF7 | 298 | 0.891 |
| NDUFS2 | 323 | 0.896 |
| NDUFA11 | 0 | 0.934 |
| NDUFAF1 | 162 | 0.946 |
| NDUFS6 | 242 | 0.949 |
| BRIP1 | 303 | 0.952 |
| NDUFB3 | 179 | 0.956 |
| NDUFAF4 | 158 | 0.960 |
| NDUFV2 | 265 | 0.967 |
| CHM | 316 | 0.970 |
| NDUFS3 | 239 | 0.984 |

1. Disease genes of muscular

| **Gene symbol** | **MIS** | **P-value** |
| --- | --- | --- |
| AR | 999 | <0.001 |
| CPT2 | 971 | 0.017 |
| EMD | 576 | 0.021 |
| BSCL2 | 841 | 0.031 |
| MYOT | 680 | 0.035 |
| CHRND | 749 | 0.035 |
| DYSF | 815 | 0.038 |
| DMD | 991 | 0.041 |
| SCG3 | 905 | 0.043 |
| ATP2A1 | 952 | 0.046 |
| SGCD | 936 | 0.059 |
| SGCB | 932 | 0.082 |
| CRYAB | 800 | 0.122 |
| SGCA | 845 | 0.132 |
| SGCG | 813 | 0.150 |
| LMNA | 881 | 0.207 |
| RYR1 | 907 | 0.219 |
| TRIM32 | 815 | 0.225 |
| CHRNA1 | 900 | 0.234 |
| FKRP | 640 | 0.241 |
| CHAT | 883 | 0.255 |
| MTM1 | 453 | 0.256 |
| SMN2 | 847 | 0.271 |
| CAV3 | 840 | 0.280 |
| GNE | 677 | 0.297 |
| IGHMBP2 | 500 | 0.300 |
| SMN1 | 901 | 0.315 |
| SEPN1 | 514 | 0.321 |
| MUSK | 904 | 0.332 |
| MYH2 | 917 | 0.333 |
| DNM2 | 944 | 0.339 |
| SMAD1 | 964 | 0.378 |
| VCP | 977 | 0.388 |
| GARS | 760 | 0.411 |
| COL6A2 | 904 | 0.423 |
| TNNT1 | 836 | 0.426 |
| CAPN3 | 559 | 0.437 |
| DMPK | 357 | 0.440 |
| COL6A1 | 906 | 0.470 |
| CLCN1 | 540 | 0.489 |
| COL6A3 | 901 | 0.495 |
| SCN4A | 456 | 0.552 |
| ACTA1 | 918 | 0.562 |
| CHRNB1 | 258 | 0.628 |
| POMT1 | 331 | 0.707 |
| NEB | 403 | 0.724 |
| MTMR14 | 197 | 0.740 |
| DES | 372 | 0.764 |
| LARGE | 359 | 0.769 |
| TCAP | 396 | 0.790 |
| RAPSN | 288 | 0.795 |
| FKTN | 270 | 0.813 |
| VAPB | 428 | 0.830 |
| CHRNE | 187 | 0.836 |
| DGUOK | 441 | 0.871 |
| LAMA2 | 306 | 0.911 |
| TTN | 392 | 0.921 |
| PABPN1 | 507 | 0.952 |
| MYH7 | 453 | 0.978 |
| PLEC | 326 | 0.997 |

1. Disease genes of neurological

| **Gene symbol** | **MIS** | **P-value** |
| --- | --- | --- |
| ESR1 | 999 | <0.001 |
| APOA1 | 999 | <0.001 |
| SIX3 | 996 | <0.001 |
| ACE | 999 | <0.001 |
| NDUFAF2 | 982 | <0.001 |
| NOS3 | 999 | <0.001 |
| ATP1A3 | 987 | <0.001 |
| SNCA | 999 | <0.001 |
| MAPT | 999 | <0.001 |
| ATP1A2 | 989 | <0.001 |
| DRD2 | 984 | <0.001 |
| GJC2 | 756 | <0.001 |
| SOX3 | 964 | <0.001 |
| PLAU | 999 | <0.001 |
| PDCD10 | 999 | <0.001 |
| RELN | 999 | <0.001 |
| ACE | 954 | <0.001 |
| NHLRC1 | 998 | 0.011 |
| VLDLR | 998 | 0.011 |
| FGF14 | 888 | 0.015 |
| NDRG1 | 986 | 0.017 |
| SLC6A8 | 584 | 0.018 |
| MLC1 | 900 | 0.018 |
| SLC12A6 | 825 | 0.018 |
| PRPH | 901 | 0.020 |
| ACSL4 | 942 | 0.020 |
| VPS13A | 805 | 0.020 |
| SERPINI1 | 908 | 0.021 |
| EPM2A | 994 | 0.021 |
| NDUFAF6 | 859 | 0.021 |
| TNF | 990 | 0.022 |
| APP | 998 | 0.025 |
| APOE | 997 | 0.030 |
| SORL1 | 928 | 0.030 |
| SYN1 | 941 | 0.030 |
| AMN | 937 | 0.031 |
| HLA-DRB1 | 921 | 0.031 |
| BSCL2 | 841 | 0.031 |
| LRRK2 | 998 | 0.037 |
| THBS2 | 942 | 0.039 |
| NKX2-1 | 982 | 0.040 |
| CILP | 658 | 0.041 |
| LGI1 | 812 | 0.042 |
| GSN | 974 | 0.042 |
| PDCD1 | 994 | 0.043 |
| SOD1 | 971 | 0.047 |
| NEFH | 865 | 0.049 |
| HSPB1 | 996 | 0.053 |
| SLC16A2 | 668 | 0.054 |
| EDNRA | 986 | 0.054 |
| HSPG2 | 989 | 0.054 |
| SCN1A | 830 | 0.056 |
| FGA | 983 | 0.057 |
| KIF1B | 822 | 0.063 |
| GCH1 | 903 | 0.065 |
| HLA-DQB1 | 815 | 0.066 |
| SLC19A3 | 502 | 0.067 |
| SGCE | 906 | 0.068 |
| ASPN | 760 | 0.070 |
| MECP2 | 921 | 0.077 |
| HOXA1 | 824 | 0.078 |
| GJB1 | 832 | 0.079 |
| PLP1 | 842 | 0.081 |
| SHH | 997 | 0.083 |
| NTRK1 | 997 | 0.087 |
| DCX | 855 | 0.090 |
| PNKD | 604 | 0.096 |
| RET | 992 | 0.096 |
| PSEN1 | 996 | 0.103 |
| CSTB | 750 | 0.104 |
| LITAF | 689 | 0.104 |
| KCNA1 | 956 | 0.109 |
| ARFGEF2 | 852 | 0.114 |
| EYA1 | 921 | 0.119 |
| ATXN1 | 934 | 0.121 |
| CST3 | 939 | 0.124 |
| CCM2 | 848 | 0.127 |
| FOXP2 | 806 | 0.134 |
| PER2 | 962 | 0.137 |
| SPG20 | 811 | 0.141 |
| EGR2 | 933 | 0.142 |
| FLNA | 919 | 0.143 |
| KCNMA1 | 923 | 0.145 |
| LYZ | 939 | 0.147 |
| COL9A3 | 910 | 0.149 |
| LRPPRC | 900 | 0.152 |
| CHRNA4 | 864 | 0.153 |
| ATXN3 | 925 | 0.154 |
| PRNP | 928 | 0.156 |
| SNCB | 482 | 0.157 |
| PPP2R2B | 904 | 0.165 |
| PARK7 | 949 | 0.170 |
| TOR1A | 715 | 0.171 |
| APTX | 934 | 0.174 |
| GABRG2 | 901 | 0.174 |
| ITM2B | 907 | 0.180 |
| NIPA1 | 660 | 0.188 |
| SCN9A | 594 | 0.191 |
| ALMS1 | 900 | 0.192 |
| MFN2 | 835 | 0.195 |
| ATL1 | 615 | 0.195 |
| PEX5 | 919 | 0.199 |
| TTPA | 557 | 0.200 |
| SOX10 | 868 | 0.201 |
| PRKCG | 903 | 0.203 |
| SPAST | 720 | 0.212 |
| TPP1 | 665 | 0.214 |
| RYR1 | 907 | 0.219 |
| CACNA1A | 856 | 0.219 |
| PINK1 | 939 | 0.228 |
| TDP1 | 860 | 0.234 |
| ME2 | 833 | 0.247 |
| FTL | 832 | 0.249 |
| PANK2 | 446 | 0.258 |
| GBA | 585 | 0.262 |
| NLGN3 | 535 | 0.265 |
| WNK1 | 846 | 0.270 |
| COL9A2 | 908 | 0.272 |
| HSPD1 | 931 | 0.285 |
| EIF2B5 | 660 | 0.295 |
| SMS | 609 | 0.305 |
| PSEN2 | 926 | 0.315 |
| KIF5A | 790 | 0.316 |
| SCN2A | 693 | 0.321 |
| PDHA1 | 908 | 0.323 |
| CACNA1S | 832 | 0.338 |
| DNM2 | 944 | 0.339 |
| CLN6 | 482 | 0.342 |
| CLN3 | 640 | 0.348 |
| SH3TC2 | 323 | 0.358 |
| GLRA1 | 584 | 0.362 |
| ATXN7 | 806 | 0.365 |
| HSPB8 | 606 | 0.367 |
| ZNF81 | 393 | 0.367 |
| MPZ | 511 | 0.375 |
| SMAD1 | 964 | 0.378 |
| KCNQ3 | 921 | 0.381 |
| CP | 620 | 0.397 |
| ARHGEF10 | 290 | 0.403 |
| COLQ | 499 | 0.404 |
| SBF2 | 459 | 0.405 |
| CLCN2 | 559 | 0.411 |
| GARS | 760 | 0.411 |
| SLC25A22 | 374 | 0.414 |
| APBB2 | 345 | 0.417 |
| MTMR2 | 524 | 0.422 |
| GAN | 609 | 0.423 |
| ARSA | 499 | 0.424 |
| KCNQ2 | 921 | 0.427 |
| PHYH | 406 | 0.429 |
| HFE | 450 | 0.437 |
| CACNB4 | 800 | 0.438 |
| PQBP1 | 321 | 0.443 |
| COL4A1 | 905 | 0.449 |
| ATP7A | 563 | 0.451 |
| PAFAH1B1 | 954 | 0.451 |
| GPR98 | 370 | 0.451 |
| PDC | 457 | 0.461 |
| GDAP1 | 457 | 0.463 |
| KRIT1 | 487 | 0.466 |
| FOXRED1 | 495 | 0.469 |
| KDM5C | 482 | 0.482 |
| COL11A1 | 900 | 0.493 |
| CENPJ | 905 | 0.493 |
| SLC25A19 | 321 | 0.496 |
| DCTN1 | 863 | 0.513 |
| EIF2B1 | 564 | 0.514 |
| CHRNB2 | 388 | 0.517 |
| PMP22 | 450 | 0.525 |
| ARX | 462 | 0.526 |
| CLN5 | 297 | 0.527 |
| TBP | 987 | 0.529 |
| JPH3 | 282 | 0.551 |
| SCN4A | 456 | 0.552 |
| ATN1 | 390 | 0.561 |
| CDK5RAP2 | 900 | 0.581 |
| PPT1 | 440 | 0.587 |
| EIF2B3 | 495 | 0.595 |
| IGBP1 | 644 | 0.613 |
| SPTLC1 | 603 | 0.614 |
| ATXN2 | 477 | 0.614 |
| MR1 | 0 | 0.623 |
| ABCD1 | 293 | 0.628 |
| CLN8 | 381 | 0.656 |
| PRSS12 | 207 | 0.664 |
| PARK2 | 792 | 0.685 |
| NDUFA2 | 648 | 0.692 |
| ROBO3 | 332 | 0.695 |
| HCFC1 | 690 | 0.696 |
| ATCAY | 0 | 0.703 |
| SPG21 | 284 | 0.721 |
| GALC | 359 | 0.738 |
| SDHA | 655 | 0.754 |
| FXN | 414 | 0.754 |
| ASPM | 366 | 0.756 |
| BLMH | 396 | 0.770 |
| ALS2 | 386 | 0.770 |
| IKBKAP | 574 | 0.773 |
| BCS1L | 516 | 0.775 |
| OPHN1 | 451 | 0.777 |
| SNAP29 | 412 | 0.779 |
| SACS | 198 | 0.782 |
| EFHC1 | 227 | 0.783 |
| CRBN | 250 | 0.788 |
| SPG7 | 527 | 0.794 |
| MPO | 181 | 0.810 |
| GPR56 | 203 | 0.814 |
| SCN1B | 167 | 0.824 |
| NDUFA12 | 340 | 0.829 |
| VAPB | 428 | 0.830 |
| POLG | 322 | 0.849 |
| EIF2B4 | 202 | 0.858 |
| SURF1 | 252 | 0.863 |
| L1CAM | 216 | 0.872 |
| CLN3 | 254 | 0.873 |
| SETX | 359 | 0.880 |
| PAK3 | 576 | 0.880 |
| NDUFS4 | 346 | 0.881 |
| PEX12 | 196 | 0.888 |
| NDUFS7 | 340 | 0.897 |
| ARHGEF6 | 703 | 0.899 |
| COX15 | 234 | 0.916 |
| MCPH1 | 193 | 0.933 |
| FTSJ1 | 347 | 0.942 |
| NDUFA10 | 233 | 0.944 |
| TIMM8A | 230 | 0.947 |
| EIF2B2 | 226 | 0.948 |
| NDUFA9 | 200 | 0.957 |
| CHM | 316 | 0.970 |
| NDUFS8 | 216 | 0.975 |
| NDUFS3 | 239 | 0.984 |
| TTF1 | 279 | 0.988 |

1. Disease genes of nutritional

| **Gene symbol** | **MIS** | **P-value** |
| --- | --- | --- |
| UCP1 | 969 | <0.001 |
| POMC | 998 | <0.001 |
| PPARG | 999 | <0.001 |
| AGRP | 999 | <0.001 |
| MC4R | 999 | <0.001 |
| PCSK1 | 970 | <0.001 |
| PPARGC1B | 978 | <0.001 |
| UCP3 | 960 | <0.001 |
| GHRL | 994 | <0.001 |
| ADRB3 | 964 | <0.001 |
| PYY | 993 | <0.001 |
| ENPP1 | 979 | <0.001 |
| NR0B2 | 993 | 0.027 |
| SIM1 | 879 | 0.044 |
| HTR2A | 949 | 0.046 |
| SDC3 | 972 | 0.050 |
| ADRB2 | 972 | 0.097 |
| SLC6A14 | 379 | 0.410 |

1. Disease genes of ophthamological

| **Gene symbol** | **MIS** | **P-value** |
| --- | --- | --- |
| PLG | 999 | <0.001 |
| CFHR1 | 992 | <0.001 |
| FOXE3 | 930 | <0.001 |
| CFHR3 | 970 | <0.001 |
| GJA3 | 905 | 0.014 |
| PITX3 | 937 | 0.014 |
| CYP1B1 | 971 | 0.018 |
| PRPH | 901 | 0.020 |
| APOE | 997 | 0.030 |
| TGFBI | 911 | 0.033 |
| PHOX2A | 828 | 0.055 |
| VSX1 | 682 | 0.062 |
| PAX6 | 990 | 0.069 |
| SAG | 921 | 0.069 |
| TACSTD2 | 587 | 0.076 |
| NDP | 753 | 0.079 |
| OPN1MW | 560 | 0.087 |
| LRP5 | 992 | 0.090 |
| RGS9BP | 900 | 0.102 |
| AIM1 | 658 | 0.112 |
| VCAN | 954 | 0.115 |
| KRT12 | 461 | 0.127 |
| HSF4 | 840 | 0.146 |
| DRD5 | 916 | 0.146 |
| CYP4V2 | 569 | 0.146 |
| PIKFYVE | 956 | 0.147 |
| SOX2 | 978 | 0.152 |
| GJA8 | 619 | 0.154 |
| KRT3 | 515 | 0.173 |
| ABCA4 | 696 | 0.176 |
| TIMP3 | 891 | 0.198 |
| OPN1LW | 508 | 0.210 |
| RGS9 | 792 | 0.216 |
| RPGRIP1 | 572 | 0.227 |
| FBLN5 | 852 | 0.228 |
| ELOVL4 | 590 | 0.231 |
| FOXC1 | 604 | 0.234 |
| FBN1 | 897 | 0.241 |
| EFEMP1 | 570 | 0.244 |
| FTL | 832 | 0.249 |
| PLEKHA1 | 357 | 0.257 |
| ROM1 | 662 | 0.262 |
| HMCN1 | 410 | 0.266 |
| SPATA7 | 394 | 0.274 |
| CRYGC | 405 | 0.305 |
| OPTN | 900 | 0.324 |
| KERA | 840 | 0.327 |
| SLC45A2 | 472 | 0.341 |
| BEST1 | 540 | 0.378 |
| RHO | 841 | 0.382 |
| FZD4 | 932 | 0.388 |
| CRYBA1 | 398 | 0.401 |
| CRYBB2 | 296 | 0.438 |
| CHST6 | 297 | 0.440 |
| PRPH2 | 502 | 0.453 |
| RLBP1 | 520 | 0.464 |
| CNGB3 | 402 | 0.476 |
| PITX2 | 822 | 0.485 |
| NHS | 416 | 0.492 |
| AIPL1 | 440 | 0.498 |
| CA4 | 418 | 0.500 |
| C1QTNF5 | 0 | 0.505 |
| CRYAA | 534 | 0.514 |
| COL8A2 | 682 | 0.521 |
| RPE65 | 576 | 0.561 |
| IMPDH1 | 645 | 0.570 |
| RP9 | 359 | 0.585 |
| CRX | 580 | 0.599 |
| CTDP1 | 900 | 0.612 |
| NYX | 457 | 0.627 |
| PRPF8 | 956 | 0.634 |
| MYOC | 390 | 0.662 |
| PRPF31 | 590 | 0.682 |
| TULP1 | 396 | 0.691 |
| CRYGD | 254 | 0.720 |
| OPA1 | 374 | 0.753 |
| CERKL | 399 | 0.771 |
| PDE6B | 584 | 0.779 |
| GUCY2D | 669 | 0.793 |
| LAMB2 | 472 | 0.794 |
| CNGA3 | 285 | 0.799 |
| CRB1 | 341 | 0.802 |
| RDH5 | 313 | 0.807 |
| OPA3 | 177 | 0.842 |
| POLG | 322 | 0.849 |
| CACNA1F | 356 | 0.868 |
| RP1 | 219 | 0.880 |
| KIF21A | 298 | 0.880 |
| FSCN2 | 167 | 0.932 |
| RPGR | 340 | 0.939 |
| GUCA1A | 288 | 0.963 |
| CHM | 316 | 0.970 |

1. Disease genes of psychiatric

| **Gene symbol** | **MIS** | **P-value** |
| --- | --- | --- |
| DRD4 | 972 | <0.001 |
| SLC6A4 | 961 | <0.001 |
| AKT1 | 999 | <0.001 |
| APOL4 | 742 | <0.001 |
| COMT | 981 | <0.001 |
| BDNF | 999 | <0.001 |
| SLC6A3 | 984 | 0.017 |
| CHI3L1 | 842 | 0.024 |
| APOL2 | 662 | 0.035 |
| HCRT | 967 | 0.043 |
| HTR2A | 949 | 0.046 |
| DAOA | 700 | 0.057 |
| GABBR2 | 902 | 0.069 |
| DRD3 | 925 | 0.081 |
| TPH2 | 815 | 0.121 |
| DLX2 | 836 | 0.145 |
| DRD5 | 916 | 0.146 |
| CHRNA4 | 864 | 0.153 |
| PRNP | 928 | 0.156 |
| PRODH | 821 | 0.190 |
| RTN4R | 788 | 0.192 |
| DISC1 | 866 | 0.214 |
| HTT | 934 | 0.238 |
| NLGN3 | 535 | 0.265 |
| DYX1C1 | 628 | 0.297 |
| MTHFR | 720 | 0.347 |
| DAO | 472 | 0.399 |
| KIAA0319 | 402 | 0.408 |
| CYP2A6 | 915 | 0.465 |
| DTNBP1 | 647 | 0.516 |
| PRODH2 | 321 | 0.674 |

1. Disease genes of renal

| **Gene symbol** | **MIS** | **P-value** |
| --- | --- | --- |
| REN | 999 | <0.001 |
| AGTR1 | 998 | <0.001 |
| ACE | 999 | <0.001 |
| AVPR2 | 804 | <0.001 |
| SLC4A5 | 814 | <0.001 |
| CD2AP | 999 | <0.001 |
| TRPM6 | 836 | <0.001 |
| AGT | 999 | <0.001 |
| ACE | 954 | <0.001 |
| COL4A4 | 929 | 0.016 |
| FOXL2 | 967 | 0.018 |
| FXYD2 | 984 | 0.021 |
| SLC7A9 | 767 | 0.034 |
| COL4A3 | 924 | 0.040 |
| SLC5A2 | 800 | 0.058 |
| INSL3 | 827 | 0.076 |
| UMOD | 576 | 0.105 |
| SLC22A12 | 750 | 0.123 |
| INVS | 854 | 0.131 |
| ACTN4 | 960 | 0.132 |
| NPHS1 | 958 | 0.137 |
| IQCB1 | 671 | 0.157 |
| NPHP3 | 628 | 0.180 |
| CLCN5 | 495 | 0.186 |
| HNF1B | 725 | 0.208 |
| NPHS2 | 775 | 0.219 |
| WT1 | 926 | 0.246 |
| ATP6V0A4 | 917 | 0.270 |
| SLC3A1 | 563 | 0.300 |
| ZNF365 | 323 | 0.305 |
| SCNN1G | 836 | 0.317 |
| CACNA1S | 832 | 0.338 |
| TRPC6 | 894 | 0.340 |
| NPHP4 | 581 | 0.346 |
| PKHD1 | 508 | 0.363 |
| SLC12A3 | 537 | 0.363 |
| SCNN1B | 642 | 0.392 |
| NPHP1 | 565 | 0.457 |
| COL4A5 | 905 | 0.486 |
| SLC4A4 | 507 | 0.490 |
| CTNS | 286 | 0.495 |
| CD151 | 325 | 0.628 |
| CLDN16 | 323 | 0.740 |
| PKD1 | 405 | 0.846 |
| DIAPH2 | 349 | 0.917 |

1. Disease genes of respiratory

| **Gene symbol** | **MIS** | **P-value** |
| --- | --- | --- |
| TSC2 | 999 | <0.001 |
| MC3R | 991 | <0.001 |
| SCGB1A1 | 974 | <0.001 |
| HNMT | 968 | <0.001 |
| TSC1 | 999 | <0.001 |
| PTGDR | 965 | <0.001 |
| BDNF | 999 | <0.001 |
| FLCN | 874 | 0.017 |
| TNF | 990 | 0.022 |
| CISH | 989 | 0.024 |
| IFNG | 994 | 0.032 |
| PHOX2B | 927 | 0.040 |
| FCGR2A | 982 | 0.042 |
| TLR2 | 992 | 0.052 |
| SLC11A1 | 863 | 0.054 |
| SFTPB | 870 | 0.070 |
| HMOX1 | 973 | 0.072 |
| ALOX5 | 879 | 0.081 |
| EDN3 | 939 | 0.087 |
| RET | 992 | 0.096 |
| ADRB2 | 972 | 0.097 |
| CCL2 | 974 | 0.098 |
| MMP1 | 988 | 0.104 |
| IFNGR1 | 955 | 0.106 |
| PLA2G7 | 929 | 0.114 |
| TGFB1 | 993 | 0.130 |
| IL12B | 952 | 0.144 |
| CCL11 | 959 | 0.165 |
| GDNF | 919 | 0.185 |
| CD209 | 864 | 0.195 |
| CFTR | 969 | 0.200 |
| IL13 | 924 | 0.245 |
| SERPINA1 | 929 | 0.253 |
| IRGM | 378 | 0.288 |
| TIRAP | 836 | 0.359 |
| ASCL1 | 901 | 0.369 |
| SCGB3A2 | 295 | 0.425 |
| SFTPA1 | 360 | 0.515 |
| SFTPA2 | 311 | 0.545 |
| SP110 | 232 | 0.591 |
| MUC5B | 824 | 0.616 |
| HLA-G | 375 | 0.637 |
| NPSR1 | 918 | 0.706 |
| FLCN | 0 | 0.782 |
| MUC7 | 290 | 0.783 |
| PHF11 | 427 | 0.828 |
| DNAH5 | 225 | 0.994 |

1. Disease genes of skeletal

| **Gene symbol** | **MIS** | **P-value** |
| --- | --- | --- |
| GHR | 999 | <0.001 |
| GHSR | 994 | <0.001 |
| PRG4 | 834 | <0.001 |
| SOX9 | 983 | 0.014 |
| SCT | 970 | 0.019 |
| SRA1 | 976 | 0.023 |
| GJA1 | 994 | 0.033 |
| THBS2 | 942 | 0.039 |
| CILP | 658 | 0.041 |
| SHOX | 757 | 0.059 |
| ASPN | 760 | 0.070 |
| SHH | 997 | 0.083 |
| WISP3 | 458 | 0.086 |
| MNX1 | 866 | 0.089 |
| IRF6 | 913 | 0.104 |
| OFD1 | 917 | 0.118 |
| GLI3 | 976 | 0.126 |
| TGFB1 | 993 | 0.130 |
| FLNA | 919 | 0.143 |
| COL9A3 | 910 | 0.149 |
| COMP | 840 | 0.150 |
| TP63 | 871 | 0.166 |
| FGFR3 | 980 | 0.183 |
| LMBR1 | 450 | 0.188 |
| CTSK | 786 | 0.247 |
| FBLN1 | 865 | 0.254 |
| FGFR1 | 989 | 0.257 |
| COL9A2 | 908 | 0.272 |
| IHH | 919 | 0.276 |
| EVC | 398 | 0.282 |
| FGFR2 | 945 | 0.287 |
| TBX4 | 545 | 0.291 |
| MSX2 | 880 | 0.292 |
| SOST | 851 | 0.297 |
| NOG | 912 | 0.313 |
| RUNX2 | 958 | 0.328 |
| MESP2 | 507 | 0.345 |
| DYM | 457 | 0.352 |
| ROR2 | 866 | 0.366 |
| MATN3 | 400 | 0.384 |
| LBR | 659 | 0.415 |
| EVC2 | 355 | 0.421 |
| GDF5 | 585 | 0.491 |
| COL11A1 | 900 | 0.493 |
| BMPR1B | 900 | 0.502 |
| FLNB | 784 | 0.516 |
| HOXD13 | 513 | 0.519 |
| EFNB1 | 901 | 0.543 |
| COL2A1 | 900 | 0.546 |
| MSX1 | 709 | 0.553 |
| ACAN | 890 | 0.556 |
| PAX9 | 424 | 0.619 |
| TRAPPC2 | 329 | 0.630 |
| BMP2 | 913 | 0.638 |
| NPR2 | 867 | 0.679 |
| DLL3 | 257 | 0.713 |
| HOXD10 | 296 | 0.819 |
| SLC26A2 | 220 | 0.867 |

1. Disease genes of unclassified

| **Gene symbol** | **MIS** | **P-value** |
| --- | --- | --- |
| PTEN | 999 | <0.001 |
| HTR2A | 949 | 0.046 |
| TAS2R16 | 932 | 0.071 |
| LRP5 | 992 | 0.090 |
| FSHR | 925 | 0.095 |
| GABRA2 | 905 | 0.116 |
| CFTR | 969 | 0.200 |
| ADH1B | 928 | 0.205 |
| SH3BP2 | 852 | 0.254 |
| FGFR2 | 945 | 0.287 |
| RCBTB1 | 497 | 0.455 |
| TSPYL1 | 267 | 0.920 |
| PLOD2 | 216 | 0.936 |
